# The Relationship between the Aberrant Long Non-Coding RNA-Mediated Competitive Endogenous RNA Network and Alzheimer’s Disease Pathogenesis

**DOI:** 10.3390/ijms23158497

**Published:** 2022-07-31

**Authors:** Zhongdi Cai, Kaiyue Zhao, Li Zeng, Mimin Liu, Ting Sun, Zhuorong Li, Rui Liu

**Affiliations:** Institute of Medicinal Biotechnology, Chinese Academy of Medical Sciences and Peking Union Medical College, Beijing 100050, China; zhongdi_cai@163.com (Z.C.); z1017882692@163.com (K.Z.); zengli0106@126.com (L.Z.); lmm161010609@163.com (M.L.); tingsun0330@163.com (T.S.)

**Keywords:** Alzheimer’s disease, ceRNAs, lncRNAs, RNA sequencing, 5×FAD mice

## Abstract

Alzheimer’s disease (AD) is a common neurodegenerative disorder characterized by cognitive dysfunction. The role of long non-coding RNAs (lncRNAs) with the action of competitive endogenous RNA (ceRNA) in AD remains unclear. The present study aimed to identify significantly differentially expressed lncRNAs (SDELs) and establish lncRNA-associated ceRNA networks via RNA sequencing analysis and a quantitative real-time Polymerase Chain Reaction (qPCR) assay using transgenic mice with five familial AD mutations. A total of 53 SDELs in the cortex and 51 SDELs in the hippocampus were identified, including seven core SDELs common to both regions. The functions and pathways were then investigated through the potential target genes of SDELs via Gene Ontology and Kyoto Encyclopedia of Genes and Genomes analyses, which indicate biological effects, action distributions, and pathological transductions associated with AD. Based on the ceRNA hypothesis, integrated ceRNA networks in the cortex and hippocampus of lncRNA-miRNA-mRNA were constructed. The core SDEL-mediated ceRNA relationship was established and the expression of these RNAs was verified by qPCR. The results identified lncRNA ENSMUST00000127786 and highlighted miRNAs and mRNAs as potential key mediators in AD. These findings provide AD-derived lncRNA-mediated ceRNA profiles, and further experimental evidence is needed to confirm these identified ceRNA regulatory relationships.

## 1. Introduction

Alzheimer’s disease (AD) is a complex neurodegenerative disorder with multiple etiologies that accounts for 60–80% of all dementia types. Due to the insidious, progressive, and irreversible nature of AD, the average survival time is about 6 years after diagnosis [1], making AD a leading cause of death in the elderly. At present, only six drugs are approved by the US Food and Drug Administration for treatment of AD. Among these drugs, aducanumab, a monoclonal antibody, is the only one that targets the underlying pathological mechanisms of AD by binding to amyloid-β (Aβ) in the brain to eliminate deposition of Aβ plaques [2]. However, the molecular mechanisms underlying the pathogenesis of AD remain largely unknown. Therefore, the identification of effective prognostic biomarkers and therapeutic targets is of great significance for elucidating the mechanism of AD, which is the basis of diagnosis and drug discovery.

With the development of high-throughput sequencing technology and microarrays, non-coding RNAs (ncRNAs) have been shown to play a role in various neurodegenerative diseases, including AD [3], Parkinson’s disease [4], vascular dementia [5], and amyotrophic lateral sclerosis (ALS) [6]. Long non-coding RNAs (lncRNAs) are a class of ncRNAs greater than 200 nucleotides in length. Prior studies have reported that some lncRNAs specifically expressed in brain tissue are involved in the pathological process of AD, participating in the formation and accumulation of Aβ plaques, neurofibrillary tangles, synaptic dysfunction, inflammatory response, and neuronal death [7]. A form of small ncRNAs within 20 nucleotides in length termed microRNAs (miRNAs) are also evidenced to play crucial roles in the etiology of AD [8]. Since miRNAs have been extensively studied in many diseases, the concept of “miRNA sponges” has been proposed, which is a large proportion of RNA transcripts with miRNA response elements (MREs) act as competitive inhibitors of miRNAs. In recent years, the involvement of lncRNAs in competitive endogenous RNA (ceRNA) interplay between ncRNAs and messenger RNAs (mRNAs) has been proposed. Under the ceRNA mechanism, aberrantly expressed lncRNAs with MRE competitively sequestrate miRNAs and reduce the interaction between miRNA and mRNA, thus attenuating the repression of the downstream mRNAs [9]. Accordingly, the role of lncRNA-mediated ceRNA in AD has received increasing attention. For example, BACE1-antisense transcript (BACE1-AS), an extensively studied lncRNA, has been found to share many MREs with BACE1, leading to the repression of various miRNAs such as miR-107, miR-124, and miR-761 that target BACE1, thereby preventing BACE1 mRNA from being degraded [10]. lncRNA brain-derived neurotrophic factor anti-sense (BDNF-AS), a newly discovered lncRNA in AD, promotes Aβ deposition by functioning as a ceRNA in the miR-9-5p/BACE1 signaling pathway [11]. Despite reports of several ceRNA networks associated with lncRNAs in AD, the current knowledge of the regulatory roles and underlying mechanisms of lncRNA as a ceRNA in AD is limited.

**Figure 1:**
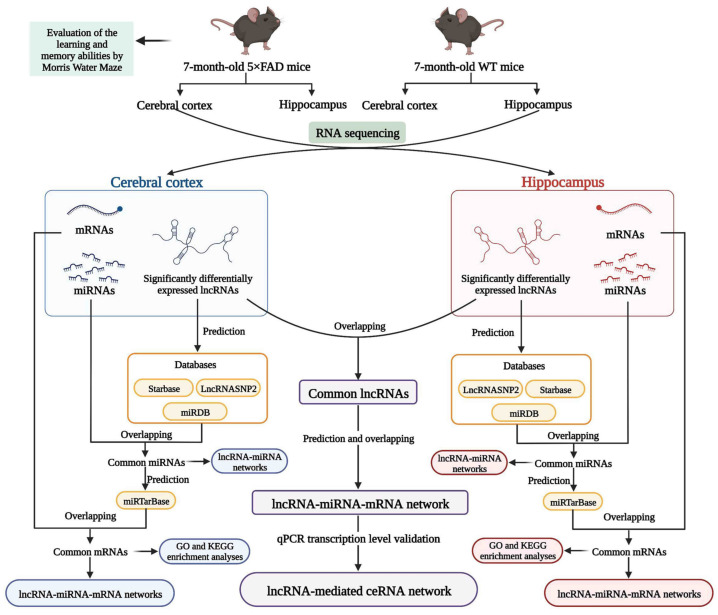
Workflow of the present study. ceRNA: competitive endogenous RNA; GO: gene ontology; KEGG: Kyoto Encyclopedia of Genes and Genomes; lncRNA: long non-coding RNA; miRNA: microRNA; mRNA: messenger RNA; qPCR: quantitative real-time Polymerase Chain Reaction; WT mice: wild-type mice; 5×FAD mice: mice overexpressing neuron-specific transgenes with five familial Alzheimer’s disease mutations. (Created with BioRender.com).

In the present study, mice overexpressing neuron-specific transgenes with five familial AD (5×FAD) mutations, namely Florida (I716V), Swedish (K670N and M671L), and London (V717I) in human amyloid precursor protein (*APP*) and M146L and L286V in human presenilin 1 (*PS1)* [12], were used as a model of AD. Several transcriptomic or proteomic analyses have been performed on the 5×FAD mice, focusing on the alteration profile of protein-coding genes in the cerebral cortex or hippocampus [13,14,15]. Nevertheless, there are still no transcriptional genome studies that have identified the lncRNA-associated ceRNA mechanisms in the brain of 5×FAD mice. Therefore, RNA sequencing (RNA-seq) analyses were performed to discriminate differently expressed lncRNAs and associated ceRNA networks in the cortex and hippocampus of 5×FAD mice relative to wild-type (WT) mice at 7 months of age. Furthermore, common lncRNAs in both brain areas and lncRNA-mediated ceRNA regulatory networks were successfully established and the expression of known lncRNAs was verified, accompanied by corresponding miRNAs and mRNAs in the ceRNA networks (Figure 1). Overall, our findings reveal lncRNA-associated ceRNA regulatory networks and provide new insights for understanding the pathogenic mechanism of AD.

## 2. Results

### 2.1. Spatial Cognition Capability Is Reduced in 5×FAD Mice

**Figure 2:**
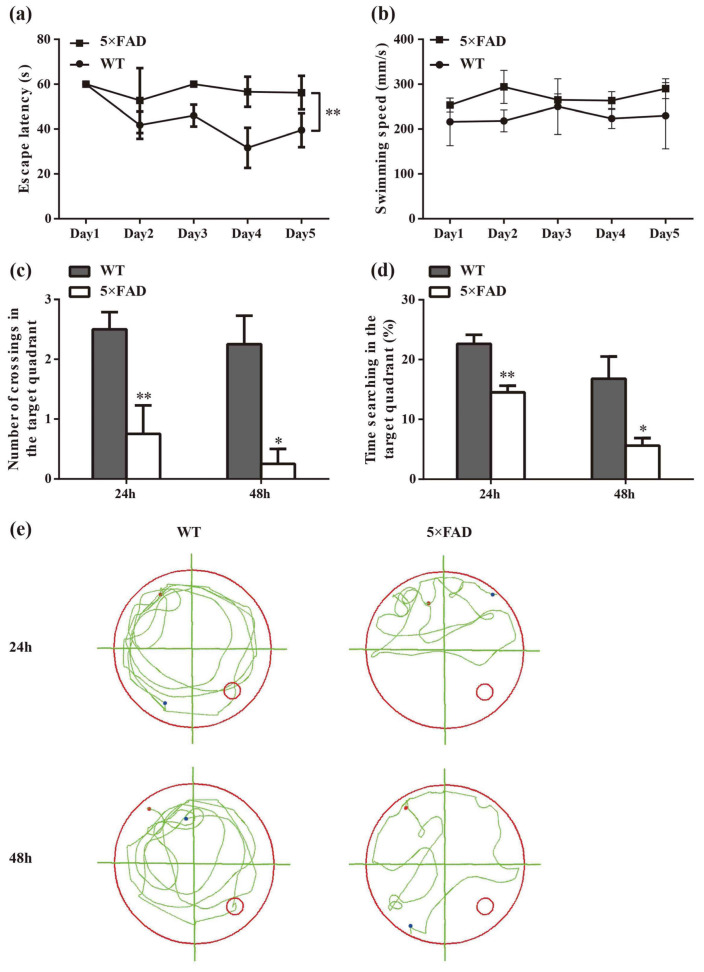
Evaluation of learning and memory abilities in 7-month-old 5×FAD mice compared with age-matched WT mice. (**a**) Comparison of latency to the platform in the spatial learning trial. (**b**) No significant differences were found in swimming speed. (**c**) Comparison of the number of crossings of the platform in the target quadrant in the spatial probe trial. (**d**) Comparison of duration spent in the target quadrant. (**e**) Representative action tracks of 5×FAD and WT mice in the spatial probe trial at 24 h and 48 h after the spatial learning trial. The red dot represents the mouse position at the start of the 60 s timing, and the blue dot represents where the mouse stayed at the endpoint. Data were analyzed using SPSS software, version 25.0 (IBM, Armonk, NY, USA). Comparisons were calculated by repeated measures ANOVA (**a**,**b**) and one-way ANOVA (**c**,**d**). Results are presented as mean ± SD. *n* = 4. * *p* < 0.05, ** *p* < 0.01 vs. WT mice.

In the spatial learning trial, there was a significant difference in latency between the 5×FAD and age-matched WT mice (*p* < 0.01), indicating that 5×FAD mice had a deficit in spatial learning ability (Figure 2a). To rule out that the group difference was due to motor ability, the swimming speed of the mice was recorded. The results showed no difference in swimming speed between the two groups during the five training days (Figure 2b). In the spatial probe trial, the time spent searching in the target quadrant and crossings of the previous platform were significantly reduced in 5×FAD mice compared to WT mice at 24 h and 48 h (Figure 2c,d, *p* < 0.01 and *p* < 0.05, respectively), suggesting impairment of both short- and long-term memory functions. Figure 2e presents a representative roadmap of action tracks for the two groups in the spatial probe trial, demonstrating the non-purposeful and mindless swimming of 5×FAD mice. These results indicate the presence of learning and memory dysfunction in 7-month-old 5×FAD mice, which mimic cognitive dysfunction in AD.

### 2.2. Identification and Analysis of Significantly Differentially Expressed lncRNAs (SDELs)

**Figure 3:**
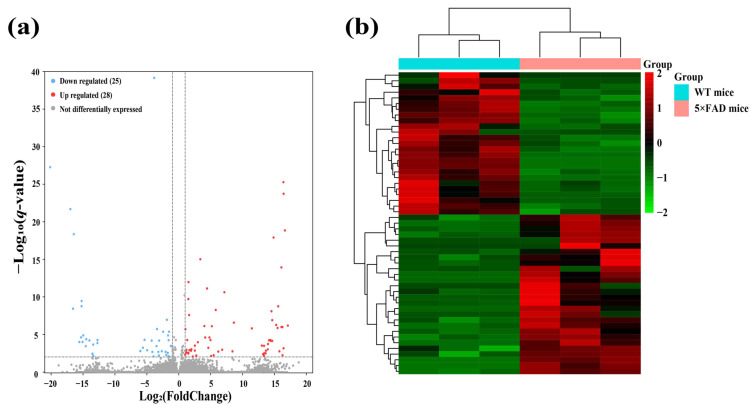

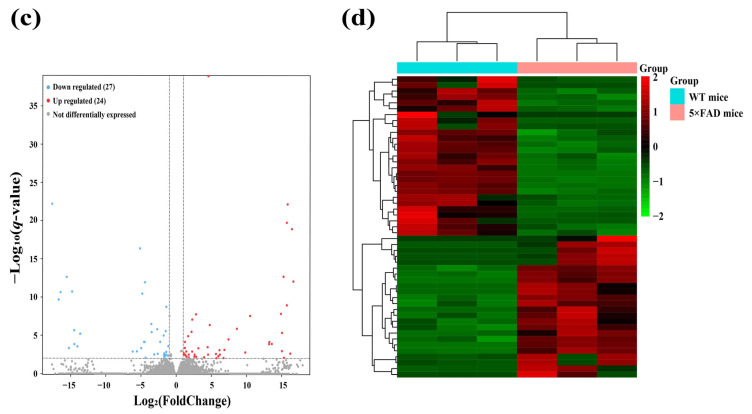
Identification of SDELs in 7-month-old 5×FAD mice compared with age-matched WT mice. Volcano plots show all lncRNAs expressed in the cerebral cortex (**a**) and hippocampus (**c**) of 5×FAD mice. The red and blue points, respectively, indicate upregulated and downregulated SDELs (|FoldChange| > 2 and *q*-value < 0.01). Hierarchical cluster analyses indicate the variation in expression levels of SDELs in the cerebral cortex (**b**) and hippocampus (**d**) of 5×FAD mice. Red and green represent relatively high and low expression levels, respectively.

To identify functional lncRNAs involved in the cortex and hippocampus, 12 cDNA libraries associated with lncRNAs were constructed, and RNA-seq was performed. The RNA-seq results obtained in these mouse brain samples showed an average of 68,085,226 total reads and an average read length of 150 bp. More detailed information on the quality assessment is presented in Table S1. We detected a total of 48,033 lncRNAs expressed in 12 samples, including 2436 novel lncRNAs and 45,597 known lncRNAs. To identify significantly altered lncRNAs, we set the screening conditions to |FoldChange| > 2 and *q*-value < 0.01. The results showed that 53 lncRNAs were significantly differentially expressed in the cerebral cortex, including 16 novel lncRNAs and 37 known lncRNAs (Table 1). A clear difference in lncRNA expression profiles in the cortex between 5×FAD and WT mice was demonstrated by volcano plots and hierarchical cluster analysis, indicating that 28 (known = 17; novel = 11) SDELs were upregulated and 25 (known = 20; novel = 5) were downregulated (Figure 3a,b). Furthermore, 51 SDELs were detected in the hippocampus of 5×FAD mice, comprising 15 novel lncRNAs and 36 known lncRNAs (Table 2). Similarly, 24 lncRNAs (known = 15; novel = 9) were upregulated and 27 (known = 21; novel = 6) were downregulated, as shown by the volcano plots and hierarchical cluster analysis (Figure 3c,d).

### 2.3. Identification of miRNAs and mRNAs Expressed in the Cerebral Cortex and Hippocampus of 5×FAD Mice

**Figure 4:**
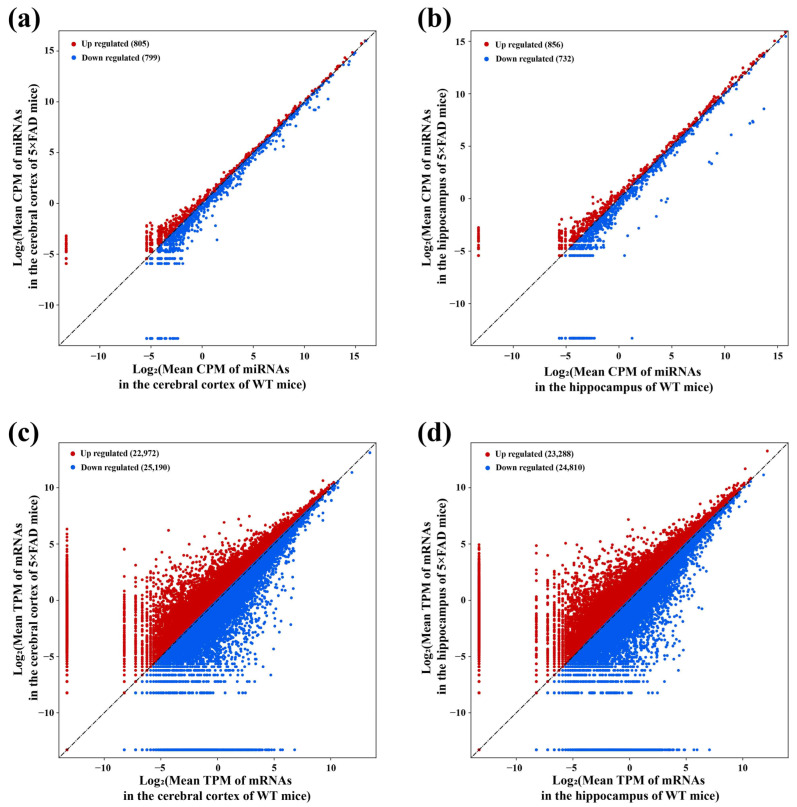
Identification of miRNAs and mRNAs with altered expression in 7-month-old 5×FAD mice compared with age-matched WT mice. The scatter plots show the number of miRNAs and mRNAs with expression changes between 5×FAD mice and WT mice. For miRNAs with altered expression in the cortex (**a**) and hippocampus (**b**), each dot represents the logarithmic value of CPM for one miRNA in both mouse groups. For mRNAs in the cortex (**c**) and hippocampus (**d**), each dot represents the logarithmic value of TPM for one mRNA in both mouse groups. Red and blue dots indicate upregulated (FoldChange > 1) and downregulated (FoldChange < −1) miRNAs and mRNAs.

The 12 cDNA libraries associated with miRNAs revealed 1604 miRNAs with expression changes in the cortex of 5×FAD mice compared with WT mice after setting the screening condition to |FoldChange| > 1 of mean count per million (CPM), comprising 805 upregulated and 799 downregulated miRNAs (Figure 4a). Similarly, 1588 miRNAs showed changes at the expression levels in the hippocampus, of which 856 were upregulated and 732 were downregulated (Figure 4b). Among them, 1489 miRNAs presented expression alteration in the cortex and hippocampus. Based on the 12 cDNA libraries associated with mRNAs, 48,162 (22,972 upregulated, 25,190 downregulated) and 48,098 (23,288 upregulated, 24,810 downregulated) mRNAs expressed in the cortex and hippocampus were revealed using the cut-off criteria of |FoldChange| > 1 for mean transcript per million (TPM) (Figure 4c,d). Among them, 45,760 mRNAs presented expression alteration in these two brain regions. These results indicated the profiles of miRNAs and mRNAs with expression alterations in the cortex and hippocampus of 5×FAD mice compared with WT mice.

### 2.4. Analysis of Key lncRNAs in the Cerebral Cortex of 5×FAD Mice

**Figure 5:**
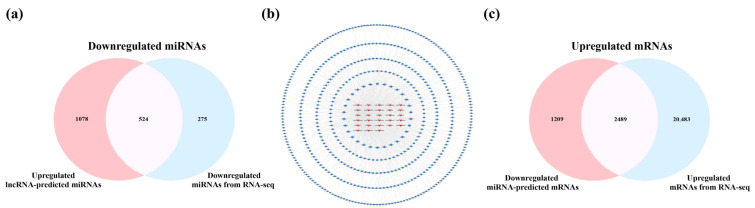

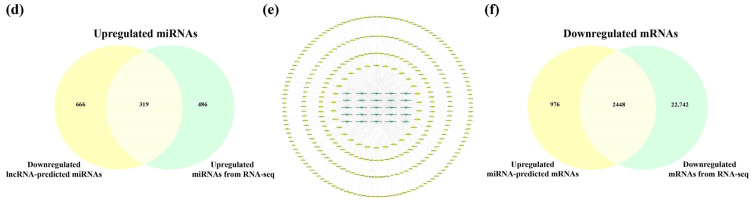
Analysis of interactions between lncRNAs, miRNAs, and mRNAs in the cortex of 5×FAD mice. The number of shared downregulated (**a**) and upregulated (**d**) miRNAs between distinct regulation of lncRNAs-predicted miRNAs and miRNAs in the cortex identified by RNA-seq. (**b**) Establishment of the upregulated lncRNA-miRNA network based on 28 upregulated SDELs and 524 miRNA targets. The V-shaped and red-orange nodes represent upregulated SDELs, while the octagonal and blue nodes represent lncRNA-targeted and downregulated miRNAs. Venn diagrams indicate the number of common upregulated (**c**) and downregulated (**f**) mRNAs between the distinct regulation of miRNA-predicted mRNAs and mRNAs identified by RNA-seq. (**e**) Establishment of the downregulated lncRNA-miRNA network by 25 downregulated SDELs and 319 miRNA targets. The V-shaped and green nodes represent downregulated SDELs, while the octagonal and yellow nodes represent targeted and upregulated miRNAs.

The SDELs with different expression trends in the cerebral cortex (upregulation = 28; downregulation = 25) and hippocampus (upregulation = 24; downregulation = 27) were separated for subsequent analysis to further explore the potential mechanism of lncRNA in the etiology of AD. We firstly analyzed the SDELs obtained from the cerebral cortex of 5×FAD mice by examining the interaction between lncRNAs, miRNAs, and mRNAs through multiple databases. Using the Starbase and LncRNASNP2 databases, we searched for the targeted miRNAs of the 17 upregulated known SDELs. The lncRNA ENSMUST00000127786 and ENSMUST00000182231 targeted miRNAs were acquired, while no miRNA targets for the other upregulated and known lncRNAs were found in these databases. Therefore, the miRNAs targeted by the sequences of the remaining 15 known lncRNAs and 11 novel lncRNAs were predicted in the miRDB database. Regarding the 20 downregulated known SDELs, a similar outcome was obtained such that only lncRNA ENSMUST00000184170 could retrieve the targeted miRNAs; the sequences of the remaining 24 downregulated SDELs were used to obtain the predicted target miRNAs in the miRDB database. After integrating all predicted miRNAs and removing duplicates, the results demonstrated that the 28 upregulated lncRNAs targeted 1602 miRNAs and that the 25 downregulated lncRNAs targeted 985 miRNAs. Using Venny 2.1.0, the overlap of the miRNAs obtained from a prediction by upregulated or downregulated SDELs and down- or upregulated miRNAs acquired from the RNA-seq results of the present study resulted in 524 and 319 overlapping miRNAs (Figure 5a,d). For the intersecting miRNAs, CytoScape 3.8.2 was applied to construct regulatory networks of upregulated and downregulated lncRNA-miRNA pairs, respectively (Figure 5b,e). The upregulated lncRNA-miRNA network contained 552 nodes and 1362 edges, while the downregulated lncRNA-miRNA network comprised 344 nodes and 453 edges. Detailed information on the lncRNA-miRNA networks is provided in Tables S2 and S3. The miRNAs involved in both networks were further screened by the Degree values to obtain key miRNAs. Among the 524 and 319 miRNAs within the two regulatory networks, 30 key miRNAs per network were obtained after the Degree value was empirically set to be greater than twice the median. Subsequently, the miRTarBase database was used to collect the downstream mRNAs of these key miRNAs. Overall, 3423 mRNAs corresponded to 30 key miRNAs with upregulation, while 3698 mRNAs were obtained by 30 key miRNAs with downregulation. To explore the connections between miRNAs and mRNAs, we compared the predicted mRNAs with the RNA-seq results obtained in these mouse brain samples. The predicted mRNAs overlapped with the 22,972 upregulated mRNAs and 25,190 downregulated mRNAs of the cortex, resulting in 2489 common upregulated mRNAs and 2448 shared downregulated mRNAs (Figure 5c,f). In this way, potentially correlated lncRNAs, miRNAs, and miRNAs were systematically identified from the cerebral cortex of 5×FAD mice.

### 2.5. Biological Role, Signaling Pathway Enrichment, and ceRNA Network Analyses of Key lncRNAs in the Cerebral Cortex of 5×FAD Mice

**Figure 6:**
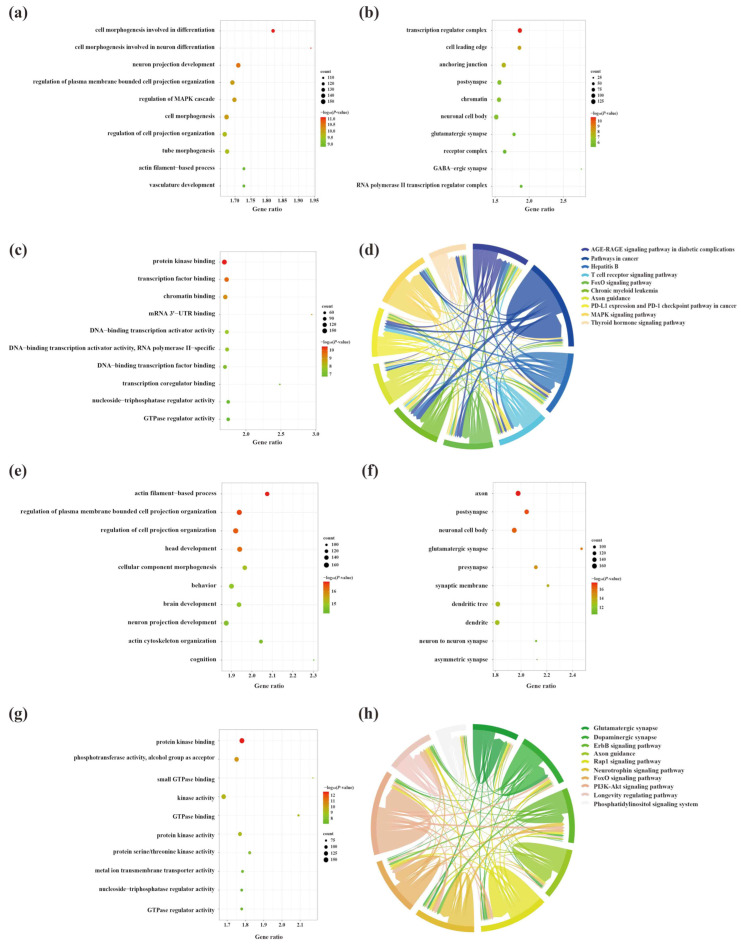
The GO function enrichment and KEGG pathway analysis results of SDELs-related mRNAs in the cerebral cortex of 5×FAD mice. GO annotation results of the top 10 BPs, CCs, and MFs related to upregulated (**a**–**c**) and downregulated (**e**–**g**) mRNAs are shown. The bubble size represents the number of targets enriched in each entry, while the color represents the enrichment significance based on the corrected *p*-value. The KEGG pathway enrichment analysis results of upregulated (**d**) and downregulated (**h**) mRNAs. Each line represents one gene; a line connecting two pathways indicates that the gene is jointly enriched in both pathways. The wider the connecting line, the greater the number of shared genes. Different pathways are presented in different colors.

To explore the molecular mechanisms underlying the involvement of SDELs in the cerebral cortex of 5×FAD mice, we performed Gene Ontology (GO) and Kyoto Encyclopedia of Genes and Genomes (KEGG) enrichment analyses of common mRNAs using the Metascape platform. The GO results of the 2489 upregulated mRNAs revealed a total of 994 GO terms, including 789 biological processes (BPs), 91 cellular compositions (CCs), and 114 molecular functions (MFs). According to the ranking by *p*-value, the top 10 terms were selected to draw a BubbleChart (Figure 6a–c). In addition, the upregulated mRNAs were enriched with 75 KEGG signaling pathways, of which the top 10 pathways are shown in a Chordal graph (Figure 6d). The main BPs involved in the upregulated lncRNA-related mRNAs included cell morphogenesis in neuron differentiation, neuron projection development, regulation of MAPK cascade, and neuron projection morphogenesis. The relevant targets mainly function in protein kinase binding, transcription factor binding, and chromatin binding at neuronal cell bodies, glutamatergic synapses, and GABA-ergic synapses. The enriched pathways were mainly associated with the MAPK signaling pathway, neurotrophin signaling pathway, and cellular senescence. The GO analyses of the downregulated mRNAs were enriched for 1175 terms, consisting of 875 BPs, 161 CCs, and 139 MFs. Moreover, 99 signaling pathways were shown to be enriched by KEGG analysis. The top 10 GO terms and KEGG pathways are shown in Figure 6e–h. The GO-enriched BPs were involved in head development, brain development, neuronal projection development, and cognition. Identification of the top 10 CCs indicated that the downregulated lncRNA-targeted mRNAs were mainly located in axons, synapses, and dendrites. The MFs of these mRNAs comprised protein kinase binding, phosphotransferase activity, alcohol group as acceptor, and small GTPase binding. Most genes were primarily concentrated in the glutamatergic synapse, dopaminergic synapse, ErbB signaling pathway, axon guidance, and neurotrophin signaling pathway.

**Figure 7:**
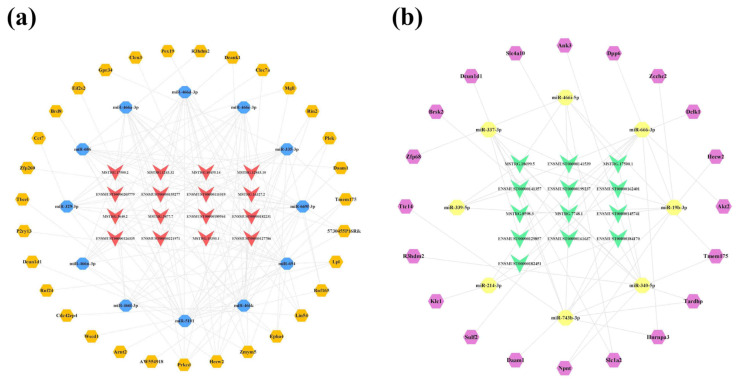
LncRNA-associated ceRNA networks in the cortex of 5×FAD mice. (**a**) Establishment of the upregulated lncRNA-miRNA-mRNA regulatory network based on 16 lncRNAs, 12 miRNAs, and 31 mRNAs. (**b**) Establishment of the downregulated lncRNA-miRNA-mRNA regulatory network based on 13 lncRNAs, 8 miRNAs, and 20 mRNAs. The lncRNAs, miRNAs, and mRNAs are indicated as V-shapes, octagons, and hexagons, respectively. The red-orange and green nodes denote upregulated and downregulated lncRNAs; yellow and blue nodes represent upregulated and downregulated miRNAs; and orange and purple nodes indicate upregulated and downregulated mRNAs.

Due to the large number of common mRNAs in the cortex, we selected key mRNAs with a |FoldChange| > 2 and *q*-value < 0.01 for network construction. As a result, 31 of 2489 mRNAs were significantly upregulated and 20 of 2448 mRNAs were significantly downregulated. Afterwards, upregulated and downregulated lncRNA-associated ceRNA networks of the cortex were constructed using CytoScape 3.8.2, respectively (Figure 7). The upregulated lncRNA-mediated ceRNA network showed that 16 lncRNAs, 12 miRNAs, and 31 mRNAs had interaction relationships, including 59 nodes and 153 edges (Figure 7a). Based on the Degree, Betweenness Centrality, and Closeness Centrality values in the network (in descending order), the novel lncRNA MSTRG.12843.10 was identified as the most active lncRNA (Degree = 12; Betweenness Centrality = 0.1226; Closeness Centrality = 0.5577) with the interactions of all miRNAs in the network. It was followed closely by MSTRG.3640.2 and ENSMUST00000127786, both targeting 11 miRNAs in the network. The downregulated lncRNA-associated ceRNA network consisted of 13 lncRNAs, 8 miRNAs, and 20 mRNAs, including 41 nodes and 53 edges (Figure 7b). In this network, the novel lncRNA MSTRG.7748.1 showed potential regulatory effects on miR-339-5p, miR-337-3p, miR-466i-5p, miR-340-5p, and miR-743b-3p. For the miRNAs, miR-743b-3p correlated with multiple lncRNAs, including ENSMUST00000145741, MSTRG.7748.1, ENSMUST00000184170, and ENSMUST00000141357. Detailed information on the lncRNA-miRNA-mRNA correspondences in the networks is provided in Tables S4 and S5.

### 2.6. Analysis of Key lncRNAs in the Hippocampus of 5×FAD Mice

**Figure 8:**
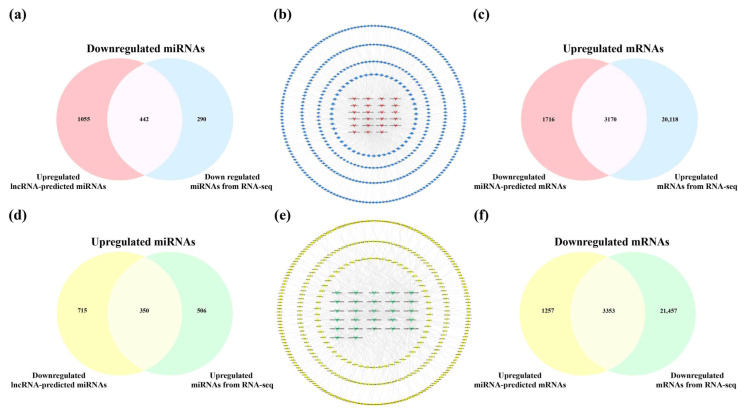
Analysis of interactions between lncRNAs, miRNAs, and mRNAs in the hippocampus of 5×FAD mice. Number of common downregulated (**a**) and upregulated (**d**) miRNAs between different regulations of lncRNAs-predicted miRNAs and miRNAs in the hippocampus from RNA-seq. (**b**) Establishment of the upregulated lncRNA-miRNA network based on 23 upregulated SDELs and 442 miRNA targets. The V-shaped and red-orange nodes represent upregulated SDELs of the hippocampus, while the octagonal and blue nodes represent lncRNA-targeted and downregulated miRNAs. Venn diagrams indicate the number of common upregulated (**c**) and downregulated (**f**) mRNAs between miRNA-predicted mRNAs and mRNAs from RNA-seq. (**e**) Establishment of the downregulated lncRNA-miRNA network by 27 downregulated SDELs and 350 miRNA targets. The V-shaped and green nodes represent downregulated SDELs of the hippocampus, while the octagonal and yellow nodes represent targeted and upregulated miRNAs.

Using the Starbase and LncRNASNP2 database, only the ENSMUST00000160679, ENSMUST00000180635, ENSMUST00000197854, and ENSMUST00000127786 targeting miRNAs could be detected in the hippocampus. Similarly, of the 21 downregulated SDELs, information for relevant miRNAs was only retrieved for ENSMUST00000184170, ENSMUST00000197200, and ENSMUST00000125930. As a result, 1497 miRNAs were predicted from the 24 upregulated SDELs, while all 27 downregulated SDELs corresponded to 1065 miRNAs. The overlapping predictions from the lncRNAs and miRNAs obtained from the RNA-seq results obtained in these mouse brain samples (upregulation = 856; downregulation = 732) were 442 upregulated and 350 downregulated miRNAs (Figure 8a,d). For the intersecting miRNAs, the upregulated lncRNA-miRNA and downregulated lncRNA-miRNA regulatory networks of the hippocampus were constructed using CytoScape 3.8.2 (Figure 8b,e). For the upregulated lncRNAs, the network contained 465 nodes and 875 edges, and the regulatory network of downregulated lncRNA-miRNA axis consisted of 377 nodes and 583 edges. A further 51 key miRNAs and 57 core miRNAs showed the closest interaction with the upregulated and downregulated SDELs, as exhibited in the innermost circle of the networks. The detailed connections in the networks are shown in Tables S6 and S7. Next, using the miRTarBase database, 4610 mRNAs corresponded to 57 upregulated miRNAs, and 4886 downstream mRNAs were retrieved by 51 downregulated miRNAs. Furthermore, 3170 common upregulated mRNAs and 3353 shared downregulated mRNAs were obtained by the 23,288 upregulated mRNAs and 24,810 downregulated mRNAs obtained from the RNA-seq results of the present study (Figure 8c,f).

### 2.7. Biological Role, Signaling Pathway Enrichment, and ceRNA Network Analyses of Key lncRNAs in the Hippocampus of 5×FAD Mice

**Figure 9:**
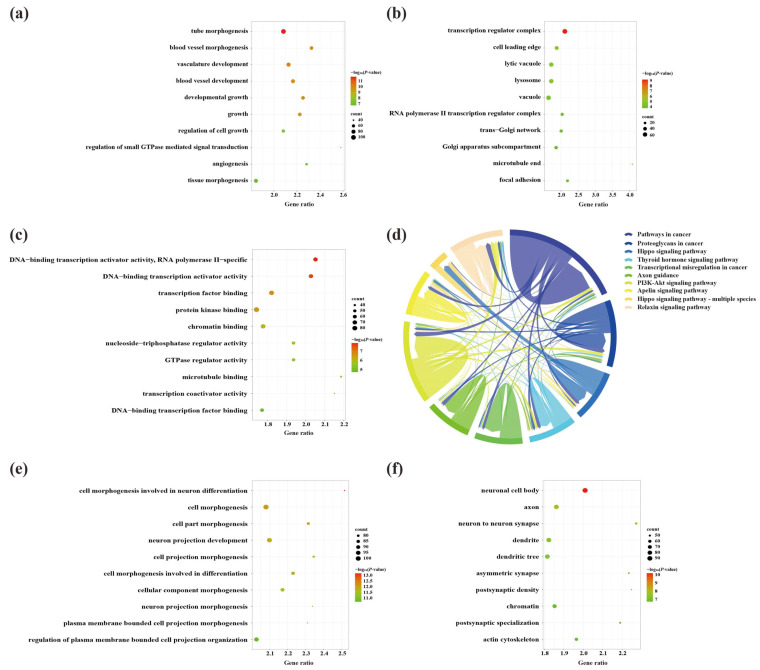

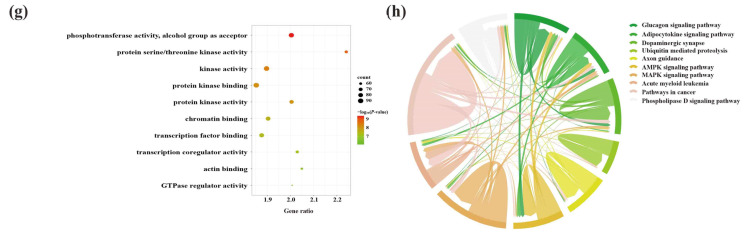
GO function enrichment and KEGG pathway analyses of SDELs-related mRNAs in the hippocampus of 5×FAD mice. GO annotation of the top 10 BPs, CCs, and MFs related to upregulated (**a**–**c**) and downregulated (**e**–**g**) mRNAs. The bubble size represents the number of targets enriched in each entry, while the color represents the enrichment significance based on the corrected *p*-value. KEGG pathway enrichment analysis of upregulated (**d**) and downregulated (**h**) mRNAs. Each line represents one gene, and a line connecting two pathways indicates that this gene is enriched in both pathways. The wider the connecting line, the greater the number of shared genes. Different pathways are presented in different colors.

A total of 748 GO terms (BP = 614; CC = 51; MF = 83) were obtained by the enrichment of 3170 upregulated mRNAs. Among the GO terms, the genes were mainly involved in BPs associated with developmental growth and neuron projection development (Figure 9a) and MFs linked to DNA-binding transcriptional activator activity, transcription factor binding at neuronal projection cytoplasm, and synapses between neurons (Figure 9b,c). Thirty-six signaling pathways were enriched by upregulated mRNAs in the hippocampus, among which the hippo signaling pathway, axon guidance, and the AMPK signaling pathway were closely linked to the etiology of AD (Figure 9d). On the other hand, the 3353 downregulated mRNAs were responsible for 709 BPs, associated with cell and neuron genesis and differentiation, including cell morphogenesis involved in neuron differentiation and neuron projection development (Figure 9e). These processes occurred in 95 CCs, including neuronal cell bodies, axons, and neuron-to-neuron synapses (Figure 9f). Similar to the results for the upregulated mRNAs, the top terms of the 123 MFs were protein kinase binding and kinase synapses (Figure 9g). Furthermore, the most enrichment of the 46 KEGG signaling pathways included many AD-associated pathways, including dopaminergic synapse, axon guidance, and AMPK signaling pathway (Figure 9h).

**Figure 10:**
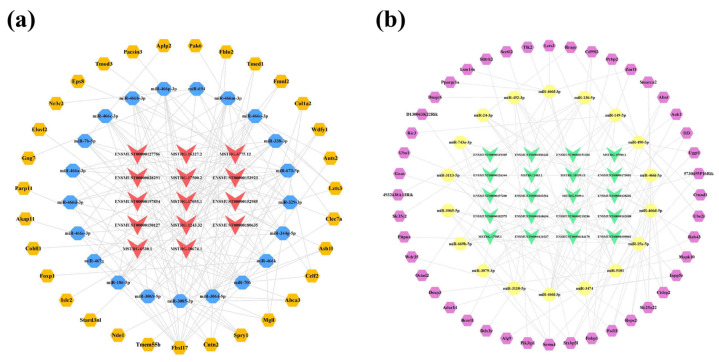
LncRNA-associated ceRNA networks in the hippocampus of 5×FAD mice. (**a**) Establishment of the upregulated lncRNA-miRNA-mRNA regulatory network based on 14 lncRNAs, 21 miRNAs, and 31 mRNAs. (**b**) Establishment of the downregulated lncRNA-miRNA-mRNA regulatory network by 20 lncRNAs, 18 miRNAs, and 44 mRNAs. The lncRNAs, miRNAs, and mRNAs are indicated as V-shapes, octagons, and hexagons, respectively. The red and green nodes denote upregulated and downregulated lncRNAs; the yellow and blue nodes represent upregulated and downregulated miRNAs; and the orange and purple nodes indicate upregulated and downregulated mRNAs.

Furthermore, 31 core mRNAs and 44 key mRNAs were differentially expressed among 3170 upregulated mRNAs and 3353 downregulated mRNAs; thereby, the upregulated lncRNA-associated network was constructed, comprising 14 lncRNAs, 21 miRNAs, and 31 mRNAs (Figure 10a). In this network, lncRNA ENSMUST00000127786 had the highest Degree and acted on 19 miRNAs, followed by three novel lncRNAs, namely MSTRG.17500.2 (Degree = 18), MSTRG.6775.12 (Degree = 16), and MSTRG.1243.32 (Degree = 15). In addition, miR-3085-3p and miR-3064-5p were associated with five identical lncRNAs (ENSMUST00000180635, ENSMUST00000127786, ENSMUST00000028291, ENSMUST00000150127, and MSTRG.17500.2) and both could bind the same seven mRNAs (Tmed1, Mgll, Fbln2, Cntn2, Abca3, Wdfy1, and Ash1l). In the downregulated lncRNA-associated network, 20 lncRNAs interacted with 18 miRNAs and 44 mRNAs downstream (Figure 10b). The novel lncRNA MSTRG.17585.1 could act on the nine miRNAs in this network. Furthermore, miR-466l-3p could interact with six lncRNAs (ENSMUST00000138202, ENSMUST00000199803, ENSMUST00000151020, MSTRG.9309.1, MSTRG.18159.11, and MSTRG.15980.1) and eight mRNAs (Wdr35, Pcbp2, Znrf1, Inpp5e, Srrm4, Slitrk2, D130043K22Rik, and Uggt1). The detailed interaction relationships of the lncRNA-miRNA-mRNA networks of the hippocampus are shown in Tables S8 and S9.

### 2.8. Analysis of Core lncRNAs in the Cerebral Cortex and Hippocampus of 5×FAD Mice

**Figure 11:**
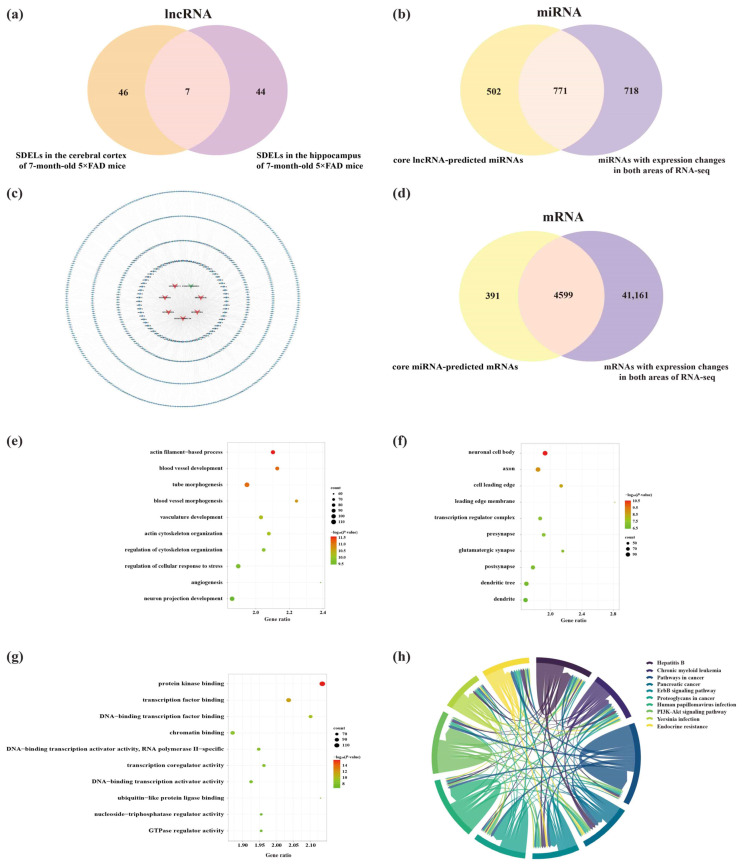
Analysis of interactions between lncRNAs, miRNAs, and mRNAs in the cerebral cortex and hippocampus of 5×FAD mice. (**a**) Number of core lncRNAs in the cortex and hippocampus obtained by RNA-seq analysis. (**b**) Number of common miRNAs between core lncRNA-predicted miRNAs and miRNAs identified in both areas by RNA-seq analysis. (**c**) Establishment of the lncRNA-miRNA regulatory network in the cortex and hippocampus of 5×FAD mice based on the seven core lncRNAs and 771 miRNA targets. The V-shaped nodes represent the core lncRNAs, while red-orange and green nodes represent upregulated and downregulated lncRNAs. The octagonal and blue nodes represent lncRNA-targeted miRNAs, respectively. (**d**) Venn diagram indicating the number of common mRNAs between the core miRNA-predicted mRNAs and mRNAs from the RNA-seq results. GO annotation of the top 10 BPs (**e**), CCs (**f**), and MFs (**g**) related to the common mRNAs. The bubble size represents the number of targets enriched in each entry, while the color represents the enrichment significance based on the corrected *p*-value. (**h**) KEGG pathway enrichment analysis of core lncRNA-associated mRNAs. Each line represents one gene, and a line connecting two pathways indicates that this gene is enriched in both pathways. The wider the connecting line, the greater the number of shared genes. Different pathways are presented in different colors.

Given the crucial roles of the cerebral cortex and hippocampus in AD [16], we identified dysregulated lncRNAs in these two areas of 7-month-old 5×FAD mice (Figure 11a). The results showed that seven lncRNAs (including two known lncRNAs and five novel lncRNAs) were significantly altered in both regions. Surprisingly, the expression trends were identical in both areas, showing one downregulated and six upregulated lncRNAs (Table 3). Subsequently, the core lncRNAs were predicted by the Starbase, LncRNASNP2, and miRDB databases. A total of 887 miRNAs were targeted by two known lncRNAs in the Starbase and LncRNASNP2 databases. Moreover, 451 miRNAs had interactions with five novel lncRNAs based on their sequencing in the miRDB database. The predictions obtained from these databases were combined and duplicates were removed, resulting in 1273 miRNAs. As previously mentioned, 1498 miRNAs were differentially altered in both brain regions in 5×FAD mice compared with WT mice. To elucidate potential relationships between lncRNAs and miRNAs, we examined the overlap of miRNAs obtained from lncRNA prediction and RNA-seq results of the present study. As a result, 771 common miRNAs were acquired (Figure 11b). Detailed information on the lncRNA-miRNA network is provided in Table S10. Consequently, the shared lncRNA-miRNA regulatory network in the cortex and hippocampus was constructed using the seven core lncRNAs and 771 miRNAs (Figure 11c). Another 4990 mRNAs were obtained by predicting the downstream mRNAs of 68 core miRNAs filtered by the Degree value of the network. Subsequently, 4599 overlapping mRNAs were identified after comparison with the 45,760 mRNAs from the RNA-seq results obtained in these mouse brain samples (Figure 11d). To investigate the etiology of AD at the transcriptional level, GO and KEGG analyses were performed for these 4599 mRNAs. The results indicated 1217 GO terms that showed enrichment, including 1015 BPs, 86 CCs, and 116 MFs. The top 10 GO-enriched domains of dysregulated mRNAs were mapped and sorted by *p*-value. The GO-enriched BP terms involved neuron projection development, regulation of neuron projection development, brain development, cognition, and regulation of neuron death (Figure 11e). The top 10 CC terms suggested that core lncRNA-associated mRNAs were located in neuronal cell bodies, axons, and glutamatergic synapses (Figure 11f). Furthermore, the MFs of related mRNAs included protein kinase binding, transcription factor binding, DNA-binding transcription factor binding, and chromatin binding (Figure 11g). Pathway enrichment analysis was used to explore key signaling pathways in the onset and progression of AD. The results revealed 101 significantly enriched pathways involving the neurotrophin signaling pathway, axon guidance, cAMP signaling pathway, hippo signaling pathway, autophagy-animal, and longevity regulating pathway (Figure 11h).

### 2.9. Construction of Potential lncRNA-Mediated Regulatory Networks and Expression Validation of RNAs

Only four mRNAs (C-type lectin domain containing 7A [Clec7a], RIKEN cDNA 5730455P16 gene [5730455P16Rik], formin-binding protein 1 [Fnbp1], and ELOVL fatty acid elongase 2 [Elovl2]) out of 4599 mRNAs were significantly differentially expressed in our RNA-seq results of the present study. Starting with the four core mRNAs from the screening results, the corresponding upstream miRNAs and lncRNAs were found to construct a lncRNA-miRNA-mRNA (L-M-T) network using CytoScape 3.8.2 (Figure S1a). The network indicated that five lncRNAs, ten miRNAs, and four mRNAs had interaction relationships. According to the Degree, Betweenness Centrality, and Closeness Centrality values of this network (in descending order), ENSMUST00000127786 was the most active lncRNA (Degree = 10; Betweenness Centrality = 0.2950; Closeness Centrality = 0.6923), interacting with all ten miRNAs in the network. Specific information about the interactions between lncRNAs, miRNAs, and mRNAs in this network is shown in Table 4.

**Figure 12:**
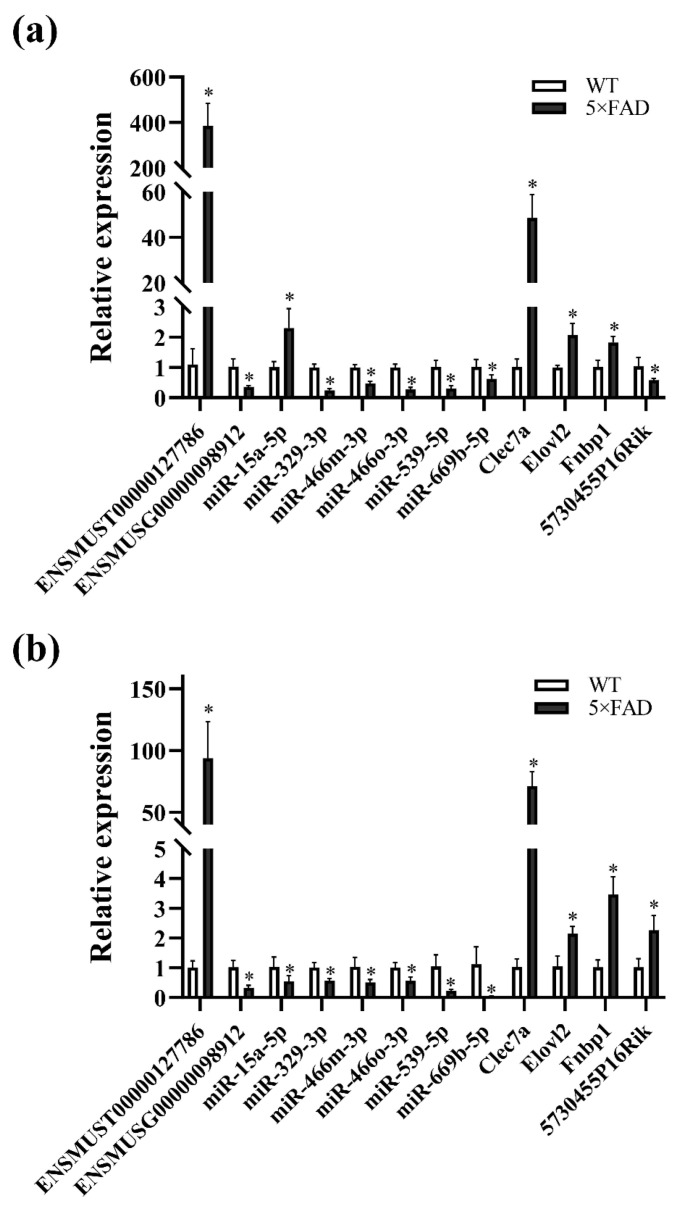
Validation of RNA expression in the L-M-T network by qPCR. (**a**) RNA expression in the cerebral cortex of 5×FAD mice compared with WT mice; (**b**) RNA expression in the hippocampus of 5×FAD mice compared with WT mice. Comparisons were analyzed using a non-parametric test. Results are presented as mean ± SD, *n* = 4. * *p* < 0.05 vs. WT mice.

**Figure 13:**
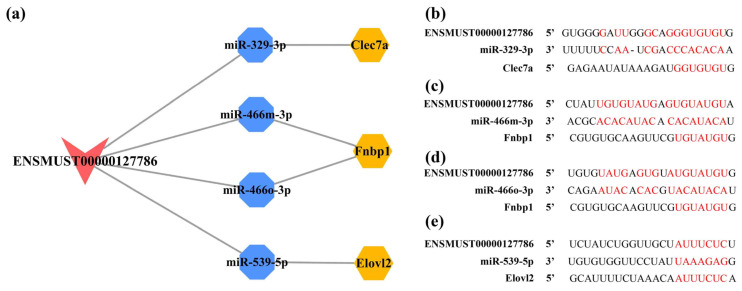
Construction of lncRNA-mediated ceRNA network and potential binding sites for RNAs in this network. (**a**) The lncRNA-mediated ceRNA regulatory network in the cortex and hippocampus of 5×FAD mice. The lncRNAs, miRNAs, and mRNAs are indicated as V shapes, octagons, and hexagons, respectively. The red node represents an upregulated lncRNA, blue nodes represent downregulated miRNAs, and orange nodes indicate upregulated mRNAs. Potential binding sites for ENSMUST00000127786 to miR-329-3p (**b**), miR-466m-3p (**c**), miR-466o-3p (**d**), and miR-539-5p (**e**) predicted by the Starbase database, as well as the potential binding sites for these miRNAs in the 3′ untranslated region (UTR) of Clec7a, Fnbp1, and Elovl2 mRNAs predicted by TargetScan database. Potential binding sites are shown in red.

It is hypothesized that ceRNAs compete for the same MREs in the regulatory network. Based on the expression trends of these RNAs in the cortex and hippocampus shown by the RNA-seq results obtained in these mouse brain samples (Table S11), it was hypothesized that ENSMUST00000127786 might regulate the levels of Clec7a, Fnbp1, and Elovl2 by sponging miR-329-3p, miR-466m-3p, miR-466o-3p, and miR-539-5p; ENSMUST00000184170 might regulate the expression of 5730455P16Rik and Fnbp1 by sponge adsorption of miR-15a-5p and miR-669b-5p (Figure S1b). miR-362-3p, miR-3057-5p, miR-466i-3p, and miR-5101 were tentatively excluded due to the inconsistent expression trends in the cortex and hippocampus from our RNA-seq results or not matching the potential ceRNA relationship (Figure S1c). Subsequently, the expression of RNAs, including ENSMUST00000127786, ENSMUST00000184170, miR-329-3p, miR-466o-3p, miR-466m-3p, miR-539-5p, Clec7a, and Elovl2, was validated to have the same expression trends in the cortex and hippocampus as the RNA-seq results using qPCR analysis (all *p* < 0.05; Figure 12a,b). However, the expression of 5730455P16Rik, Fnbp1, miR-15a-5p, and miR-669b-5p was inconsistent with the RNA-seq results (Figure 12a,b). Finally, a potential lncRNA-mediated ceRNA network was constructed by combining the RNA-seq data, expression validation results, and MRE sequencing predictions. The network suggested a possible interrelationship of ENSMUST00000127786 with miR-329-3p, miR-466m-3p, miR-466o-3p, and miR-539-5p, as well as relationships between these miRNAs and Clec7a, Fnbp1, and Elovl2 (Figure 13a). Based on the Starbase and TargetScan platforms, the potential binding sites between ENSMUST00000127786 and corresponding miRNAs and mRNAs in their potential network were obtained (Figure 13b–e).

## 3. Discussion

The global prevalence of AD is increasing each year, greatly impairing the quality of life and physical health of patients and imposing a considerable burden on families and societies. The pathology of AD is characterized by senile plaques formed by the deposition of Aβ and neuronal fiber tangles. The neuropathology of AD begins with lesions in the cerebral cortex and gradually invades the hippocampus [16]. The amyloid plaques, deposits of Aβ peptide, are defining lesions in the AD brain. Similar to APP/PS1 double transgenic mice [17], 5×FAD mice have additional mutant sites on *APP*, which increases the production of Aβ_42_ peptides. Double locus mutations on *PS1* accelerate the processes of neurodegeneration, neuronal loss, and the formation of amyloid plaques [12]. Therefore, in 5×FAD mice, intra-neuronal aggregation of Aβ appears around 1.5 months, deposition of Aβ plaques begins at 2 months, neuronal loss and spatial learning deficits appear at around 6 months, and severe cognitive dysfunction is observed at 7–8 months [18]. Thus, 5×FAD mice at 2 months may simulate the early stage of AD, while 7–8 months of age reflects the progressive stage. In our study, 7-month-old 5×FAD mice showed learning and memory dysfunction reflecting the disruption of cognition in AD. Due to the crucial role of epigenetics in the etiology of AD, it is necessary to clarify the underlying pathological mechanisms at the epigenetic level. Therefore, epigenetic profile data in the cortex and hippocampus of 5×FAD mice were analyzed by RNA-seq, with a focus on data common to both regions.

Our results revealed 53 SDELs in the cortex and 51 SDELs in the hippocampus of 7-month-old 5×FAD mice, with seven SDELs shared by both regions. Among these SDELs, 35 known lncRNAs were significantly altered only in the cortex. ENSMUST00000199237 was the lncRNA with the highest FoldChange value and is one of the transcripts of Mitotic arrest deficient 1 like 1 (Mad1l1), a susceptibility gene for bipolar disorder and schizophrenia [19], suggesting a possible role of ENSMUST00000199237 in other neuropsychiatric disorders. Of the 16 novel lncRNAs, MSTRG.14838.1 and MSTRG.8598.3 were the most upregulated and downregulated in the cortex. Numerous novel lncRNAs, such as MSTRG.8598.3, MSTRG.7748.1, and MSTRG.14838.1, showed better FoldChange values than known lncRNAs. Moreover, KEGG analysis indicated that both upregulated and downregulated lncRNAs were involved in the glutamatergic synapse, cell senescence, and the neurotrophin signaling pathway and enriched in biological processes associated with the regulation of synapse structure or activity, cerebral cortex cell migration, and cerebral cortex development, revealing potential mechanisms for the roles of these novel lncRNAs in AD. In the upregulated lncRNA-mediated ceRNA network, MSTRG.12843.10 had possible interactions with all miRNAs. For the downregulated lncRNA-mediated ceRNA network, MSTRG.7748.1 showed significant downregulation and potentially targeted the majority of miRNAs to indirectly affect the expression of several mRNAs, suggesting that MSTRG.7748.1 is worthy of further investigation in the etiology of AD.

Among the 51 SDELs in the hippocampus, the genes corresponding to many of the 36 known SDELs are of interest in the neurological field, such as ENSMUST00000182575, which possessed the largest FoldChange and showed significant downregulation in the RNA-seq results. In addition, its gene ontology, autism susceptibility candidate 2 (Auts2), is reported to be highly expressed in both the cortex and hippocampus of mice and is essential for the development of the cerebral cortex [20], suggesting that abnormal ENSMUST00000182575 expression in the hippocampus may be related to impaired cognitive abilities in 5×FAD mice. Notably, 24 upregulated and 27 downregulated lncRNAs were engaged in the KEGG pathway of axon guidance and biological processes associated with cognition, learning or memory, and neuron migration. Several novel lncRNAs, such as MSTRG.6775.12 and MSTRG.9309.1, had higher variation in the hippocampus, and KEGG and GO analyses indicated their biological roles in dopaminergic synapse, axon guidance, and the AMPK signaling pathway, revealing potential effects for future studies. Regarding the upregulated lncRNA-associated ceRNA network, MSTRG.6775.12 had the highest FoldChange value in the hippocampus and possible ceRNA actions with 16 miRNAs, thus regulating the expression of 19 downstream mRNAs. Furthermore, ENSMUST00000150127 showed a potential regulatory relationship with miR-3064-5p and miR-3085-3p, resulting in the altered expression of several mRNAs, such as Wdfy1 [21], Mgll [22], and Cntn2 [23], related to neurodevelopmental functions and neurodegenerative mechanisms. In the downregulated lncRNA-associated ceRNA network, MSTRG.17585.1 potentially targeted several miRNAs, among which miR-3065-5p has been shown to be specifically expressed in the plasma of AD patients, differentiating AD from other cognitive disorders [24]. Surprisingly, ENSMUST00000182575 also had a potential regulatory function on miR-3065-5p and affected the expression of Stxbp5l and Srrm4. A previous study reported high expression of Stxbp5l in the hippocampus and associations with the processes of brain development and normal neurotransmission [25].

The cerebral cortex and hippocampus reflect independent and parallel memory systems [26]. In terms of learning and memory functions, the cortex plays a larger role in short-term memory while the hippocampus is crucial for learning contextual information and long-term memory functions [27]. Therefore, analyses of lncRNAs specifically expressed in both areas are helpful for exploring the potential action of lncRNAs on learning and memory impairment in AD. Our results identified seven common SDELs, including two known lncRNAs (ENSMUST00000127786 and ENSMUSG00000098912) and five novel lncRNAs. ENSMUST00000127786 was significantly upregulated in both areas and the FoldChange values were relatively high compared to other known lncRNAs. ENSMUST00000127786 is a spliced transcript variant of X inactive-specific transcript (Xist) that has been reported to be abnormally expressed in AD [28]. A recent study revealed that silencing Xist attenuated AD-associated BACE1 alterations via the miR-124/BACE1 signaling pathway [29], which encourages speculation that Xist is a potential target for the treatment of AD. Xist is located on the X chromosome, producing a 15- to 17-kb lncRNA that accumulates over the chromosome from which it is transcribed, recruiting factors/complexes that modify the underlying chromatin environment and repress X-linked gene expression [30]. Studies have shown that Xist is expressed in undifferentiated embryonic stem cells of male and female mice [31]. Although Xist is closely related to sex, the influence of sex factors on the transcription of Xist in AD pathology is still on the exploration road. ENSMUSG00000098912, a significantly downregulated lncRNA, had the highest Degree value (compared to known lncRNAs) in the downregulated lncRNA-mediated ceRNA network and interacted with a wide range of miRNAs, including miR-214-3p, miR-24-3p, miR-743b-3p, etc., and shows multiple correlations with pathologies of autophagy and apoptosis in AD [32,33]. The GO and KEGG analysis results provide a basic understanding of the biological characteristics of the novel lncRNAs, suggesting they act as ceRNAs through the neurotrophin signaling pathway, axon guidance, and hippo signaling pathway. Thus, lncRNAs with significant changes in the cortex and hippocampus might have biological actions related to AD via ceRNA mechanisms.

To explore potential relationships between differentially expressed genes, we initially constructed a shared L-M-T network by combining predictions from databases and RNA-seq results. Thus, we got a network of five lncRNAs, six miRNAs, and four mRNAs. Expression verification of the RNAs in the L-M-T network by qPCR analysis was performed and compared with the RNA-seq results, thus tentatively generating a potential lncRNA-mediated ceRNA network that might be associated with AD. This network conceptualized that ENSMUST00000127786 might regulate the expression of Clec7a, Fnbp1, and Elovl2 by sponging miR-329-3p, miR-466m-3p, miR-466o-3p, and miR-539-5p. Thus, we might offer a novel view of the interplay between lncRNA, miRNAs, and mRNAs. As reported, Xist directly interacts with miR-329-3p, manifesting that Xist silencing-induced cell proliferation and apoptosis were abolished by miR-329-3p inhibitor [34]. Another predicted ceRNA regulation was a possible relationship between ENSMUST00000127786 and miR-539-5p due to the report that Xist directly inhibits the miR-539-5p expression and miR-539-5p inhibitor partially reverses the effect of Xist depletion on the proliferation and migration of oxidized low-density lipoprotein-stimulated vascular smooth muscle cells [35]. In the predicted network, miR-466m-3p and miR-466o-3p, which might have a correlation with ENSMUST00000127786, showed expression changes in AD for the first time. As for the mRNAs in the L-M-T network, *Clec7a* is an Aβ plaque-associated marker in microglia, displaying a significantly increased expression in 5×FAD mice [36]. Moreover, higher levels of DNA methylation of *Elovl2* have been reported in the early stages of AD, indicating it could be used as an epigenetic marker of early AD [37]. Genetic variants in *Elovl2* are known to increase the risk of developing AD [38]. Although *Fnbp1* is poorly studied in AD, it has been reported as a risk gene for ALS [39]. The potential characteristics of these RNAs in AD and the predicted ceRNA mechanisms of our findings are just the beginning; further investigation is necessary to understand the regulation of this network in AD.

The present study has certain limitations. First, our study is a pilot experiment; more deep-going validation experiments should be performed to determine the lncRNA-miRNA and miRNA-mRNA interactions within the potential lncRNA-mediated ceRNA network and further understand their biological roles in AD. Second, the gender heterogeneity of the small samples in 5×FAD mice may limit the reliability of our analyses of these RNA-seq results; therefore, our future studies should include a more significant number of AD animal models, gender factors, and the dynamic changes in the disease procession. Third, our analyses for lncRNA from the RNA-seq data are not comprehensive. Only lncRNAs having a poly-A tail were used to build the libraries; accordingly, only such lncRNAs were analyzed.

In summary, our study was the first to identify SDELs in the cerebral cortex and hippocampus of 5×FAD mice via RNA-seq technology and to construct a potential lncRNA-mediated ceRNA network of the pathogenesis of AD with qPCR transcription level validation. These findings may provide new insights into understanding the pathological mechanisms of lncRNA, as well as potential therapeutic targets for drug discovery in AD.

## 4. Materials and Methods

### 4.1. Animals

Seven-month-old 5×FAD mice on the C57BL/6J genetic background expressing *APP* containing the Swedish (K595N/M596L), Florida (I716V), and London (V717I) mutations and *PS1* with mutations on M146L and L286V were obtained from the Academy of Military Medical Sciences in Beijing, China. The age-matched, nontransgenic littermates were used as WT mice. Each group included two females and two males (*n* = 4 per group). All procedures were approved by the ethical committee of the Institute of Medicinal Biotechnology (No. IMB-20210602D101).

### 4.2. Morris Water Maze (MWM) Test

The MWM test was used to examine the spatial learning and memory activities of 5×FAD and WT mice. The test procedure was the same as described in our previous studies [40,41]. Briefly, the MWM test is divided into two parts: a spatial learning trial and a probe trial. The spatial learning trial was conducted four times per day for five consecutive days. The escape latency (i.e., time to reach the platform in seconds) and swimming speed (mm/s) were recorded during the spatial learning trial, followed by a probe test 24 h and 48 h after the last training session. When the platform was removed, the mice were placed in the water in the furthest quadrant from the original platform location and allowed to swim for 60 s. The duration spent in the target quadrant, number of crossings in the platform, and action tracks were recorded. Data were analyzed by repeated-measures ANOVA and one-way ANOVA using SPSS software, version 25.0 (IBM, Armonk, NY, USA).

### 4.3. Sample Preparation and RNA Extraction

Following behavioral testing, all mice (eight in total) were sacrificed the day after MWM test. During this process, we took the tissue samples of the cerebral cortex and hippocampal from each mouse. Subsequently, the RNA-seq analysis was performed by using the tissue samples from three 5×FAD mice and three WT mice (two females and one male per group). Each of the mouse tissue samples was sequenced as a separate and independent sample with no replicates. Total RNA was isolated from the cerebral cortex and hippocampus using TRIzol reagent (Invitrogen, Carlsbad, CA, USA) according to the manufacturer’s instructions. The total RNA concentration was assessed using a Spark 20M multimode microplate reader (Tecan Group, Mannedorf, Switzerland). The integrity of RNA was evaluated using 1% agarose gel electrophoresis.

### 4.4. lncRNA Library Construction and Sequencing

To construct the lncRNA library, sequencing of paired ends mode was used. rRNA was removed from the total RNA of all tissue samples using the Ribo-off rRNA Depletion kit (Vazyme Biotech, Nanjing, China) based on the enzymatic reaction between the DNA probe and RNaseH. The VAHTS^TM^ Stranded mRNA-seq Library Prep Kit by Illumina^®^ (Vazyme Biotech, Nanjing, China) was employed for fragmentation, cDNA synthesis, cDNA fragment modification, magnetic bead purification and fragmentation sorting, and library amplification according to the manufacturer’s recommendations. After quality control and purification, the lncRNA library was obtained to meet the requirements of Illumina platform sequencing. During the transcript splicing process, the reference-based transcriptome analysis method was used based on aligning the sequenced reads to a reference genome, followed by assembling overlapping alignments into transcripts. Genomic localization results of transcripts for each sample were assembled using StringTie software. The assembly results of all samples were then merged, during which process the transcripts with low expression (fragments per kilobase of exon model per million mapped reads [FPKM] < 1) were discarded. For the obtained transcripts with polyA tails, we then constructed lncRNA libraries based on the screening criteria, including the number of transcript exons (transcripts with the number of exons ≥ 2), transcript length (>200 bp), known annotation, and whether the transcripts had protein-coding potential (using coding potential analysis tools of Coding Potential Calculator 2 [CPC2], Coding-Non-Coding Index [CNCI], Pfam, and predictor of lncRNAs and mRNAs based on an improved *k*-mer scheme [PLEK]).

### 4.5. miRNA Library Construction and Sequencing

Based on the structural characteristics of small RNA with a 3′ hydroxyl group and 5’′ phosphate group, a miRNA library was constructed by sequencing of single-end mode after the processes of 3′ adapter-joining, reverse transcription, 5′ adapter-joining, cDNA single-strand synthesis, and library amplification using the T4 RNA Ligase 1 and 2 (New England Biolabs, MA, USA), and M-MuLV Reverse Transcriptase (Sangon Biotech, Shanghai, China) kits according to the enzyme-catalyzed reaction. To meet the requirements of Illumina platform sequencing, miRNAs were only obtained after quality control and purification.

### 4.6. mRNA Library Construction and Sequencing

Total RNA Extractor TRIzol regeant (Sangon Biotech, Shanghai, China) and the Qubit2.0 RNA Assay Kit (Life, Carlsbad, CA, USA) were used for tissue fragmentation, nucleic acid extraction, and purification of mouse tissues by paired-end mode sequencing. We ensured that the total RNA samples met the quality requirements for mRNA library construction.

### 4.7. Differential Expression and Bioinformatics Analyses

Differential expression and bioinformatics analyses were performed to identify lncRNAs, miRNAs, and mRNAs with differential expression between 5×FAD and WT mice using the “edgeR” packages in R [42]. To make the lncRNA and mRNA expression levels comparable across samples and experiments, we represented the expression levels of lncRNAs and mRNAs in units of TPM, a superior measure when considering the sequencing depth and gene length along with the effect of samples on the counts of reads [43]. With the miRDeep2 software, we normalized the number of counts obtained from the quantification of miRNAs to CPM values and thus calculated the FoldChange values of expressed miRNAs between the two mouse groups. Expression differences were evaluated using Student’s *t*-test. A |FoldChange| > 2.0 and a *q*-value < 0.01 were both required to distinguish lncRNA, miRNAs, and mRNAs that were significantly dysregulated in the cerebral cortex and hippocampus of 7-month-old 5×FAD mice compared to age-matched WT mice. Subsequently, the TPM values of SDELs in each sample were further transformed by z-score normalization to obtain the hierarchical cluster analysis using the bioinformatics platform (http://www.bioinformatics.com.cn, accessed on 5 April 2022). To establish the relationship between lncRNAs, miRNAs, and mRNAs, we used the Starbase database (https://starbase.sysu.edu.cn/, accessed on 26 January 2022), LncRNASNP2 database (http://bioinfo.life.hust.edu.cn/lncRNASNP#!/, assessed on 28 January 2022), and miRDB database (http://mirdb.org/, assessed on 8 February 2022) to predict miRNAs interacting with lncRNAs. The Starbase database contains gene expression data from 10,882 RNA-seq and 10546 miRNA-seq, providing a platform for survival and differential expression analysis of miRNAs, lncRNAs, and mRNAs. The LncRNASNP2 database is a comprehensive resource for lncRNAs of different species, as well as the binding relationships between lncRNAs and miRNAs. miRDB is a database for miRNA target prediction. All targets in miRDB are predicted by a bioinformatics tool, MirTarget, which is developed by analyzing thousands of miRNA-target interactions via high-throughput sequencing experiments. Due to the function of providing comprehensive information about experimentally validated miRNA-target interactions, the MiRTarBase database (http://mirtarbase.mbc.nctu.edu.tw/, assessed on 3 March 2022) was used to predict the downstream mRNAs of miRNAs.

### 4.8. GO Function Enrichment and KEGG Pathway Analyses

GO and KEGG analyses of lncRNA-associated mRNAs were performed using the Metascape (https://metascape.org/, assessed on 10 March 2022) platform. A *p*-value < 0.01 indicated significant enrichment. Metascape platform integrates multiple authoritative data sources to enable the annotation of large quantities of genes in pathway enrichment and biological processes.

### 4.9. Construction of the lncRNA-Mediated ceRNA Network and Detection of RNA Expression by qPCR

Possible correlations between lncRNAs and mRNAs were evaluated according to the condition of inclusion of the same miRNA target. Cytoscape software was utilized for visualization and to construct a diagram of the L-M-T network. All the tissues taken on the second day of the MWM test were used to detect differentially expressed RNAs in the predicted networks using qPCR assays. Total tissue RNA was extracted using TRIzol reagent (Invitrogen, Carlsbad, CA, USA) according to the manufacturer’s guidelines. The primers used in this study are shown in Table 5. miRNAs were evaluated using the miRNA 1st Strand cDNA Synthesis Kit and miRNA Universal SYBR qPCR Master Mix (Vazyme Biotech, Nanjing, China) following the manufacturers’ instructions. The mRNAs and lncRNAs were reverse transcribed to complementary DNA (cDNA) using a HiScript III 1st Strand cDNA synthesis kit (Vazyme Biotech, Nanjing, China), after which qPCR was performed using ChamQ Universal SYBR qPCR Master Mix (Vazyme Biotech, Nanjing, China). The glyceraldehyde-3-phosphate dehydrogenase gene (GAPDH) and U6 were used as controls. Relative gene expression levels were calculated using the 2^−ΔΔCT^ method and analyzed by a non-parametric test using SPSS software, version 25.0 (IBM, Armonk, NY, USA). Combined with the validation results by qPCR, the lncRNA-mediated ceRNA network was constructed for 7-month-old 5×FAD mice. In addition, using Starbase and TargetScan (https://www.targetscan.org/mmu_71/, assessed on 11 May 2022) databases, we identified the locations of potential MREs for lncRNA-miRNA-mRNA interactions in the network. We used the Starbase database to retrieve the binding sites of lncRNA to miRNAs, and the TargetScan database to obtain the binding sites of miRNAs to mRNAs.

## Figures and Tables

**Table 1:** Basic information of upregulated and downregulated SDELs in the cerebral cortex of 7-month-old 5×FAD mice.

Regulation	lncRNA ID	Mean Transcript per Million (TPM)		log_2_ Fold Change	*p*-Value	*q*-Value
		5×FAD Mice	WT Mice			
Upregulated SDELs	ENSMUST00000141677	19.5700	6.9000	1.50	2.55 × 10^−5^	3.46 *×* 10^−3^
	ENSMUST00000131052	5.2167	0.2933	4.15	7.73 × 10^−8^	2.58 × 10^−5^
	ENSMUST00000189564	7.1500	1.5533	2.20	8.06 × 10^−5^	8.92 × 10^−3^
	ENSMUST00000155277	9.8033	4.9000	1.00	3.88 × 10^−5^	4.94 × 10^−3^
	ENSMUST00000123670	6.5933	1.7300	1.93	9.33 × 10^−6^	1.49 × 10^−3^
	ENSMUST00000150644	5.6833	0.0167	8.41	1.18 × 10^−5^	1.81 × 10^−3^
	ENSMUST00000156420	9.3233	0.3333	4.81	7.75 × 10^−8^	2.58 × 10^−5^
	ENSMUST00000221971	17.5367	8.4833	1.05	4.92 × 10^−9^	2.02 × 10^−6^
	ENSMUST00000205311	7.7400	2.6833	1.53	2.00 × 10^−5^	2.83 × 10^−3^
	ENSMUST00000126335	8.0900	3.2067	1.34	1.91 × 10^−7^	5.62 × 10^−5^
	ENSMUST00000127786	131.1167	0.0467	11.46	3.74 × 10^−9^	1.57 × 10^−6^
	ENSMUST00000182231	5.6033	0.0001	15.77	1.17 × 10^−5^	1.80 × 10^−3^
	ENSMUST00000111019	11.4500	1.9933	2.52	1.42 × 10^−6^	3.14 × 10^−4^
	ENSMUST00000160318	12.4067	1.5533	3.00	4.29 × 10^−7^	1.10 × 10^−4^
	ENSMUST00000148373	9.0667	2.2800	1.99	5.25 × 10^−6^	9.48 × 10^−4^
	ENSMUST00000205779	11.2933	3.9233	1.53	1.03 × 10^−15^	1.08 × 10^−12^
	ENSMUST00000145025	12.8433	1.2333	3.38	6.51 × 10^−19^	9.91 × 10^−16^
	MSTRG.10455.14	17.0067	7.5200	1.18	1.62 × 10^−5^	2.38 × 10^−3^
	MSTRG.11359.1	6.9667	0.0001	16.09	2.38 × 10^−9^	1.04 × 10^−6^
	MSTRG.15393.1	6.9567	0.0001	16.09	9.26 × 10^−18^	1.19 × 10^−14^
	MSTRG.17500.2	8.6633	0.0001	16.40	1.44 × 10^−29^	5.35 × 10^−26^
	MSTRG.12843.10	7.8933	0.0001	16.27	4.89 × 10^−5^	5.94 × 10^−3^
	MSTRG.16327.2	8.7600	0.0001	16.42	3.75 × 10^−6^	7.06 × 10^−4^
	MSTRG.3640.2	8.8033	0.0001	16.43	5.55 × 10^−28^	1.88 × 10^−24^
	MSTRG.3877.7	8.7900	0.5433	4.02	1.72 × 10^−9^	7.76 × 10^−7^
	MSTRG.14838.1	14.0300	0.0001	17.10	1.45 × 10^−9^	6.67 × 10^−7^
	MSTRG.1243.32	10.1600	0.0001	16.63	6.51 × 10^−23^	1.43 × 10^−19^
	MSTRG.5009.1	7.7933	0.0001	16.25	2.35 × 10^−9^	1.03 × 10^−6^
Downregulated SDELs	ENSMUST00000145774	0.6400	5.8500	−3.19	1.41 × 10^−5^	2.11 × 10^−3^
	ENSMUST00000132392	8.0467	23.1867	−1.53	1.13 × 10^−8^	4.37 × 10^−6^
	ENSMUST00000182451	3.9300	12.9333	−1.72	2.04 × 10^−5^	2.87 × 10^−3^
	ENSMUST00000161415	1.6833	9.1633	−2.44	1.14 × 10^−8^	4.37 × 10^−6^
	ENSMUST00000161637	0.4733	19.3467	−5.35	1.72 × 10^−7^	5.24 × 10^−5^
	ENSMUST00000124691	0.3933	6.9067	−4.13	1.45 × 10^−5^	2.15 × 10^−3^
	ENSMUST00000141539	3.9967	12.0300	−1.59	2.02 × 10^−7^	5.84 × 10^−5^
	ENSMUST00000162401	1.7367	6.2900	−1.86	2.21 × 10^−10^	1.14 × 10^−7^
	ENSMUST00000139277	31.3367	121.4033	−1.95	4.41 × 10^−5^	5.46 × 10^−3^
	ENSMUST00000129857	1.1600	21.8867	−4.24	2.39 × 10^−7^	6.65 × 10^−5^
	ENSMUST00000184170	0.0001	9.6800	−16.56	6.00 × 10^−12^	3.79 × 10^−9^
	ENSMUST00000209301	4.8000	10.6767	−1.15	1.75 × 10^−6^	3.74 × 10^−4^
	ENSMUST00000199237	0.1767	8.6533	−5.61	3.10 × 10^−6^	5.98 × 10^−4^
	ENSMUST00000137980	8.0300	18.9333	−1.24	1.63 × 10^−6^	3.51 × 10^−4^
	ENSMUST00000141357	2.9133	8.0067	−1.46	9.04 × 10^−5^	9.80 × 10^−3^
	ENSMUST00000210394	1.2167	13.6133	−3.48	1.68 × 10^−6^	3.61 × 10^−4^
	ENSMUST00000146588	0.9167	9.7400	−3.41	4.69 × 10^−9^	1.93 × 10^−6^
	ENSMUST00000144628	4.8300	30.4733	−2.66	1.13 × 10^−5^	1.76 × 10^−3^
	ENSMUST00000145741	0.5200	16.0600	−4.95	7.81 × 10^−6^	1.29 × 10^−3^
	ENSMUST00000221246	0.3400	5.2700	−3.95	7.80 × 10^−5^	8.68 × 10^−3^
	MSTRG.17500.1	0.6133	8.8667	−3.85	1.23 × 10^−43^	7.04 × 10^−40^
	MSTRG.18699.11	0.0001	8.9733	−16.45	2.11 × 10^−22^	4.50 × 10^−19^
	MSTRG.7748.1	0.0001	12.9100	−16.98	7.52 × 10^−26^	2.08 × 10^−22^
	MSTRG.18699.5	7.7767	32.3900	−2.06	1.40 × 10^−5^	2.09 × 10^−3^
	MSTRG.8598.3	0.0001	116.1733	−20.15	1.39 × 10^−31^	5.44 × 10^−28^

**Table 2:** Basic information of upregulated and downregulated SDELs in the hippocampus of 7-month-old 5×FAD mice.

Regulation	lncRNA ID	Mean TPM		log_2_ Fold Change	*p*-Value	*q*-Value
		5×FAD Mice	WT Mice			
Upregulated SDELs	ENSMUST00000144016	100.2933	37.1633	1.43	7.69 × 10^−6^	1.30 × 10^−3^
	ENSMUST00000141374	11.2500	4.4133	1.35	3.44 × 10^−5^	4.47 × 10^−3^
	ENSMUST00000028291	5.9100	0.2167	4.77	1.06 × 10^−9^	4.41 × 10^−7^
	ENSMUST00000154192	8.7400	1.7967	2.28	1.88 × 10^−10^	8.49 × 10^−8^
	ENSMUST00000160679	6.0767	1.8800	1.69	4.36 × 10^−8^	1.27 × 10^−5^
	ENSMUST00000153923	8.1333	2.9133	1.48	5.19 × 10^−5^	6.30 × 10^−3^
	ENSMUST00000218620	16.3267	0.7067	4.53	2.36 × 10^−5^	3.26 × 10^−3^
	ENSMUST00000211218	13.5300	5.7400	1.24	2.18 × 10^−6^	4.44 × 10^−4^
	ENSMUST00000180635	10.6967	4.5000	1.25	2.93 × 10^−7^	7.31 × 10^−5^
	ENSMUST00000150127	9.7400	0.4167	4.55	8.10 × 10^−44^	1.21 × 10^−39^
	ENSMUST00000127786	124.8767	0.0867	10.49	5.72× 10^−11^	2.86 × 10^−8^
	ENSMUST00000152985	6.0333	0.0067	9.82	1.07 × 10^−5^	1.72 × 10^−3^
	ENSMUST00000145174	19.7200	1.2567	3.97	6.20 × 10^−5^	7.31 × 10^−3^
	ENSMUST00000132577	6.4433	2.6667	1.27	2.52 × 10^−5^	3.45 × 10^−3^
	ENSMUST00000197854	27.5900	13.1100	1.07	2.18 × 10^−5^	3.08 × 10^−3^
	MSTRG.11359.1	11.7967	0.0300	8.62	3.67 × 10^−9^	1.36 × 10^−6^
	MSTRG.17500.2	5.7800	0.0001	15.82	1.77 × 10^−26^	7.06 × 10^−23^
	MSTRG.16327.2	7.6200	0.0001	16.22	1.56 × 10^−5^	2.36 × 10^−3^
	MSTRG.6530.1	19.1900	0.1800	6.74	8.09 × 10^−5^	9.10 × 10^−3^
	MSTRG.6775.12	10.2333	0.0001	16.64	7.89 × 10^−16^	8.71 × 10^−13^
	MSTRG.10674.1	5.3633	0.0001	15.71	6.04 × 10^−24^	1.80 × 10^−20^
	MSTRG.17055.1	5.4067	0.0001	15.72	1.75 × 10^−12^	1.14 × 10^−9^
	MSTRG.14838.1	13.2767	0.0767	7.44	1.23 × 10^−7^	3.31 × 10^−5^
	MSTRG.1243.32	8.8267	0.0001	16.43	4.48 × 10^−23^	1.22 × 10^−19^
Downregulated SDELs	ENSMUST00000125930	5.2800	14.2633	−1.43	2.43 × 10^−5^	3.35 × 10^−3^
	ENSMUST00000182575	0.0001	20.5667	−17.65	1.47 × 10^−26^	6.25 × 10^−23^
	ENSMUST00000119305	2.1933	19.1767	−3.13	2.26 × 10^−5^	3.18 × 10^−3^
	ENSMUST00000218432	11.9700	31.6733	−1.40	3.01 × 10^−12^	1.85 × 10^−9^
	ENSMUST00000138202	0.2633	5.7567	−4.45	1.06 × 10^−15^	1.13 × 10^−12^
	ENSMUST00000182642	2.0900	10.1333	−2.28	2.83 × 10^−7^	7.14 × 10^−5^
	ENSMUST00000162088	0.0001	10.6933	−16.71	2.94 × 10^−13^	2.12 × 10^−10^
	ENSMUST00000184170	2.6967	8.6433	−1.68	1.49 × 10^−5^	2.26 × 10^−3^
	ENSMUST00000203561	2.6567	7.0467	−1.41	8.76 × 10^−6^	1.45 × 10^−3^
	ENSMUST00000126582	1.2467	8.1333	−2.71	4.24 × 10^−9^	1.55 × 10^−6^
	ENSMUST00000126344	2.8900	6.1167	−1.08	3.37 × 10^−5^	4.40 × 10^−3^
	ENSMUST00000168634	5.0733	14.7667	−1.54	3.31 × 10^−5^	4.34 × 10^−3^
	ENSMUST00000151020	0.4867	5.4433	−3.48	1.13 × 10^−8^	3.83 × 10^−6^
	ENSMUST00000134954	2.4700	7.9333	−1.68	6.42 × 10^−5^	7.52 × 10^−3^
	ENSMUST00000218286	1.0900	6.7000	−2.62	1.75 × 10^−5^	2.60 × 10^−3^
	ENSMUST00000197200	3.1900	7.1833	−1.17	3.42 × 10^−5^	4.45 × 10^−3^
	ENSMUST00000185523	3.6467	7.9267	−1.12	1.16 × 10^−6^	2.63 × 10^−4^
	ENSMUST00000131037	1.3800	48.6333	−5.14	2.01 × 10^−20^	4.30 × 10^−17^
	ENSMUST00000173081	0.2433	6.9100	−4.83	4.30 × 10^−14^	3.52 × 10^−11^
	ENSMUST00000199803	0.1133	5.4200	−5.58	7.35 × 10^−6^	1.25 × 10^−3^
	ENSMUST00000220428	10.6567	30.2200	−1.50	2.87 × 10^−5^	3.85 × 10^−3^
	MSTRG.2483.2	0.2633	8.2800	−4.97	2.44 × 10^−6^	4.84 × 10^−4^
	MSTRG.16159.9	2.2767	26.3967	−3.54	7.92 × 10^−10^	3.31× 10^−7^
	MSTRG.17585.1	0.0833	6.0233	−6.18	7.85 × 10^−6^	1.32× 10^−3^
	MSTRG.9309.1	0.0001	8.9167	−16.44	2.67 × 10^−14^	2.35 × 10^−11^
	MSTRG.15980.1	3.5233	9.9133	−1.49	8.01 × 10^−9^	2.80 × 10^−6^
	MSTRG.18159.11	0.2600	5.8400	−4.49	2.94 × 10^−7^	7.31 × 10^−5^

**Table 3:** Core lncRNAs in the cerebral cortex and hippocampus of 7-month-old 5×FAD mice.

lncRNA ID	Cortex				Hippocampus			
	Log_2_ Fold Change	*p*-Value	*q*-Value	Regulation	Log_2_ Fold Change	*p*-Value	*q*-Value	Regulation
ENSMUST00000127786	11.46	3.74 × 10^−9^	1.57 × 10^−6^	up	10.49	5.72 × 10^−11^	2.86 × 10^−8^	up
ENSMUSG00000098912	−16.56	6.00 × 10^−12^	3.79 × 10^−9^	down	−1.68	1.49 × 10^−5^	2.26 × 10^−3^	down
MSTRG.11359.1	16.09	2.38 × 10^−9^	1.04 × 10^−6^	up	8.62	3.67 × 10^−9^	1.36 × 10^−6^	up
MSTRG.17500.2	16.40	1.44 × 10^−29^	5.35 × 10^−26^	up	15.82	1.77 × 10^−26^	7.06 × 10^−23^	up
MSTRG.16327.2	16.42	3.75 × 10^−6^	7.06 × 10^−4^	up	16.22	1.56 × 10^−5^	2.36 × 10^−3^	up
MSTRG.14838.1	17.10	1.45 × 10^−9^	6.67 × 10^−7^	up	7.44	1.23 × 10^−7^	3.31 × 10^−5^	up
MSTRG.1243.32	16.63	6.51 × 10^−23^	1.43 × 10^−19^	up	16.43	4.48 × 10^−23^	1.22 × 10^−19^	up

**Table 4:** Interaction relationships in the L-M-T network.

lncRNA	miRNA	mRNA
ENSMUST00000127786	miR-362-3p, miR-329-3p, miR-466i-3p, miR-669b-5p, miR-3057-5p, miR-5101, miR-15a-5p, miR-466o-3p, miR-466m-3p, miR-539-5p	Clec7a, 5730455P16Rik, Fnbp1, Elovl2
MSTRG.17500.2		
ENSMUST00000184170	miR-669b-5p, miR-15a-5p	5730455P16Rik, Fnbp1
MSTRG.1243.32	miR-362-3p, miR-329-3p, miR-466i-3p, miR-5101, miR-466o-3p, miR-466m-3p, miR-539-5p	Clec7a, 5730455P16Rik, Fnbp1, Elovl2
MSTRG.16327.2	miR-3057-5p	5730455P16Rik

**Table 5:** Sequences of the primers used in this study.

Name	Sequence (5′-3′)
miR-15a-5p RT	GTCGTATCCAGTGCAGGGTCCGAGGTATTCGCACTGGATACGACCACAAA
miR-15a-5p-F	CGCGTAGCAGCACATAATGG
miR-329-3p RT	GTCGTATCCAGTGCAGGGTCCGAGGTATTCGCACTGGATACGACAAAAAG
miR-329-3p-F	CGCGAACACACCCAGCTAAC
miR-466m-3p RT	GTCGTATCCAGTGCAGGGTCCGAGGTATTCGCACTGGATACGACTGCGTG
miR-466m-3p-F	CGCGCGTACATACACACATACA
miR-466o-3p RT	GTCGTATCCAGTGCAGGGTCCGAGGTATTCGCACTGGATACGACGTCTTA
miR-466o-3p-F	GCGCGTACATACATGCACACA
miR-539-5p RT	GTCGTATCCAGTGCAGGGTCCGAGGTATTCGCACTGGATACGACACACAC
miR-539-5p-F	CGCGGGAGAAATTATCCTTG
miR-669b-5p RT	GTCGTATCCAGTGCAGGGTCCGAGGTATTCGCACTGGATACGACACATGC
miR-669b-5p-F	CGCGAGTTTTGTGTGCATGT
miR-universal R	AGTGCAGGGTCCGAGGTATT
ENSMUST00000127786-F	TCACTCCTGCCTTTCGTGAC
ENSMUST00000127786-R	AACAAGTGGGGTGAGCACAA
ENSMUSG00000098912-F	CAGAACAAGCAGCAAGGCACAAC
ENSMUSG00000098912-R	AGTCAGAACCCGTGAAAATCAGTCC
Clec7a-F	GTGCAGTAAGCTTTCCTGGG
Clec7a-R	TCCCGCAATCAGAGTGAAG
Fnbp1-F	AGATGGAGGAGAGGCGGATTGTG
Fnbp1-R	TCACTATCCCGTCCAGGCACTTC
Elovl2-F	CAACATGTTTGGACCACGAG
Elovl2-R	GGATGAAGGTGGGAAGGTAAG
5730455P16Rik-F	GGTGATGATTTCAGCCTCTCCTTGG
5730455P16Rik-R	AACATTACCCAGCCCTTCAAAGACC
U6-F	CAAATTCGTGAAGCGTTCCA
U6-R	AGTGCAGGGTCCGAGGTATT
U6-RT	GTCGTATCCAGTGCAGGGTCCGAGGTA
GAPDH-F	TTGATCTGAAGTCAGGAATCCC
GAPDH-R	TGTAGACCATGTAGTTGAGGTCA

## Data Availability

The data in this study that support the findings are available from the corresponding author upon request.

## Supplementary Materials

The supporting information can be downloaded at: https://www.mdpi.com/article/10.3390/ijms23158497/s1.
