# Supplementary material for: The Relationship between the Aberrant Long Non-Coding RNA-Mediated Competitive Endogenous RNA Network and Alzheimer’s Disease Pathogenesis
Source: Int J Mol Sci. 2022 Jul 31;23(15):8497. doi: 10.3390/ijms23158497 (PMC9369371; doi:10.3390/ijms23158497)
Supplement: Supplementary file 1 [file ijms-23-08497-s001.zip › ijms-1780332-supplementary.pdf]

Supplementary Materials

Content

Figure S1. Processing of establishment of potential interactions between lncRNAs, miRNAs, and mRNAs in the cerebral cortex and hippocampus of 5×FAD mice.. 2
Table S1. Quality assessment of 12 cDNA libraries associated with lncRNAs. 3
Table S2. Information of the upregulated lncRNA-miRNA network in the cortex of 7-month-old 5×FAD mice. 4
Table S3. Information of the downregulated lncRNA-miRNA network in the cortex of 7-month-old 5×FAD mice. 36
Table S4. Correlated information of the upregulated lncRNA-associated ceRNA network in the cerebral cortex of 5×FAD mice. 47
Table S5. Correlated information of the downregulated lncRNA-associated ceRNA network in the cerebral cortex of 5×FAD mice. 50
Table S6. Information of the upregulated lncRNA-miRNA network in the hippocampus of 7-month-old 5×FAD mice. 51
Table S7. Information of the downregulated lncRNA-miRNA network in the hippocampus of 7-month-old 5×FAD mice. 71
Table S8. Correlated information of the upregulated lncRNA-associated ceRNA network in the hippocampus of 5×FAD mice. 85
Table S9. Correlated information of the downregulated lncRNA-associated ceRNA network in the hippocampus of 5×FAD mice. 87
Table S10. Information of the lncRNA-miRNA network in the cerebral cortex and hippocampus of 7-month-old 5×FAD mice. 89
Table S11. Information of RNAs in the L-M-T network based on RNA-seq results. 115

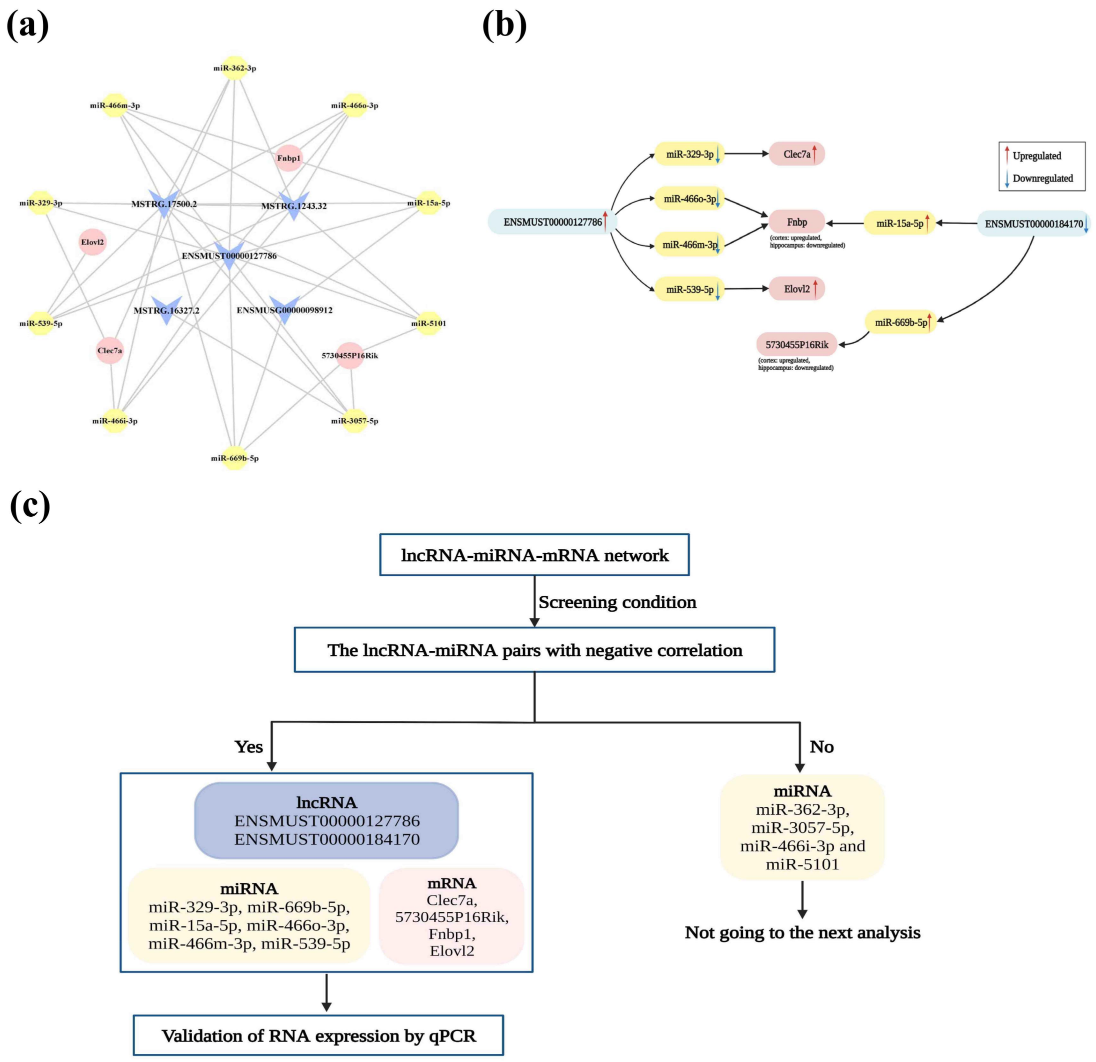

Figure S1. Processing of establishment of potential interactions between lncRNAs, miRNAs, and mRNAs in the cerebral cortex and hippocampus of 5×FAD mice. (a) Establishment of the L-M-T network based on five lncRNAs, ten miRNAs, and four mRNAs. The lncRNAs, miRNAs, and mRNAs are indicated as V-shapes, octagons, and circular shapes, respectively. (b) Potential interactions between screened lncRNAs, miRNAs, and mRNAs in the L-M-T network. The arrows indicate the potential effect of one RNA on another RNA. (c) Workflow of screened RNAs for qPCR validation. lncRNA: long non-coding RNA; miRNA: microRNA; mRNA: messenger RNA; qPCR: quantitative real-time Polymerase Chain Reaction.

Table S1. Quality assessment of 12 cDNA libraries associated with lncRNAs.

Samples	Total Reads Count	Total Bases Count (bp)	Average Read Length (bp)	N Bases Count (bp)	N Bases Ratio (%)	GC Bases Count (bp)	GC Bases Ratio (%)
Cerebral cortex of 7-month-old WT mice-1	70,848,618	10,627,292,700	150	198,826	0.00	5,216,312,261	49.08
Cerebral cortex of 7-month-old WT mice-2	73,438,846	11,015,826,900	150	219,101	0.00	5,345,363,860	48.52
Cerebral cortex of 7-month-old WT mice-3	54,045,806	8,106,870,900	150	196,809	0.00	3,805,904,026	46.95
Cerebral cortex of 7-month-old 5×FAD mice-1	70,387,314	10,558,097,100	150	234,038	0.00	5,072,148,931	48.04
Cerebral cortex of 7-month-old 5×FAD mice-2	59,014,786	8,852,217,900	150	205,973	0.00	4,217,932,828	47.65
Cerebral cortex of 7-month-old 5×FAD mice-3	70,632,868	10,594,930,200	150	214,497	0.00	5,095,059,928	48.09
Hippocampus of 7-month-old WT mice-1	77,487,284	11,623,092,600	150	300,138	0.00	5,528,175,705	47.56
Hippocampus of 7-month-old WT mice-2	77,478,166	11,621,724,900	150	279,909	0.00	5,488,985,013	47.23
Hippocampus of 7-month-old WT mice-3	72,130,852	10,819,627,800	150	266,620	0.00	5,191,919,471	47.99
Hippocampus of 7-month-old 5×FAD mice-1	59,970,682	8,995,602,300	150	180,081	0.00	4,369,498,492	48.57
Hippocampus of 7-month-old 5×FAD mice-2	69,198,998	10,379,849,700	150	208,504	0.00	4,884,950,222	47.06
Hippocampus of 7-month-old 5×FAD mice-3	62,388,494	9,358,274,100	150	222,352	0.00	4,585,485,011	49.00

Table S2. Information of the upregulated lncRNA-miRNA network in the cortex of 7-month-old 5×FAD mice.

Upregulated lncRNA	miRNA targets
ENSMUST00000127786	miR-466m-3p
ENSMUST00000127786	miR-669a-3-3p
ENSMUST00000127786	miR-669c-3p
ENSMUST00000127786	miR-466o-3p
ENSMUST00000127786	miR-98-5p
ENSMUST00000127786	miR-3473d
ENSMUST00000127786	miR-686
ENSMUST00000127786	miR-6904-5p
ENSMUST00000127786	miR-6984-5p
ENSMUST00000127786	miR-6240
ENSMUST00000127786	miR-466b-3p
ENSMUST00000127786	miR-466p-3p
ENSMUST00000127786	miR-3060-3p
ENSMUST00000127786	miR-466c-3p
ENSMUST00000127786	miR-669i
ENSMUST00000127786	miR-466n-3p
ENSMUST00000127786	miR-669p-3p
ENSMUST00000127786	miR-7000-5p
ENSMUST00000127786	miR-6914-5p
ENSMUST00000127786	miR-186-5p
ENSMUST00000127786	miR-7092-3p
ENSMUST00000127786	miR-448-3p
ENSMUST00000127786	miR-494-3p
ENSMUST00000127786	miR-330-3p
ENSMUST00000127786	miR-3569-5p
ENSMUST00000127786	miR-30c-2-3p
ENSMUST00000127786	miR-26a-5p
ENSMUST00000127786	miR-107-3p
ENSMUST00000127786	miR-760-5p
ENSMUST00000127786	miR-103-3p
ENSMUST00000127786	miR-6916-3p
ENSMUST00000127786	miR-6927-3p
ENSMUST00000127786	miR-7116-5p
ENSMUST00000127786	let-7a-5p
ENSMUST00000127786	let-7d-5p
ENSMUST00000127786	miR-6970-5p
ENSMUST00000127786	miR-543-3p
ENSMUST00000127786	miR-221-5p
ENSMUST00000127786	miR-132-3p
ENSMUST00000127786	miR-23b-3p
ENSMUST00000127786	miR-195a-5p

ENSMUST00000127786	miR-129b-3p
ENSMUST00000127786	miR-1191b-5p
ENSMUST00000127786	miR-6982-3p
ENSMUST00000127786	miR-425-5p
ENSMUST00000127786	miR-543-5p
ENSMUST00000127786	miR-148b-3p
ENSMUST00000127786	miR-3069-5p
ENSMUST00000127786	miR-466d-3p
ENSMUST00000127786	miR-3095-5p
ENSMUST00000127786	miR-466a-3p
ENSMUST00000127786	miR-27b-3p
ENSMUST00000127786	miR-466e-3p
ENSMUST00000127786	miR-6933-3p
ENSMUST00000127786	miR-6925-5p
ENSMUST00000127786	miR-201-5p
ENSMUST00000127786	miR-3474
ENSMUST00000127786	let-7g-5p
ENSMUST00000127786	miR-551b-5p
ENSMUST00000127786	miR-1933-5p
ENSMUST00000127786	miR-342-3p
ENSMUST00000127786	miR-410-3p
ENSMUST00000127786	miR-1949
ENSMUST00000127786	miR-26b-5p
ENSMUST00000127786	miR-6937-5p
ENSMUST00000127786	miR-3064-5p
ENSMUST00000127786	miR-5615-5p
ENSMUST00000127786	miR-6971-3p
ENSMUST00000127786	miR-344-5p
ENSMUST00000127786	miR-6999-5p
ENSMUST00000127786	miR-704
ENSMUST00000127786	miR-5101
ENSMUST00000127786	miR-148b-5p
ENSMUST00000127786	miR-1981-5p
ENSMUST00000127786	miR-7066-3p
ENSMUST00000127786	miR-3082-5p
ENSMUST00000127786	miR-423-5p
ENSMUST00000127786	miR-6516-5p
ENSMUST00000127786	miR-30c-1-3p
ENSMUST00000127786	miR-486a-3p
ENSMUST00000127786	miR-1961
ENSMUST00000127786	miR-124-3p
ENSMUST00000127786	miR-145a-5p
ENSMUST00000127786	miR-7665-5p
ENSMUST00000127786	miR-135a-2-3p

ENSMUST00000127786	miR-5114
ENSMUST00000127786	miR-3089-5p
ENSMUST00000127786	miR-23a-3p
ENSMUST00000127786	miR-329-3p
ENSMUST00000127786	miR-15b-5p
ENSMUST00000127786	miR-31-5p
ENSMUST00000127786	miR-467a-3p
ENSMUST00000127786	miR-7661-3p
ENSMUST00000127786	miR-6911-5p
ENSMUST00000127786	miR-3083-5p
ENSMUST00000127786	miR-29b-3p
ENSMUST00000127786	miR-674-5p
ENSMUST00000127786	miR-203-3p
ENSMUST00000127786	miR-421-3p
ENSMUST00000127786	miR-654-3p
ENSMUST00000127786	miR-5124b
ENSMUST00000127786	miR-3074-5p
ENSMUST00000127786	miR-130b-5p
ENSMUST00000127786	let-7f-5p
ENSMUST00000127786	miR-128-3p
ENSMUST00000127786	let-7e-5p
ENSMUST00000127786	miR-3080-5p
ENSMUST00000127786	miR-466f-3p
ENSMUST00000127786	miR-1943-5p
ENSMUST00000127786	miR-6982-5p
ENSMUST00000127786	miR-669f-3p
ENSMUST00000127786	miR-6976-5p
ENSMUST00000127786	miR-694
ENSMUST00000127786	miR-5617-5p
ENSMUST00000127786	miR-3094-3p
ENSMUST00000127786	miR-5709-5p
ENSMUST00000127786	miR-344d-1-5p
ENSMUST00000127786	let-7i-5p
ENSMUST00000127786	miR-3065-5p
ENSMUST00000127786	miR-672-3p
ENSMUST00000127786	miR-7236-3p
ENSMUST00000127786	miR-1843a-5p
ENSMUST00000127786	miR-192-5p
ENSMUST00000127786	miR-219b-3p
ENSMUST00000127786	miR-1912-3p
ENSMUST00000127786	miR-204-3p
ENSMUST00000127786	miR-7087-5p
ENSMUST00000127786	miR-30a-3p
ENSMUST00000127786	miR-7054-5p

ENSMUST00000127786	miR-6537-3p
ENSMUST00000127786	miR-192-3p
ENSMUST00000127786	miR-7022-5p
ENSMUST00000127786	miR-505-3p
ENSMUST00000127786	miR-501-5p
ENSMUST00000127786	miR-6903-3p
ENSMUST00000127786	miR-24-3p
ENSMUST00000127786	miR-1934-5p
ENSMUST00000127786	miR-185-3p
ENSMUST00000127786	miR-7a-5p
ENSMUST00000127786	miR-8118
ENSMUST00000127786	let-7f-2-3p
ENSMUST00000127786	miR-486b-3p
ENSMUST00000127786	miR-335-3p
ENSMUST00000127786	miR-344d-3-5p
ENSMUST00000127786	miR-5710
ENSMUST00000127786	miR-8106
ENSMUST00000127786	miR-409-5p
ENSMUST00000127786	miR-503-3p
ENSMUST00000127786	miR-382-3p
ENSMUST00000127786	miR-7647-3p
ENSMUST00000127786	miR-6947-5p
ENSMUST00000127786	miR-344c-5p
ENSMUST00000127786	miR-212-3p
ENSMUST00000127786	miR-6964-3p
ENSMUST00000127786	miR-29a-3p
ENSMUST00000127786	miR-384-5p
ENSMUST00000127786	miR-1955-3p
ENSMUST00000127786	miR-222-3p
ENSMUST00000127786	miR-1931
ENSMUST00000127786	miR-3087-3p
ENSMUST00000127786	miR-3099-3p
ENSMUST00000127786	miR-466i-3p
ENSMUST00000127786	miR-674-3p
ENSMUST00000127786	miR-7117-5p
ENSMUST00000127786	miR-7a-2-3p
ENSMUST00000127786	miR-3473e
ENSMUST00000127786	miR-3102-3p
ENSMUST00000127786	miR-1969
ENSMUST00000127786	miR-299a-5p
ENSMUST00000127786	miR-1298-5p
ENSMUST00000127786	miR-1264-3p
ENSMUST00000127786	miR-8111
ENSMUST00000127786	miR-344g-5p

ENSMUST00000127786	miR-3095-3p
ENSMUST00000127786	miR-215-5p
ENSMUST00000127786	miR-3475-3p
ENSMUST00000127786	miR-153-5p
ENSMUST00000127786	miR-7061-5p
ENSMUST00000127786	miR-7015-3p
ENSMUST00000127786	miR-145a-3p
ENSMUST00000127786	miR-7224-3p
ENSMUST00000127786	miR-511-5p
ENSMUST00000127786	miR-221-3p
ENSMUST00000127786	miR-20b-5p
ENSMUST00000127786	miR-1298-3p
ENSMUST00000127786	miR-504-5p
ENSMUST00000127786	miR-6914-3p
ENSMUST00000127786	miR-7066-5p
ENSMUST00000127786	miR-30c-5p
ENSMUST00000127786	miR-1198-5p
ENSMUST00000127786	miR-7116-3p
ENSMUST00000127786	miR-7013-5p
ENSMUST00000127786	miR-30f
ENSMUST00000127786	miR-7b-5p
ENSMUST00000127786	miR-6997-5p
ENSMUST00000127786	miR-3473b
ENSMUST00000127786	miR-874-3p
ENSMUST00000127786	miR-26b-3p
ENSMUST00000127786	miR-493-5p
ENSMUST00000127786	miR-3058-5p
ENSMUST00000127786	miR-1983
ENSMUST00000127786	miR-7687-3p
ENSMUST00000127786	miR-6902-3p
ENSMUST00000127786	miR-3103-3p
ENSMUST00000127786	miR-181b-5p
ENSMUST00000127786	miR-222-5p
ENSMUST00000127786	miR-6911-3p
ENSMUST00000127786	miR-7240-5p
ENSMUST00000127786	miR-7031-5p
ENSMUST00000127786	miR-3079-3p
ENSMUST00000127786	miR-329-5p
ENSMUST00000127786	miR-412-3p
ENSMUST00000127786	miR-1224-5p
ENSMUST00000127786	miR-466q
ENSMUST00000127786	miR-7077-5p
ENSMUST00000127786	miR-3062-5p
ENSMUST00000127786	miR-322-3p

ENSMUST00000127786	miR-328-3p
ENSMUST00000127786	miR-26a-2-3p
ENSMUST00000127786	miR-223-5p
ENSMUST00000127786	miR-3472
ENSMUST00000127786	miR-7242-3p
ENSMUST00000127786	miR-129-2-3p
ENSMUST00000127786	miR-7085-5p
ENSMUST00000127786	miR-186-3p
ENSMUST00000127786	miR-3081-3p
ENSMUST00000127786	miR-324-3p
ENSMUST00000127786	miR-3078-5p
ENSMUST00000127786	miR-6991-5p
ENSMUST00000127786	miR-344d-3p
ENSMUST00000127786	miR-30d-5p
ENSMUST00000127786	miR-302b-3p
ENSMUST00000127786	miR-320-3p
ENSMUST00000127786	miR-181b-2-3p
ENSMUST00000127786	miR-7004-5p
ENSMUST00000127786	miR-3085-3p
ENSMUST00000127786	miR-384-3p
ENSMUST00000127786	miR-6399
ENSMUST00000127786	miR-30e-3p
ENSMUST00000127786	miR-351-5p
ENSMUST00000127786	miR-7019-3p
ENSMUST00000127786	miR-7085-3p
ENSMUST00000127786	miR-6516-3p
ENSMUST00000127786	miR-129-1-3p
ENSMUST00000127786	miR-361-5p
ENSMUST00000127786	miR-3105-3p
ENSMUST00000127786	miR-7651-5p
ENSMUST00000127786	miR-125a-5p
ENSMUST00000127786	miR-8099
ENSMUST00000127786	miR-6975-3p
ENSMUST00000127786	miR-483-3p
ENSMUST00000127786	miR-3091-3p
ENSMUST00000127786	miR-683
ENSMUST00000127786	miR-483-5p
ENSMUST00000127786	miR-450b-3p
ENSMUST00000127786	miR-3102-5p.2-5p
ENSMUST00000127786	miR-135b-3p
ENSMUST00000127786	miR-450a-1-3p
ENSMUST00000127786	miR-345-3p
ENSMUST00000127786	miR-667-3p
ENSMUST00000127786	miR-3113-5p

ENSMUST00000127786	miR-5623-5p
ENSMUST00000127786	miR-6540-5p
ENSMUST00000127786	miR-5626-5p
ENSMUST00000127786	miR-505-5p
ENSMUST00000127786	miR-338-5p
ENSMUST00000127786	miR-664-3p
ENSMUST00000127786	miR-501-3p
ENSMUST00000127786	miR-664-5p
ENSMUST00000127786	miR-3094-5p
ENSMUST00000127786	miR-450a-2-3p
ENSMUST00000127786	miR-539-5p
ENSMUST00000127786	miR-411-3p
ENSMUST00000127786	miR-379-3p
ENSMUST00000127786	miR-682
ENSMUST00000127786	miR-3080-3p
ENSMUST00000127786	miR-9-3p
ENSMUST00000127786	miR-22-3p
ENSMUST00000127786	miR-185-5p
ENSMUST00000127786	miR-331-3p
ENSMUST00000127786	miR-5127
ENSMUST00000127786	miR-486a-5p
ENSMUST00000127786	miR-486b-5p
ENSMUST00000127786	miR-191-5p
ENSMUST00000127786	miR-125b-5p
ENSMUST00000127786	miR-679-3p
ENSMUST00000127786	miR-466l-3p
ENSMUST00000127786	miR-143-3p
ENSMUST00000127786	miR-127-3p
ENSMUST00000182231	miR-191-3p
ENSMUST00000182231	miR-7236-3p
ENSMUST00000182231	miR-329-3p
ENSMUST00000182231	miR-3082-5p
ENSMUST00000182231	miR-3091-3p
ENSMUST00000182231	miR-1964-5p
ENSMUST00000182231	miR-346-5p
ENSMUST00000182231	miR-331-3p
ENSMUST00000141677	miR-672-3p
ENSMUST00000141677	miR-301a-5p
ENSMUST00000141677	miR-669c-3p
ENSMUST00000141677	miR-27b-3p
ENSMUST00000141677	miR-7047-5p
ENSMUST00000141677	miR-382-3p
ENSMUST00000141677	miR-128-3p
ENSMUST00000131052	miR-7019-3p

ENSMUST00000131052	miR-760-3p
ENSMUST00000131052	miR-3470a
ENSMUST00000131052	miR-7046-3p
ENSMUST00000189564	miR-3095-3p
ENSMUST00000189564	miR-335-3p
ENSMUST00000189564	miR-6540-3p
ENSMUST00000189564	miR-450b-5p
ENSMUST00000189564	miR-7235-3p
ENSMUST00000189564	miR-466f-3p
ENSMUST00000189564	miR-6925-5p
ENSMUST00000189564	miR-467f
ENSMUST00000189564	miR-7668-5p
ENSMUST00000189564	miR-223-5p
ENSMUST00000189564	miR-6984-5p
ENSMUST00000189564	miR-301a-5p
ENSMUST00000189564	miR-669h-3p
ENSMUST00000189564	miR-669f-3p
ENSMUST00000189564	miR-467a-3p
ENSMUST00000189564	miR-7674-5p
ENSMUST00000189564	miR-6915-3p
ENSMUST00000189564	miR-1668
ENSMUST00000189564	miR-329-5p
ENSMUST00000189564	miR-3085-3p
ENSMUST00000189564	miR-3064-5p
ENSMUST00000189564	miR-1b-3p
ENSMUST00000189564	miR-874-3p
ENSMUST00000189564	miR-450b-3p
ENSMUST00000189564	miR-450a-1-3p
ENSMUST00000189564	miR-323-5p
ENSMUST00000155277	miR-7231-5p
ENSMUST00000155277	miR-466n-3p
ENSMUST00000155277	miR-669c-3p
ENSMUST00000155277	miR-329-3p
ENSMUST00000155277	miR-467e-3p
ENSMUST00000155277	miR-467d-3p
ENSMUST00000155277	miR-467c-3p
ENSMUST00000155277	miR-467b-3p
ENSMUST00000155277	miR-6952-5p
ENSMUST00000155277	miR-450a-2-3p
ENSMUST00000155277	miR-466e-3p
ENSMUST00000155277	miR-466d-3p
ENSMUST00000155277	miR-466a-3p
ENSMUST00000155277	miR-6399
ENSMUST00000155277	miR-3084-3p

ENSMUST00000155277	miR-6955-5p
ENSMUST00000155277	miR-7084-5p
ENSMUST00000155277	miR-8118
ENSMUST00000155277	miR-3062-5p
ENSMUST00000155277	miR-7038-5p
ENSMUST00000155277	let-7c-2-3p
ENSMUST00000155277	let-7a-1-3p
ENSMUST00000155277	miR-6946-3p
ENSMUST00000155277	miR-191-3p
ENSMUST00000155277	miR-3470a
ENSMUST00000155277	miR-299a-5p
ENSMUST00000123670	miR-186-3p
ENSMUST00000123670	miR-6963-3p
ENSMUST00000123670	miR-491-5p
ENSMUST00000123670	miR-7052-3p
ENSMUST00000123670	miR-7051-5p
ENSMUST00000150644	miR-129b-5p
ENSMUST00000150644	miR-7065-3p
ENSMUST00000150644	miR-7077-5p
ENSMUST00000150644	miR-1a-3p
ENSMUST00000150644	miR-7052-3p
ENSMUST00000150644	miR-7224-3p
ENSMUST00000150644	miR-7651-5p
ENSMUST00000150644	miR-671-5p
ENSMUST00000150644	miR-496a-5p
ENSMUST00000150644	miR-683
ENSMUST00000150644	miR-370-3p
ENSMUST00000156420	miR-6925-5p
ENSMUST00000156420	miR-107-3p
ENSMUST00000156420	miR-103-3p
ENSMUST00000156420	miR-7665-5p
ENSMUST00000156420	miR-6976-5p
ENSMUST00000156420	miR-5125
ENSMUST00000156420	miR-7092-5p
ENSMUST00000156420	miR-466q
ENSMUST00000156420	miR-7031-5p
ENSMUST00000156420	miR-7027-3p
ENSMUST00000156420	miR-195a-5p
ENSMUST00000156420	miR-15b-5p
ENSMUST00000156420	miR-704
ENSMUST00000221971	miR-6975-3p
ENSMUST00000221971	miR-382-3p
ENSMUST00000221971	miR-320-5p
ENSMUST00000221971	miR-22-3p

ENSMUST00000221971	miR-3475-3p
ENSMUST00000221971	miR-683
ENSMUST00000221971	miR-6983-5p
ENSMUST00000221971	miR-181b-2-3p
ENSMUST00000221971	miR-6946-3p
ENSMUST00000221971	miR-7047-5p
ENSMUST00000221971	miR-466e-3p
ENSMUST00000221971	miR-466d-3p
ENSMUST00000221971	miR-466a-3p
ENSMUST00000221971	miR-130b-5p
ENSMUST00000221971	miR-669p-3p
ENSMUST00000221971	miR-3103-5p
ENSMUST00000221971	miR-23b-3p
ENSMUST00000221971	miR-23a-3p
ENSMUST00000221971	miR-1943-5p
ENSMUST00000221971	miR-6546-3p
ENSMUST00000205311	miR-409-5p
ENSMUST00000205311	miR-758-5p
ENSMUST00000205311	miR-6946-3p
ENSMUST00000205311	miR-339-3p
ENSMUST00000205311	miR-7085-5p
ENSMUST00000205311	miR-491-5p
ENSMUST00000205311	miR-3069-5p
ENSMUST00000205311	miR-543-3p
ENSMUST00000205311	miR-148b-3p
ENSMUST00000205311	miR-669o-5p
ENSMUST00000126335	miR-379-5p
ENSMUST00000126335	miR-760-3p
ENSMUST00000126335	miR-6927-5p
ENSMUST00000126335	miR-5129-5p
ENSMUST00000126335	miR-1a-3p
ENSMUST00000126335	miR-320-5p
ENSMUST00000126335	miR-92b-5p
ENSMUST00000126335	miR-5101
ENSMUST00000126335	miR-491-5p
ENSMUST00000126335	miR-126a-5p
ENSMUST00000126335	miR-6516-5p
ENSMUST00000126335	miR-682
ENSMUST00000126335	miR-323-5p
ENSMUST00000126335	miR-7034-5p
ENSMUST00000126335	miR-712-5p
ENSMUST00000111019	miR-669f-3p
ENSMUST00000111019	miR-467a-3p
ENSMUST00000111019	miR-466e-3p

ENSMUST00000111019	miR-466d-3p
ENSMUST00000111019	miR-466a-3p
ENSMUST00000111019	miR-8094
ENSMUST00000111019	miR-100-3p
ENSMUST00000111019	miR-335-3p
ENSMUST00000111019	miR-467e-3p
ENSMUST00000111019	miR-467d-3p
ENSMUST00000111019	miR-467c-3p
ENSMUST00000111019	miR-467b-3p
ENSMUST00000111019	miR-6948-5p
ENSMUST00000111019	miR-212-5p
ENSMUST00000111019	miR-384-5p
ENSMUST00000111019	miR-30d-5p
ENSMUST00000111019	miR-30c-5p
ENSMUST00000111019	miR-6946-3p
ENSMUST00000111019	miR-6908-3p
ENSMUST00000111019	miR-7a-1-3p
ENSMUST00000111019	miR-686
ENSMUST00000111019	miR-344h-3p
ENSMUST00000111019	miR-3965
ENSMUST00000111019	miR-3087-3p
ENSMUST00000160318	miR-671-3p
ENSMUST00000160318	miR-7b-5p
ENSMUST00000160318	miR-7a-5p
ENSMUST00000160318	miR-3083-3p
ENSMUST00000148373	miR-6971-3p
ENSMUST00000148373	miR-370-3p
ENSMUST00000148373	miR-5134-5p
ENSMUST00000148373	miR-3099-5p
ENSMUST00000148373	miR-3473e
ENSMUST00000148373	miR-3473b
ENSMUST00000148373	miR-484
ENSMUST00000148373	miR-7062-5p
ENSMUST00000205779	miR-1194
ENSMUST00000205779	miR-6946-3p
ENSMUST00000205779	miR-505-3p
ENSMUST00000205779	miR-3965
ENSMUST00000205779	miR-7064-3p
ENSMUST00000205779	miR-3065-5p
ENSMUST00000205779	miR-669c-3p
ENSMUST00000205779	miR-692
ENSMUST00000205779	miR-3057-3p
ENSMUST00000205779	miR-5615-5p
ENSMUST00000205779	miR-186-5p

ENSMUST00000205779	miR-686
ENSMUST00000145025	miR-504-3p
ENSMUST00000145025	miR-3099-5p
ENSMUST00000145025	miR-29b-1-5p
ENSMUST00000145025	miR-7084-5p
ENSMUST00000145025	miR-23b-3p
ENSMUST00000145025	miR-23a-3p
ENSMUST00000145025	miR-1948-3p
ENSMUST00000145025	miR-6537-3p
ENSMUST00000145025	miR-7073-5p
ENSMUST00000145025	miR-6958-3p
MSTRG.10455.14	miR-7054-5p
MSTRG.10455.14	miR-6999-5p
MSTRG.10455.14	miR-466k
MSTRG.10455.14	miR-153-5p
MSTRG.10455.14	miR-6903-3p
MSTRG.10455.14	miR-551b-5p
MSTRG.10455.14	miR-1954
MSTRG.10455.14	miR-5123
MSTRG.10455.14	miR-30f
MSTRG.10455.14	miR-701-3p
MSTRG.10455.14	miR-7092-3p
MSTRG.10455.14	miR-7081-3p
MSTRG.10455.14	miR-1961
MSTRG.10455.14	miR-335-3p
MSTRG.10455.14	miR-7685-5p
MSTRG.10455.14	miR-669c-3p
MSTRG.10455.14	miR-6975-3p
MSTRG.10455.14	miR-6915-3p
MSTRG.10455.14	miR-384-3p
MSTRG.10455.14	miR-547-3p
MSTRG.10455.14	miR-3102-5p.2-5p
MSTRG.10455.14	miR-466e-3p
MSTRG.10455.14	miR-466d-3p
MSTRG.10455.14	miR-466a-3p
MSTRG.10455.14	miR-3569-3p
MSTRG.10455.14	miR-203-3p
MSTRG.10455.14	miR-320-5p
MSTRG.10455.14	miR-3069-5p
MSTRG.10455.14	miR-412-3p
MSTRG.10455.14	miR-1983
MSTRG.10455.14	miR-7013-5p
MSTRG.10455.14	miR-7668-5p
MSTRG.10455.14	miR-7a-1-3p

MSTRG.10455.14	miR-126a-5p
MSTRG.10455.14	miR-322-3p
MSTRG.10455.14	miR-3089-5p
MSTRG.10455.14	miR-6970-5p
MSTRG.10455.14	miR-466n-3p
MSTRG.10455.14	miR-98-5p
MSTRG.10455.14	let-7i-5p
MSTRG.10455.14	let-7g-5p
MSTRG.10455.14	let-7f-5p
MSTRG.10455.14	let-7e-5p
MSTRG.10455.14	let-7a-5p
MSTRG.10455.14	let-7d-5p
MSTRG.10455.14	miR-3057-3p
MSTRG.10455.14	miR-3094-3p
MSTRG.10455.14	miR-504-3p
MSTRG.10455.14	miR-3572-3p
MSTRG.10455.14	miR-148b-5p
MSTRG.10455.14	miR-6516-3p
MSTRG.10455.14	let-7f-2-3p
MSTRG.10455.14	miR-712-5p
MSTRG.10455.14	miR-1298-5p
MSTRG.10455.14	miR-6916-3p
MSTRG.10455.14	miR-6946-3p
MSTRG.10455.14	miR-467e-3p
MSTRG.10455.14	miR-467d-3p
MSTRG.10455.14	miR-467c-3p
MSTRG.10455.14	miR-467b-3p
MSTRG.10455.14	miR-6948-5p
MSTRG.10455.14	miR-222-3p
MSTRG.10455.14	miR-221-3p
MSTRG.10455.14	let-7c-2-3p
MSTRG.10455.14	let-7a-1-3p
MSTRG.10455.14	miR-6984-5p
MSTRG.10455.14	miR-466l-3p
MSTRG.10455.14	let-7j
MSTRG.10455.14	miR-488-5p
MSTRG.10455.14	miR-6984-3p
MSTRG.10455.14	miR-6947-5p
MSTRG.10455.14	miR-686
MSTRG.10455.14	miR-669p-3p
MSTRG.10455.14	miR-6537-3p
MSTRG.10455.14	miR-204-5p
MSTRG.10455.14	miR-7116-5p
MSTRG.10455.14	miR-344h-5p

MSTRG.10455.14	miR-344e-5p
MSTRG.10455.14	miR-325-3p
MSTRG.10455.14	miR-1195
MSTRG.10455.14	miR-694
MSTRG.10455.14	miR-7038-5p
MSTRG.10455.14	miR-669h-3p
MSTRG.10455.14	miR-6238
MSTRG.10455.14	miR-669f-3p
MSTRG.10455.14	miR-467a-3p
MSTRG.10455.14	miR-6240
MSTRG.10455.14	miR-330-3p
MSTRG.10455.14	miR-338-5p
MSTRG.10455.14	miR-194-5p
MSTRG.11359.1	miR-7667-3p
MSTRG.11359.1	miR-365-2-5p
MSTRG.11359.1	miR-365-1-5p
MSTRG.15393.1	miR-669c-3p
MSTRG.15393.1	miR-495-3p
MSTRG.15393.1	miR-3103-3p
MSTRG.15393.1	miR-8118
MSTRG.15393.1	miR-466l-3p
MSTRG.15393.1	miR-6908-5p
MSTRG.15393.1	miR-466i-3p
MSTRG.15393.1	miR-8106
MSTRG.15393.1	miR-3091-3p
MSTRG.15393.1	miR-694
MSTRG.15393.1	miR-664-3p
MSTRG.15393.1	miR-31-5p
MSTRG.15393.1	miR-7069-5p
MSTRG.15393.1	miR-423-5p
MSTRG.15393.1	miR-5114
MSTRG.15393.1	miR-1195
MSTRG.15393.1	miR-1933-5p
MSTRG.15393.1	miR-7668-5p
MSTRG.17500.2	miR-669c-3p
MSTRG.17500.2	miR-466k
MSTRG.17500.2	miR-6903-3p
MSTRG.17500.2	miR-7116-3p
MSTRG.17500.2	miR-223-5p
MSTRG.17500.2	miR-1187
MSTRG.17500.2	miR-466p-5p
MSTRG.17500.2	miR-466a-5p
MSTRG.17500.2	miR-466e-5p
MSTRG.17500.2	miR-466o-3p

MSTRG.17500.2	miR-466m-3p
MSTRG.17500.2	miR-7116-5p
MSTRG.17500.2	miR-466l-3p
MSTRG.17500.2	miR-5125
MSTRG.17500.2	miR-7117-5p
MSTRG.17500.2	miR-3473e
MSTRG.17500.2	miR-3473b
MSTRG.17500.2	miR-6944-3p
MSTRG.17500.2	miR-3083-5p
MSTRG.17500.2	miR-7240-5p
MSTRG.17500.2	miR-5101
MSTRG.17500.2	miR-672-3p
MSTRG.17500.2	miR-7073-5p
MSTRG.17500.2	miR-185-5p
MSTRG.17500.2	miR-468-5p
MSTRG.17500.2	miR-6948-5p
MSTRG.17500.2	miR-7013-5p
MSTRG.17500.2	miR-466e-3p
MSTRG.17500.2	miR-466d-3p
MSTRG.17500.2	miR-466a-3p
MSTRG.17500.2	miR-7664-3p
MSTRG.17500.2	miR-467e-3p
MSTRG.17500.2	miR-467d-3p
MSTRG.17500.2	miR-467c-3p
MSTRG.17500.2	miR-467b-3p
MSTRG.17500.2	miR-664-5p
MSTRG.17500.2	miR-204-3p
MSTRG.17500.2	miR-145a-3p
MSTRG.17500.2	miR-1954
MSTRG.17500.2	miR-1194
MSTRG.17500.2	miR-654-3p
MSTRG.17500.2	miR-6975-3p
MSTRG.17500.2	miR-331-3p
MSTRG.17500.2	miR-5124b
MSTRG.17500.2	miR-24-3p
MSTRG.17500.2	miR-6998-3p
MSTRG.17500.2	miR-1198-5p
MSTRG.17500.2	miR-365-2-5p
MSTRG.17500.2	miR-365-1-5p
MSTRG.17500.2	miR-6908-5p
MSTRG.17500.2	miR-511-3p
MSTRG.17500.2	miR-539-5p
MSTRG.17500.2	miR-139-5p
MSTRG.17500.2	miR-450b-5p

MSTRG.17500.2	miR-6964-3p
MSTRG.17500.2	miR-342-3p
MSTRG.17500.2	miR-344g-5p
MSTRG.17500.2	miR-344d-3-5p
MSTRG.17500.2	miR-344d-1-5p
MSTRG.17500.2	miR-344c-5p
MSTRG.17500.2	miR-344-5p
MSTRG.17500.2	miR-466i-3p
MSTRG.17500.2	miR-3087-3p
MSTRG.17500.2	miR-181d-3p
MSTRG.17500.2	miR-7231-5p
MSTRG.17500.2	miR-6516-3p
MSTRG.17500.2	miR-21b
MSTRG.17500.2	miR-5626-3p
MSTRG.17500.2	miR-363-5p
MSTRG.17500.2	miR-204-5p
MSTRG.17500.2	miR-6402
MSTRG.17500.2	miR-208a-5p
MSTRG.17500.2	miR-7008-3p
MSTRG.17500.2	miR-1964-5p
MSTRG.17500.2	miR-1961
MSTRG.17500.2	miR-6946-3p
MSTRG.17500.2	miR-3099-5p
MSTRG.17500.2	miR-7026-5p
MSTRG.17500.2	miR-185-3p
MSTRG.17500.2	miR-7000-5p
MSTRG.17500.2	miR-433-5p
MSTRG.17500.2	miR-683
MSTRG.17500.2	miR-6900-3p
MSTRG.17500.2	miR-195a-3p
MSTRG.17500.2	miR-532-5p
MSTRG.17500.2	miR-466q
MSTRG.17500.2	miR-7663-3p
MSTRG.17500.2	miR-466n-3p
MSTRG.17500.2	miR-694
MSTRG.17500.2	miR-7667-3p
MSTRG.17500.2	miR-338-5p
MSTRG.17500.2	miR-129b-5p
MSTRG.17500.2	miR-301a-5p
MSTRG.17500.2	miR-145a-5p
MSTRG.17500.2	miR-7686-5p
MSTRG.17500.2	miR-3094-5p
MSTRG.17500.2	miR-3065-5p
MSTRG.17500.2	miR-450a-2-3p

MSTRG.17500.2	miR-7031-5p
MSTRG.17500.2	miR-323-5p
MSTRG.17500.2	miR-3074-5p
MSTRG.17500.2	miR-7052-3p
MSTRG.17500.2	miR-466p-3p
MSTRG.17500.2	miR-466c-3p
MSTRG.17500.2	miR-466b-3p
MSTRG.17500.2	miR-7063-5p
MSTRG.17500.2	miR-329-3p
MSTRG.17500.2	miR-6958-3p
MSTRG.17500.2	miR-1929-3p
MSTRG.17500.2	miR-1193-3p
MSTRG.17500.2	miR-7084-5p
MSTRG.17500.2	miR-466f-3p
MSTRG.17500.2	miR-7015-3p
MSTRG.17500.2	miR-493-5p
MSTRG.17500.2	miR-551b-5p
MSTRG.17500.2	miR-433-3p
MSTRG.17500.2	miR-1943-5p
MSTRG.17500.2	miR-5615-5p
MSTRG.17500.2	miR-7065-3p
MSTRG.17500.2	miR-706
MSTRG.17500.2	miR-222-5p
MSTRG.17500.2	miR-3085-3p
MSTRG.17500.2	miR-3064-5p
MSTRG.17500.2	miR-130b-5p
MSTRG.17500.2	miR-5127
MSTRG.17500.2	miR-195a-5p
MSTRG.17500.2	miR-15b-5p
MSTRG.17500.2	miR-98-5p
MSTRG.17500.2	let-7i-5p
MSTRG.17500.2	let-7g-5p
MSTRG.17500.2	let-7f-5p
MSTRG.17500.2	let-7e-5p
MSTRG.17500.2	let-7a-5p
MSTRG.17500.2	miR-148b-3p
MSTRG.17500.2	miR-669a-3-3p
MSTRG.17500.2	miR-7685-5p
MSTRG.17500.2	miR-3094-3p
MSTRG.17500.2	miR-3095-5p
MSTRG.17500.2	miR-1941-3p
MSTRG.17500.2	miR-1983
MSTRG.17500.2	miR-686
MSTRG.17500.2	miR-488-5p

MSTRG.17500.2	miR-5114
MSTRG.17500.2	miR-212-3p
MSTRG.17500.2	miR-132-3p
MSTRG.17500.2	miR-412-3p
MSTRG.17500.2	miR-7085-3p
MSTRG.17500.2	miR-669h-3p
MSTRG.17500.2	miR-877-5p
MSTRG.17500.2	miR-8106
MSTRG.17500.2	miR-3066-3p
MSTRG.17500.2	miR-365-3p
MSTRG.17500.2	miR-7077-5p
MSTRG.17500.2	miR-7689-3p
MSTRG.17500.2	miR-6901-3p
MSTRG.17500.2	miR-3084-3p
MSTRG.17500.2	miR-692
MSTRG.17500.2	miR-6948-3p
MSTRG.17500.2	miR-7026-3p
MSTRG.17500.2	miR-324-3p
MSTRG.17500.2	miR-384-3p
MSTRG.17500.2	miR-3100-5p
MSTRG.17500.2	miR-377-5p
MSTRG.17500.2	miR-693-5p
MSTRG.17500.2	let-7d-5p
MSTRG.17500.2	miR-7055-3p
MSTRG.17500.2	miR-6540-3p
MSTRG.17500.2	miR-370-5p
MSTRG.17500.2	miR-1b-3p
MSTRG.17500.2	miR-6909-5p
MSTRG.17500.2	miR-770-3p
MSTRG.17500.2	miR-7656-5p
MSTRG.17500.2	miR-6769b-5p
MSTRG.17500.2	miR-1894-5p
MSTRG.17500.2	miR-30e-3p
MSTRG.17500.2	miR-30a-3p
MSTRG.17500.2	let-7j
MSTRG.17500.2	miR-6962-5p
MSTRG.17500.2	miR-504-5p
MSTRG.17500.2	miR-3057-3p
MSTRG.17500.2	miR-873a-3p
MSTRG.17500.2	miR-6998-5p
MSTRG.17500.2	miR-346-5p
MSTRG.17500.2	miR-361-3p
MSTRG.17500.2	miR-7661-3p
MSTRG.17500.2	miR-3099-3p

MSTRG.17500.2	miR-674-5p
MSTRG.17500.2	miR-701-3p
MSTRG.17500.2	miR-186-5p
MSTRG.17500.2	miR-7674-5p
MSTRG.17500.2	miR-5710
MSTRG.12843.10	miR-669c-3p
MSTRG.12843.10	miR-466k
MSTRG.12843.10	miR-5101
MSTRG.12843.10	miR-7116-3p
MSTRG.12843.10	miR-3065-5p
MSTRG.12843.10	miR-6903-3p
MSTRG.12843.10	miR-466l-3p
MSTRG.12843.10	miR-30f
MSTRG.12843.10	miR-203-3p
MSTRG.12843.10	miR-7685-5p
MSTRG.12843.10	miR-467f
MSTRG.12843.10	miR-7013-5p
MSTRG.12843.10	miR-7061-5p
MSTRG.12843.10	miR-6916-3p
MSTRG.12843.10	miR-466q
MSTRG.12843.10	miR-7065-5p
MSTRG.12843.10	miR-20b-5p
MSTRG.12843.10	miR-186-5p
MSTRG.12843.10	miR-495-3p
MSTRG.12843.10	miR-338-5p
MSTRG.12843.10	miR-382-3p
MSTRG.12843.10	miR-8118
MSTRG.12843.10	miR-7a-1-3p
MSTRG.12843.10	miR-7009-5p
MSTRG.12843.10	miR-7047-5p
MSTRG.12843.10	miR-551b-5p
MSTRG.12843.10	miR-6946-3p
MSTRG.12843.10	miR-1929-3p
MSTRG.12843.10	miR-7092-3p
MSTRG.12843.10	miR-6240
MSTRG.12843.10	miR-330-3p
MSTRG.12843.10	miR-335-3p
MSTRG.12843.10	miR-3103-5p
MSTRG.12843.10	miR-1894-5p
MSTRG.12843.10	miR-712-5p
MSTRG.12843.10	miR-664-3p
MSTRG.12843.10	miR-6975-3p
MSTRG.12843.10	miR-494-3p
MSTRG.12843.10	miR-6996-5p

MSTRG.12843.10	miR-669f-3p
MSTRG.12843.10	miR-467a-3p
MSTRG.12843.10	miR-379-5p
MSTRG.12843.10	miR-692
MSTRG.12843.10	miR-7075-3p
MSTRG.12843.10	miR-223-5p
MSTRG.12843.10	miR-3470a
MSTRG.12843.10	miR-1843a-5p
MSTRG.12843.10	miR-325-3p
MSTRG.12843.10	miR-467e-3p
MSTRG.12843.10	miR-467d-3p
MSTRG.12843.10	miR-467c-3p
MSTRG.12843.10	miR-467b-3p
MSTRG.12843.10	miR-138-2-3p
MSTRG.12843.10	let-7c-2-3p
MSTRG.12843.10	let-7a-1-3p
MSTRG.12843.10	miR-1961
MSTRG.12843.10	miR-126a-5p
MSTRG.12843.10	miR-3066-3p
MSTRG.12843.10	miR-129b-5p
MSTRG.12843.10	miR-7008-3p
MSTRG.12843.10	miR-6944-3p
MSTRG.12843.10	miR-6948-5p
MSTRG.12843.10	miR-466f-3p
MSTRG.12843.10	miR-26b-3p
MSTRG.12843.10	miR-26a-2-3p
MSTRG.12843.10	miR-7686-5p
MSTRG.12843.10	miR-682
MSTRG.12843.10	miR-3572-3p
MSTRG.12843.10	miR-6915-3p
MSTRG.12843.10	miR-7b-5p
MSTRG.12843.10	miR-7a-5p
MSTRG.12843.10	miR-8094
MSTRG.12843.10	miR-466e-3p
MSTRG.12843.10	miR-466d-3p
MSTRG.12843.10	miR-466a-3p
MSTRG.12843.10	miR-26a-1-3p
MSTRG.12843.10	miR-450b-3p
MSTRG.12843.10	miR-450a-1-3p
MSTRG.12843.10	miR-6964-3p
MSTRG.12843.10	miR-448-3p
MSTRG.12843.10	miR-6540-3p
MSTRG.12843.10	miR-148b-3p
MSTRG.12843.10	miR-301a-5p

MSTRG.12843.10	miR-505-3p
MSTRG.12843.10	miR-153-5p
MSTRG.12843.10	miR-145a-5p
MSTRG.12843.10	miR-6982-5p
MSTRG.12843.10	miR-28a-3p
MSTRG.12843.10	miR-872-3p
MSTRG.12843.10	miR-6963-3p
MSTRG.12843.10	miR-7027-5p
MSTRG.12843.10	miR-486b-5p
MSTRG.12843.10	miR-486a-5p
MSTRG.12843.10	miR-1955-3p
MSTRG.12843.10	miR-6984-3p
MSTRG.12843.10	miR-6925-5p
MSTRG.12843.10	miR-5710
MSTRG.12843.10	miR-421-3p
MSTRG.12843.10	let-7f-2-3p
MSTRG.12843.10	miR-3113-5p
MSTRG.12843.10	miR-7650-5p
MSTRG.12843.10	miR-669d-5p
MSTRG.12843.10	miR-7010-5p
MSTRG.12843.10	miR-1264-3p
MSTRG.12843.10	miR-667-5p
MSTRG.12843.10	miR-320-3p
MSTRG.12843.10	miR-130b-5p
MSTRG.12843.10	miR-7667-5p
MSTRG.12843.10	miR-383-5p
MSTRG.12843.10	miR-222-3p
MSTRG.12843.10	miR-221-3p
MSTRG.12843.10	miR-6922-3p
MSTRG.12843.10	let-7e-5p
MSTRG.12843.10	let-7a-5p
MSTRG.12843.10	miR-539-5p
MSTRG.12843.10	miR-185-5p
MSTRG.12843.10	miR-3535
MSTRG.12843.10	miR-8106
MSTRG.12843.10	miR-686
MSTRG.12843.10	miR-504-3p
MSTRG.12843.10	miR-7019-3p
MSTRG.12843.10	miR-6933-3p
MSTRG.12843.10	miR-3474
MSTRG.12843.10	miR-7051-5p
MSTRG.12843.10	miR-1298-5p
MSTRG.12843.10	miR-706
MSTRG.12843.10	miR-5125

MSTRG.12843.10	miR-466n-3p
MSTRG.12843.10	miR-669i
MSTRG.12843.10	miR-299a-5p
MSTRG.12843.10	miR-3100-5p
MSTRG.12843.10	miR-3064-5p
MSTRG.12843.10	miR-208a-5p
MSTRG.12843.10	miR-98-5p
MSTRG.12843.10	let-7i-5p
MSTRG.12843.10	let-7g-5p
MSTRG.12843.10	let-7f-5p
MSTRG.12843.10	let-7d-5p
MSTRG.12843.10	miR-3087-3p
MSTRG.12843.10	miR-329-3p
MSTRG.12843.10	miR-27b-3p
MSTRG.12843.10	miR-195a-3p
MSTRG.12843.10	miR-6998-5p
MSTRG.12843.10	miR-23b-3p
MSTRG.12843.10	miR-23a-3p
MSTRG.12843.10	miR-1943-5p
MSTRG.12843.10	miR-1224-5p
MSTRG.12843.10	miR-701-3p
MSTRG.12843.10	miR-3102-5p.2-5p
MSTRG.12843.10	miR-664-5p
MSTRG.12843.10	miR-7661-3p
MSTRG.12843.10	miR-212-5p
MSTRG.12843.10	miR-6997-5p
MSTRG.12843.10	miR-547-3p
MSTRG.12843.10	miR-7665-5p
MSTRG.12843.10	miR-6976-5p
MSTRG.12843.10	miR-193a-5p
MSTRG.12843.10	miR-433-3p
MSTRG.12843.10	miR-532-5p
MSTRG.12843.10	miR-674-3p
MSTRG.12843.10	miR-181b-2-3p
MSTRG.12843.10	miR-28a-5p
MSTRG.12843.10	miR-1954
MSTRG.12843.10	miR-7213-3p
MSTRG.12843.10	miR-694
MSTRG.12843.10	miR-6983-5p
MSTRG.12843.10	miR-216b-3p
MSTRG.12843.10	miR-6973b-3p
MSTRG.12843.10	miR-431-5p
MSTRG.12843.10	miR-185-3p
MSTRG.12843.10	miR-384-5p

MSTRG.12843.10	miR-30d-5p
MSTRG.12843.10	miR-30c-5p
MSTRG.12843.10	miR-3105-3p
MSTRG.12843.10	miR-683
MSTRG.12843.10	miR-30b-3p
MSTRG.12843.10	miR-669o-5p
MSTRG.12843.10	miR-3060-3p
MSTRG.12843.10	miR-873a-3p
MSTRG.12843.10	miR-302b-3p
MSTRG.12843.10	miR-190b-5p
MSTRG.12843.10	miR-345-3p
MSTRG.12843.10	miR-1187
MSTRG.12843.10	miR-7116-5p
MSTRG.12843.10	miR-7a-2-3p
MSTRG.12843.10	miR-411-3p
MSTRG.12843.10	miR-379-3p
MSTRG.12843.10	miR-760-3p
MSTRG.12843.10	miR-219a-1-3p
MSTRG.12843.10	miR-1941-3p
MSTRG.12843.10	miR-449c-3p
MSTRG.12843.10	miR-511-3p
MSTRG.12843.10	miR-1981-3p
MSTRG.12843.10	miR-410-3p
MSTRG.12843.10	miR-344d-3p
MSTRG.12843.10	miR-3094-3p
MSTRG.16327.2	miR-466k
MSTRG.16327.2	miR-6903-3p
MSTRG.16327.2	miR-28c
MSTRG.16327.2	miR-23b-3p
MSTRG.16327.2	miR-23a-3p
MSTRG.16327.2	miR-1930-3p
MSTRG.16327.2	miR-143-5p
MSTRG.16327.2	miR-5621-5p
MSTRG.16327.2	miR-30f
MSTRG.16327.2	miR-7651-5p
MSTRG.16327.2	miR-5710
MSTRG.16327.2	miR-1964-5p
MSTRG.3640.2	miR-466k
MSTRG.3640.2	miR-5101
MSTRG.3640.2	miR-6903-3p
MSTRG.3640.2	miR-7116-3p
MSTRG.3640.2	miR-466l-3p
MSTRG.3640.2	miR-346-5p
MSTRG.3640.2	miR-3094-3p

MSTRG.3640.2	miR-30e-3p
MSTRG.3640.2	miR-30a-3p
MSTRG.3640.2	miR-5615-5p
MSTRG.3640.2	miR-6946-3p
MSTRG.3640.2	miR-551b-5p
MSTRG.3640.2	miR-694
MSTRG.3640.2	miR-7116-5p
MSTRG.3640.2	miR-335-3p
MSTRG.3640.2	miR-130b-5p
MSTRG.3640.2	miR-7065-3p
MSTRG.3640.2	miR-376b-5p
MSTRG.3640.2	miR-27b-3p
MSTRG.3640.2	miR-6913-3p
MSTRG.3640.2	miR-664-3p
MSTRG.3640.2	miR-494-3p
MSTRG.3640.2	miR-6915-3p
MSTRG.3640.2	miR-692
MSTRG.3640.2	miR-7046-3p
MSTRG.3640.2	miR-3074-5p
MSTRG.3640.2	miR-21b
MSTRG.3640.2	miR-7a-1-3p
MSTRG.3640.2	miR-7026-5p
MSTRG.3640.2	miR-511-3p
MSTRG.3640.2	miR-433-3p
MSTRG.3640.2	miR-145a-3p
MSTRG.3640.2	miR-411-3p
MSTRG.3640.2	miR-379-3p
MSTRG.3640.2	miR-6999-5p
MSTRG.3640.2	miR-3065-5p
MSTRG.3640.2	miR-7054-5p
MSTRG.3640.2	miR-186-5p
MSTRG.3640.2	miR-466n-3p
MSTRG.3640.2	miR-1981-3p
MSTRG.3640.2	miR-412-3p
MSTRG.3640.2	miR-7092-3p
MSTRG.3640.2	miR-6975-3p
MSTRG.3640.2	let-7c-2-3p
MSTRG.3640.2	let-7a-1-3p
MSTRG.3640.2	miR-1983
MSTRG.3640.2	miR-1954
MSTRG.3640.2	miR-7055-5p
MSTRG.3640.2	miR-669f-3p
MSTRG.3640.2	miR-467a-3p
MSTRG.3640.2	miR-194-2-3p

MSTRG.3640.2	miR-1198-5p
MSTRG.3640.2	miR-1264-3p
MSTRG.3640.2	miR-6900-3p
MSTRG.3640.2	miR-466o-3p
MSTRG.3640.2	miR-466m-3p
MSTRG.3640.2	miR-3094-5p
MSTRG.3640.2	miR-1930-3p
MSTRG.3640.2	miR-143-5p
MSTRG.3640.2	miR-6932-5p
MSTRG.3640.2	miR-672-3p
MSTRG.3640.2	miR-1894-5p
MSTRG.3640.2	miR-6769b-3p
MSTRG.3640.2	miR-8118
MSTRG.3640.2	miR-676-5p
MSTRG.3640.2	miR-223-5p
MSTRG.3640.2	miR-219c-3p
MSTRG.3640.2	miR-29b-2-5p
MSTRG.3640.2	miR-1194
MSTRG.3640.2	miR-686
MSTRG.3640.2	miR-382-3p
MSTRG.3640.2	miR-344h-5p
MSTRG.3640.2	miR-344e-5p
MSTRG.3640.2	miR-6944-3p
MSTRG.3640.2	miR-3470a
MSTRG.3640.2	miR-6925-5p
MSTRG.3640.2	miR-6240
MSTRG.3640.2	miR-504-3p
MSTRG.3640.2	miR-330-3p
MSTRG.3640.2	miR-466e-3p
MSTRG.3640.2	miR-466d-3p
MSTRG.3640.2	miR-466a-3p
MSTRG.3640.2	miR-539-5p
MSTRG.3640.2	miR-409-3p
MSTRG.3640.2	miR-6901-3p
MSTRG.3640.2	miR-361-5p
MSTRG.3640.2	miR-532-3p
MSTRG.3640.2	miR-466p-5p
MSTRG.3640.2	miR-466a-5p
MSTRG.3640.2	miR-1187
MSTRG.3640.2	miR-7013-5p
MSTRG.3640.2	miR-3092-3p
MSTRG.3640.2	miR-6516-3p
MSTRG.3640.2	miR-3062-5p
MSTRG.3640.2	miR-129-5p

MSTRG.3640.2	miR-712-5p
MSTRG.3640.2	miR-128-3p
MSTRG.3640.2	miR-7667-3p
MSTRG.3640.2	miR-30c-2-3p
MSTRG.3640.2	miR-30c-1-3p
MSTRG.3640.2	miR-322-3p
MSTRG.3640.2	miR-466e-5p
MSTRG.3640.2	miR-222-5p
MSTRG.3640.2	miR-7685-3p
MSTRG.3640.2	miR-410-3p
MSTRG.3640.2	miR-344d-3p
MSTRG.3640.2	miR-3066-3p
MSTRG.3640.2	miR-873a-5p
MSTRG.3640.2	miR-7220-3p
MSTRG.3640.2	miR-467e-3p
MSTRG.3640.2	miR-467d-3p
MSTRG.3640.2	miR-467c-3p
MSTRG.3640.2	miR-467b-3p
MSTRG.3640.2	miR-3475-5p
MSTRG.3640.2	miR-216b-3p
MSTRG.3640.2	miR-9-3p
MSTRG.3640.2	miR-153-5p
MSTRG.3640.2	miR-8106
MSTRG.3640.2	miR-3535
MSTRG.3640.2	miR-6916-5p
MSTRG.3640.2	miR-669p-3p
MSTRG.3640.2	miR-135b-3p
MSTRG.3640.2	miR-543-3p
MSTRG.3640.2	miR-185-5p
MSTRG.3640.2	miR-6927-3p
MSTRG.3640.2	miR-195a-3p
MSTRG.3640.2	miR-6540-5p
MSTRG.3640.2	miR-7027-5p
MSTRG.3640.2	miR-1224-5p
MSTRG.3640.2	miR-466i-3p
MSTRG.3640.2	miR-501-5p
MSTRG.3640.2	miR-203-3p
MSTRG.3640.2	miR-3082-5p
MSTRG.3640.2	miR-6908-3p
MSTRG.3640.2	miR-7010-5p
MSTRG.3640.2	miR-466o-5p
MSTRG.3640.2	miR-466c-5p
MSTRG.3640.2	miR-466b-5p
MSTRG.3640.2	miR-503-3p

MSTRG.3640.2	miR-1941-3p
MSTRG.3640.2	miR-706
MSTRG.3640.2	miR-26b-3p
MSTRG.3640.2	miR-26a-2-3p
MSTRG.3640.2	miR-6998-5p
MSTRG.3640.2	miR-3102-3p.2-3p
MSTRG.3640.2	miR-450b-3p
MSTRG.3640.2	miR-450a-1-3p
MSTRG.3640.2	miR-6912-3p
MSTRG.3640.2	miR-668-3p
MSTRG.3640.2	miR-222-3p
MSTRG.3640.2	miR-221-3p
MSTRG.3640.2	miR-6902-3p
MSTRG.3640.2	miR-6911-3p
MSTRG.3640.2	miR-7231-5p
MSTRG.3640.2	miR-505-5p
MSTRG.3640.2	miR-1949
MSTRG.3640.2	miR-7087-5p
MSTRG.3640.2	miR-7224-3p
MSTRG.3640.2	miR-669i
MSTRG.3640.2	miR-547-3p
MSTRG.3640.2	miR-126a-5p
MSTRG.3640.2	miR-7055-3p
MSTRG.3640.2	miR-6955-3p
MSTRG.3640.2	miR-3100-5p
MSTRG.3640.2	miR-7240-5p
MSTRG.3640.2	miR-6947-5p
MSTRG.3640.2	miR-7665-5p
MSTRG.3640.2	miR-6976-5p
MSTRG.3640.2	miR-30f
MSTRG.3640.2	miR-384-5p
MSTRG.3640.2	miR-30d-5p
MSTRG.3640.2	miR-30c-5p
MSTRG.3640.2	miR-181b-2-3p
MSTRG.3640.2	miR-669d-5p
MSTRG.3640.2	miR-3095-3p
MSTRG.3640.2	miR-669a-3-3p
MSTRG.3640.2	miR-499-3p
MSTRG.3640.2	miR-7077-5p
MSTRG.3640.2	miR-5125
MSTRG.3640.2	miR-542-3p
MSTRG.3640.2	miR-679-3p
MSTRG.3640.2	miR-144-5p
MSTRG.3640.2	miR-7647-3p

MSTRG.3640.2	miR-7031-5p
MSTRG.3640.2	miR-582-3p
MSTRG.3640.2	miR-493-5p
MSTRG.3640.2	miR-7061-5p
MSTRG.3640.2	miR-466q
MSTRG.3640.2	miR-3087-3p
MSTRG.3640.2	miR-3078-5p
MSTRG.3640.2	miR-301a-5p
MSTRG.3640.2	miR-7081-3p
MSTRG.3640.2	miR-204-5p
MSTRG.3640.2	miR-190b-3p
MSTRG.3640.2	miR-495-3p
MSTRG.3640.2	miR-351-5p
MSTRG.3640.2	miR-125b-5p
MSTRG.3640.2	miR-125a-5p
MSTRG.3877.7	miR-466k
MSTRG.3877.7	miR-7116-3p
MSTRG.3877.7	miR-6903-3p
MSTRG.3877.7	miR-5101
MSTRG.3877.7	miR-126a-5p
MSTRG.3877.7	miR-153-5p
MSTRG.3877.7	miR-7240-5p
MSTRG.3877.7	miR-485-3p
MSTRG.3877.7	miR-551b-5p
MSTRG.3877.7	miR-505-3p
MSTRG.3877.7	miR-5125
MSTRG.3877.7	miR-351-3p
MSTRG.3877.7	miR-3475-5p
MSTRG.3877.7	miR-335-3p
MSTRG.3877.7	miR-302b-3p
MSTRG.3877.7	miR-7077-5p
MSTRG.3877.7	miR-3095-5p
MSTRG.3877.7	miR-6983-5p
MSTRG.3877.7	miR-7026-5p
MSTRG.3877.7	miR-23b-3p
MSTRG.3877.7	miR-23a-3p
MSTRG.3877.7	miR-129-5p
MSTRG.3877.7	miR-6946-3p
MSTRG.3877.7	miR-190b-3p
MSTRG.3877.7	miR-6516-5p
MSTRG.3877.7	miR-7220-5p
MSTRG.3877.7	miR-7073-5p
MSTRG.3877.7	miR-181b-2-3p
MSTRG.3877.7	miR-702-3p

MSTRG.3877.7	miR-203-3p
MSTRG.3877.7	miR-7010-5p
MSTRG.3877.7	miR-694
MSTRG.3877.7	miR-335-5p
MSTRG.3877.7	miR-185-5p
MSTRG.3877.7	miR-421-3p
MSTRG.3877.7	miR-467c-5p
MSTRG.3877.7	miR-328-3p
MSTRG.3877.7	miR-361-5p
MSTRG.3877.7	miR-450b-5p
MSTRG.3877.7	miR-3092-3p
MSTRG.3877.7	miR-6240
MSTRG.3877.7	miR-330-3p
MSTRG.3877.7	miR-706
MSTRG.3877.7	miR-5621-5p
MSTRG.14838.1	miR-3084-3p
MSTRG.14838.1	miR-377-5p
MSTRG.14838.1	miR-153-5p
MSTRG.14838.1	miR-7671-5p
MSTRG.1243.32	miR-5101
MSTRG.1243.32	miR-466o-3p
MSTRG.1243.32	miR-466m-3p
MSTRG.1243.32	miR-706
MSTRG.1243.32	miR-466e-3p
MSTRG.1243.32	miR-466d-3p
MSTRG.1243.32	miR-466a-3p
MSTRG.1243.32	miR-7092-3p
MSTRG.1243.32	miR-222-3p
MSTRG.1243.32	miR-221-3p
MSTRG.1243.32	miR-6922-3p
MSTRG.1243.32	miR-466p-3p
MSTRG.1243.32	miR-466c-3p
MSTRG.1243.32	miR-466b-3p
MSTRG.1243.32	miR-7054-5p
MSTRG.1243.32	miR-484
MSTRG.1243.32	miR-7010-5p
MSTRG.1243.32	miR-181b-5p
MSTRG.1243.32	miR-669f-3p
MSTRG.1243.32	miR-467a-3p
MSTRG.1243.32	miR-1264-3p
MSTRG.1243.32	miR-5626-3p
MSTRG.1243.32	miR-181b-2-3p
MSTRG.1243.32	miR-6402
MSTRG.1243.32	miR-7052-3p

MSTRG.1243.32	miR-6996-5p
MSTRG.1243.32	miR-30e-3p
MSTRG.1243.32	miR-30a-3p
MSTRG.1243.32	miR-669c-3p
MSTRG.1243.32	miR-6916-5p
MSTRG.1243.32	miR-7027-5p
MSTRG.1243.32	miR-329-3p
MSTRG.1243.32	miR-466f-3p
MSTRG.1243.32	miR-6946-3p
MSTRG.1243.32	miR-6999-5p
MSTRG.1243.32	miR-504-3p
MSTRG.1243.32	miR-6958-3p
MSTRG.1243.32	miR-3100-5p
MSTRG.1243.32	miR-30c-2-3p
MSTRG.1243.32	miR-30c-1-3p
MSTRG.1243.32	miR-676-5p
MSTRG.1243.32	miR-7073-5p
MSTRG.1243.32	miR-18b-5p
MSTRG.1243.32	miR-7116-3p
MSTRG.1243.32	miR-7b-5p
MSTRG.1243.32	miR-7a-5p
MSTRG.1243.32	miR-1194
MSTRG.1243.32	miR-7051-5p
MSTRG.1243.32	miR-467e-3p
MSTRG.1243.32	miR-467d-3p
MSTRG.1243.32	miR-467c-3p
MSTRG.1243.32	miR-467b-3p
MSTRG.1243.32	miR-466n-3p
MSTRG.1243.32	miR-669i
MSTRG.1243.32	miR-335-3p
MSTRG.1243.32	miR-6902-3p
MSTRG.1243.32	miR-760-5p
MSTRG.1243.32	miR-501-5p
MSTRG.1243.32	miR-692
MSTRG.1243.32	miR-7661-3p
MSTRG.1243.32	miR-466i-3p
MSTRG.1243.32	miR-6769b-3p
MSTRG.1243.32	miR-128-3p
MSTRG.1243.32	miR-664-3p
MSTRG.1243.32	miR-6983-5p
MSTRG.1243.32	miR-551b-5p
MSTRG.1243.32	miR-6911-5p
MSTRG.1243.32	miR-7055-5p
MSTRG.1243.32	miR-874-3p

MSTRG.1243.32	miR-3572-3p
MSTRG.1243.32	miR-412-3p
MSTRG.1243.32	miR-378a-5p
MSTRG.1243.32	miR-7a-2-3p
MSTRG.1243.32	miR-148b-3p
MSTRG.1243.32	miR-3110-3p
MSTRG.1243.32	miR-7668-5p
MSTRG.1243.32	miR-1983
MSTRG.1243.32	miR-7081-3p
MSTRG.1243.32	miR-219a-1-3p
MSTRG.1243.32	miR-194-2-3p
MSTRG.1243.32	miR-6984-3p
MSTRG.1243.32	miR-5129-5p
MSTRG.1243.32	miR-877-3p
MSTRG.1243.32	miR-5124b
MSTRG.1243.32	miR-222-5p
MSTRG.1243.32	miR-365-3p
MSTRG.1243.32	miR-1981-3p
MSTRG.1243.32	miR-129b-5p
MSTRG.1243.32	miR-701-3p
MSTRG.1243.32	miR-511-3p
MSTRG.1243.32	miR-7068-3p
MSTRG.1243.32	miR-539-5p
MSTRG.1243.32	miR-129b-3p
MSTRG.1243.32	miR-483-3p
MSTRG.1243.32	miR-450b-3p
MSTRG.1243.32	miR-450a-1-3p
MSTRG.1243.32	miR-3082-3p
MSTRG.1243.32	miR-186-5p
MSTRG.1243.32	let-7f-2-3p
MSTRG.1243.32	miR-6541
MSTRG.1243.32	miR-6240
MSTRG.1243.32	miR-770-3p
MSTRG.1243.32	miR-6991-5p
MSTRG.1243.32	miR-1668
MSTRG.1243.32	miR-345-3p
MSTRG.1243.32	miR-139-5p
MSTRG.1243.32	miR-24-3p
MSTRG.1243.32	miR-7651-5p
MSTRG.1243.32	miR-330-3p
MSTRG.1243.32	miR-7665-5p
MSTRG.5009.1	miR-28a-3p
MSTRG.5009.1	miR-138-2-3p
MSTRG.5009.1	miR-3097-3p

MSTRG.5009.1	miR-3082-5p

Table S3. Information of the downregulated lncRNA-miRNA network in the cortex of 7-month-old 5×FAD mice.

Downregulated lncRNA	miRNA targets
ENSMUST00000184170	miR-380-3p
ENSMUST00000184170	miR-1933-3p
ENSMUST00000184170	miR-743b-3p
ENSMUST00000184170	miR-669b-5p
ENSMUST00000184170	miR-214-3p
ENSMUST00000184170	miR-7080-3p
ENSMUST00000184170	miR-411-5p
ENSMUST00000184170	miR-153-3p
ENSMUST00000184170	miR-15a-5p
ENSMUST00000184170	miR-16-5p
ENSMUST00000184170	miR-322-5p
ENSMUST00000184170	miR-497a-5p
ENSMUST00000184170	miR-195b
ENSMUST00000184170	miR-193a-3p
ENSMUST00000184170	miR-193b-3p
ENSMUST00000184170	miR-18a-5p
ENSMUST00000184170	miR-3070-5p
ENSMUST00000184170	miR-488-3p
ENSMUST00000184170	miR-145b
ENSMUST00000184170	miR-127-5p
ENSMUST00000184170	miR-7043-3p
ENSMUST00000184170	miR-669p-5p
ENSMUST00000184170	miR-29c-5p
ENSMUST00000184170	miR-3058-3p
ENSMUST00000145774	miR-7056-5p
ENSMUST00000132392	miR-23b-5p
ENSMUST00000132392	miR-7048-3p
ENSMUST00000132392	miR-98-3p
ENSMUST00000132392	let-7f-1-3p
ENSMUST00000132392	let-7b-3p
ENSMUST00000132392	miR-7077-3p
ENSMUST00000182451	miR-669e-5p
ENSMUST00000182451	miR-1933-3p
ENSMUST00000182451	miR-6906-5p
ENSMUST00000182451	miR-666-3p
ENSMUST00000161415	miR-6919-5p
ENSMUST00000161415	miR-6954-3p
ENSMUST00000161415	miR-574-5p
ENSMUST00000161415	miR-1291
ENSMUST00000161415	miR-1258-3p
ENSMUST00000161415	miR-210-5p

ENSMUST00000161415	miR-709
ENSMUST00000161415	miR-342-5p
ENSMUST00000161415	miR-7684-5p
ENSMUST00000161415	miR-3078-3p
ENSMUST00000161415	miR-3087-5p
ENSMUST00000161415	miR-7034-3p
ENSMUST00000161415	miR-3084-5p
ENSMUST00000161415	miR-3090-3p
ENSMUST00000161415	miR-134-5p
ENSMUST00000161415	miR-330-5p
ENSMUST00000161415	miR-326-3p
ENSMUST00000161415	miR-6907-5p
ENSMUST00000161415	miR-101b-3p
ENSMUST00000161415	miR-1188-3p
ENSMUST00000161415	miR-669e-5p
ENSMUST00000161637	miR-346-3p
ENSMUST00000161637	miR-210-5p
ENSMUST00000161637	miR-27a-3p
ENSMUST00000161637	miR-193b-5p
ENSMUST00000161637	miR-300-5p
ENSMUST00000161637	miR-16-1-3p
ENSMUST00000161637	miR-1258-5p
ENSMUST00000161637	miR-16-2-3p
ENSMUST00000161637	miR-219a-5p
ENSMUST00000161637	miR-702-5p
ENSMUST00000161637	miR-337-3p
ENSMUST00000161637	miR-7013-3p
ENSMUST00000161637	miR-881-3p
ENSMUST00000161637	miR-300-3p
ENSMUST00000161637	miR-3078-3p
ENSMUST00000161637	miR-6985-3p
ENSMUST00000161637	miR-673-5p
ENSMUST00000161637	miR-6900-5p
ENSMUST00000161637	miR-666-3p
ENSMUST00000161637	miR-152-5p
ENSMUST00000161637	miR-376c-3p
ENSMUST00000161637	miR-214-5p
ENSMUST00000124691	miR-3098-3p
ENSMUST00000124691	miR-6899-3p
ENSMUST00000124691	miR-28b
ENSMUST00000124691	miR-136-5p
ENSMUST00000141539	miR-6918-5p
ENSMUST00000141539	miR-7037-3p
ENSMUST00000141539	miR-1224-3p

ENSMUST00000141539	miR-3067-5p
ENSMUST00000141539	miR-216a-3p
ENSMUST00000141539	miR-214-3p
ENSMUST00000141539	miR-6954-3p
ENSMUST00000141539	miR-1258-5p
ENSMUST00000162401	miR-6901-5p
ENSMUST00000162401	miR-20a-3p
ENSMUST00000162401	miR-326-3p
ENSMUST00000162401	miR-330-5p
ENSMUST00000162401	miR-337-3p
ENSMUST00000162401	miR-3085-5p
ENSMUST00000162401	miR-7662-5p
ENSMUST00000162401	miR-152-3p
ENSMUST00000162401	miR-148a-3p
ENSMUST00000162401	miR-8103
ENSMUST00000162401	miR-7675-3p
ENSMUST00000162401	miR-214-3p
ENSMUST00000162401	miR-690
ENSMUST00000139277	miR-30d-3p
ENSMUST00000139277	miR-6934-5p
ENSMUST00000139277	miR-211-5p
ENSMUST00000139277	miR-3088-3p
ENSMUST00000129857	miR-378b
ENSMUST00000129857	miR-7043-3p
ENSMUST00000129857	miR-6418-3p
ENSMUST00000129857	miR-666-3p
ENSMUST00000129857	miR-3057-5p
ENSMUST00000129857	miR-7034-3p
ENSMUST00000129857	miR-935
ENSMUST00000129857	miR-7117-3p
ENSMUST00000129857	miR-145b
ENSMUST00000129857	miR-6966-3p
ENSMUST00000129857	miR-5624-5p
ENSMUST00000129857	miR-138-5p
ENSMUST00000129857	miR-7237-3p
ENSMUST00000129857	miR-23b-5p
ENSMUST00000129857	miR-3078-3p
ENSMUST00000129857	miR-337-3p
ENSMUST00000129857	miR-669k-5p
ENSMUST00000209301	miR-7222-3p
ENSMUST00000199237	miR-871-3p
ENSMUST00000199237	miR-700-5p
ENSMUST00000199237	miR-758-3p
ENSMUST00000199237	miR-666-3p

ENSMUST00000199237	let-7k
ENSMUST00000199237	let-7c-5p
ENSMUST00000199237	let-7b-5p
ENSMUST00000199237	miR-7024-3p
ENSMUST00000137980	miR-296-5p
ENSMUST00000137980	miR-490-3p
ENSMUST00000137980	miR-107-5p
ENSMUST00000137980	miR-103-2-5p
ENSMUST00000137980	miR-103-1-5p
ENSMUST00000137980	miR-1958
ENSMUST00000137980	miR-5621-3p
ENSMUST00000141357	miR-6898-5p
ENSMUST00000141357	miR-339-5p
ENSMUST00000141357	miR-7211-5p
ENSMUST00000141357	miR-743b-3p
ENSMUST00000141357	miR-708-5p
ENSMUST00000141357	miR-337-3p
ENSMUST00000141357	miR-135b-5p
ENSMUST00000141357	miR-135a-5p
ENSMUST00000141357	miR-7049-5p
ENSMUST00000141357	miR-6979-3p
ENSMUST00000210394	miR-298-5p
ENSMUST00000210394	miR-6900-5p
ENSMUST00000210394	miR-3073a-3p
ENSMUST00000146588	miR-182-5p
ENSMUST00000146588	miR-7000-3p
ENSMUST00000146588	miR-876-5p
ENSMUST00000146588	miR-1249-5p
ENSMUST00000144628	miR-7042-5p
ENSMUST00000144628	miR-5615-3p
ENSMUST00000144628	miR-8107
ENSMUST00000144628	miR-3086-5p
ENSMUST00000144628	miR-7243-3p
ENSMUST00000144628	miR-214-5p
ENSMUST00000144628	miR-6913-5p
ENSMUST00000144628	miR-376b-3p
ENSMUST00000145741	miR-7214-5p
ENSMUST00000145741	miR-290b-5p
ENSMUST00000145741	miR-142b
ENSMUST00000145741	miR-199b-5p
ENSMUST00000145741	miR-199a-5p
ENSMUST00000145741	miR-22-5p
ENSMUST00000145741	miR-3572-5p
ENSMUST00000145741	miR-1930-5p

ENSMUST00000145741	miR-20a-3p
ENSMUST00000145741	miR-7688-5p
ENSMUST00000145741	miR-7675-3p
ENSMUST00000145741	miR-7024-3p
ENSMUST00000145741	miR-96-3p
ENSMUST00000145741	miR-344f-5p
ENSMUST00000145741	miR-7669-3p
ENSMUST00000145741	miR-449a-5p
ENSMUST00000145741	miR-34a-5p
ENSMUST00000145741	miR-3088-3p
ENSMUST00000145741	miR-1231-5p
ENSMUST00000145741	miR-743b-3p
ENSMUST00000145741	miR-224-5p
ENSMUST00000145741	miR-6905-5p
ENSMUST00000145741	miR-6994-5p
ENSMUST00000145741	miR-3106-5p
ENSMUST00000145741	miR-206-3p
ENSMUST00000145741	miR-767
ENSMUST00000145741	miR-449c-5p
ENSMUST00000145741	miR-449b
ENSMUST00000145741	miR-34c-5p
ENSMUST00000145741	miR-34b-5p
ENSMUST00000145741	miR-130b-3p
ENSMUST00000145741	miR-7034-3p
ENSMUST00000145741	miR-19b-3p
ENSMUST00000145741	miR-19a-3p
ENSMUST00000145741	miR-300-5p
ENSMUST00000145741	miR-7048-3p
ENSMUST00000145741	miR-135b-5p
ENSMUST00000145741	miR-135a-5p
ENSMUST00000221246	miR-7117-3p
ENSMUST00000221246	miR-300-5p
ENSMUST00000221246	miR-665-3p
ENSMUST00000221246	miR-744-3p
ENSMUST00000221246	miR-5619-5p
ENSMUST00000221246	miR-290b-5p
ENSMUST00000221246	miR-6967-3p
ENSMUST00000221246	miR-6896-3p
ENSMUST00000221246	miR-205-5p
ENSMUST00000221246	miR-6918-5p
ENSMUST00000221246	miR-147-5p
MSTRG.17500.1	miR-466i-5p
MSTRG.17500.1	miR-466d-5p
MSTRG.17500.1	miR-6899-3p

MSTRG.17500.1	miR-3090-3p
MSTRG.17500.1	miR-210-5p
MSTRG.17500.1	miR-6940-3p
MSTRG.17500.1	miR-7675-3p
MSTRG.17500.1	miR-6951-5p
MSTRG.17500.1	miR-3473a
MSTRG.17500.1	miR-20b-3p
MSTRG.17500.1	miR-17-3p
MSTRG.17500.1	miR-3073a-3p
MSTRG.17500.1	miR-199b-3p
MSTRG.17500.1	miR-199a-3p
MSTRG.17500.1	miR-134-5p
MSTRG.17500.1	miR-6979-3p
MSTRG.17500.1	miR-7056-5p
MSTRG.17500.1	miR-340-5p
MSTRG.17500.1	miR-7000-3p
MSTRG.17500.1	miR-421-5p
MSTRG.17500.1	miR-670-3p
MSTRG.17500.1	miR-3110-5p
MSTRG.17500.1	miR-1258-5p
MSTRG.17500.1	miR-3547-5p
MSTRG.17500.1	miR-1249-3p
MSTRG.17500.1	miR-491-3p
MSTRG.17500.1	miR-3098-3p
MSTRG.17500.1	miR-7217-5p
MSTRG.17500.1	miR-6953-3p
MSTRG.17500.1	miR-7117-3p
MSTRG.17500.1	miR-211-5p
MSTRG.17500.1	miR-6537-5p
MSTRG.17500.1	miR-202-5p
MSTRG.17500.1	miR-134-3p
MSTRG.17500.1	miR-3059-5p
MSTRG.18699.11	miR-216a-3p
MSTRG.18699.11	miR-187-5p
MSTRG.18699.11	miR-3108-5p
MSTRG.18699.11	miR-1231-3p
MSTRG.18699.11	miR-652-5p
MSTRG.18699.11	miR-6907-5p
MSTRG.18699.11	miR-6900-5p
MSTRG.18699.11	miR-6967-3p
MSTRG.7748.1	miR-466i-5p
MSTRG.7748.1	miR-466d-5p
MSTRG.7748.1	miR-7217-5p
MSTRG.7748.1	miR-297a-5p

MSTRG.7748.1	miR-297c-5p
MSTRG.7748.1	miR-297b-5p
MSTRG.7748.1	miR-6896-3p
MSTRG.7748.1	miR-7118-3p
MSTRG.7748.1	miR-669b-5p
MSTRG.7748.1	miR-1a-1-5p
MSTRG.7748.1	miR-802-5p
MSTRG.7748.1	miR-7093-3p
MSTRG.7748.1	miR-19b-1-5p
MSTRG.7748.1	miR-190a-3p
MSTRG.7748.1	miR-488-3p
MSTRG.7748.1	miR-466n-5p
MSTRG.7748.1	miR-3064-3p
MSTRG.7748.1	miR-758-3p
MSTRG.7748.1	miR-374b-5p
MSTRG.7748.1	miR-3963
MSTRG.7748.1	miR-3086-5p
MSTRG.7748.1	miR-7047-3p
MSTRG.7748.1	miR-134-5p
MSTRG.7748.1	miR-669k-5p
MSTRG.7748.1	miR-1192
MSTRG.7748.1	miR-6952-3p
MSTRG.7748.1	miR-3088-3p
MSTRG.7748.1	miR-876-5p
MSTRG.7748.1	miR-7043-3p
MSTRG.7748.1	miR-200a-3p
MSTRG.7748.1	miR-141-3p
MSTRG.7748.1	miR-6896-5p
MSTRG.7748.1	miR-5107-3p
MSTRG.7748.1	miR-7043-5p
MSTRG.7748.1	miR-27a-3p
MSTRG.7748.1	miR-496a-3p
MSTRG.7748.1	miR-1929-5p
MSTRG.7748.1	miR-489-3p
MSTRG.7748.1	miR-122-5p
MSTRG.7748.1	miR-146a-3p
MSTRG.7748.1	miR-3964
MSTRG.7748.1	miR-202-5p
MSTRG.7748.1	miR-653-5p
MSTRG.7748.1	miR-337-3p
MSTRG.7748.1	miR-183-3p
MSTRG.7748.1	miR-130a-5p
MSTRG.7748.1	miR-301b-5p
MSTRG.7748.1	miR-142b

MSTRG.7748.1	miR-1968-5p
MSTRG.7748.1	miR-669b-3p
MSTRG.7748.1	miR-1b-5p
MSTRG.7748.1	miR-880-3p
MSTRG.7748.1	miR-470-5p
MSTRG.7748.1	miR-547-5p
MSTRG.7748.1	miR-124-5p
MSTRG.7748.1	miR-7048-3p
MSTRG.7748.1	miR-7037-3p
MSTRG.7748.1	miR-7015-5p
MSTRG.7748.1	miR-421-5p
MSTRG.7748.1	miR-6481
MSTRG.7748.1	miR-1231-3p
MSTRG.7748.1	miR-30e-5p
MSTRG.7748.1	miR-30b-5p
MSTRG.7748.1	miR-30a-5p
MSTRG.7748.1	miR-136-5p
MSTRG.7748.1	miR-1930-5p
MSTRG.7748.1	miR-7077-3p
MSTRG.7748.1	let-7c-1-3p
MSTRG.7748.1	miR-5620-3p
MSTRG.7748.1	miR-6537-5p
MSTRG.7748.1	miR-20a-3p
MSTRG.7748.1	miR-669m-5p
MSTRG.7748.1	miR-466m-5p
MSTRG.7748.1	miR-466j
MSTRG.7748.1	miR-466h-5p
MSTRG.7748.1	miR-9-5p
MSTRG.7748.1	miR-669d-3p
MSTRG.7748.1	miR-297c-3p
MSTRG.7748.1	miR-297b-3p
MSTRG.7748.1	miR-297a-3p
MSTRG.7748.1	miR-217-5p
MSTRG.7748.1	miR-290a-5p
MSTRG.7748.1	miR-1197-3p
MSTRG.7748.1	miR-5121
MSTRG.7748.1	miR-290b-5p
MSTRG.7748.1	miR-592-5p
MSTRG.7748.1	miR-344e-3p
MSTRG.7748.1	miR-3969
MSTRG.7748.1	miR-6942-5p
MSTRG.7748.1	miR-883a-3p
MSTRG.7748.1	miR-154-3p
MSTRG.7748.1	miR-7646-5p

MSTRG.7748.1	miR-6985-5p
MSTRG.7748.1	miR-339-5p
MSTRG.7748.1	miR-679-5p
MSTRG.7748.1	miR-6987-3p
MSTRG.7748.1	miR-669m-3p
MSTRG.7748.1	miR-7056-5p
MSTRG.7748.1	miR-743b-3p
MSTRG.7748.1	miR-669h-5p
MSTRG.7748.1	miR-1936
MSTRG.7748.1	miR-22-5p
MSTRG.7748.1	miR-6912-5p
MSTRG.7748.1	miR-6986-5p
MSTRG.7748.1	miR-6925-3p
MSTRG.7748.1	miR-6951-5p
MSTRG.7748.1	miR-7649-3p
MSTRG.7748.1	miR-7237-3p
MSTRG.7748.1	miR-218-5p
MSTRG.7748.1	miR-324-5p
MSTRG.7748.1	miR-3074-1-3p
MSTRG.7748.1	miR-350-3p
MSTRG.7748.1	miR-8107
MSTRG.7748.1	miR-155-3p
MSTRG.7748.1	miR-6934-3p
MSTRG.7748.1	miR-3473a
MSTRG.7748.1	miR-6954-3p
MSTRG.7748.1	miR-340-5p
MSTRG.7748.1	miR-96-5p
MSTRG.7748.1	miR-3471
MSTRG.7748.1	miR-194-1-3p
MSTRG.7748.1	miR-6989-5p
MSTRG.18699.5	miR-216a-3p
MSTRG.18699.5	miR-187-5p
MSTRG.18699.5	miR-3108-5p
MSTRG.18699.5	miR-669m-5p
MSTRG.18699.5	miR-466m-5p
MSTRG.18699.5	miR-466j
MSTRG.18699.5	miR-466h-5p
MSTRG.18699.5	miR-340-5p
MSTRG.18699.5	miR-7674-3p
MSTRG.18699.5	miR-6481
MSTRG.18699.5	miR-6967-3p
MSTRG.18699.5	miR-466n-5p
MSTRG.18699.5	miR-1258-3p
MSTRG.18699.5	miR-376c-3p

MSTRG.18699.5	miR-466f
MSTRG.18699.5	miR-544-3p
MSTRG.18699.5	miR-7661-5p
MSTRG.18699.5	miR-5126
MSTRG.18699.5	miR-709
MSTRG.18699.5	miR-6945-3p
MSTRG.18699.5	miR-6952-3p
MSTRG.18699.5	miR-155-3p
MSTRG.18699.5	miR-350-3p
MSTRG.18699.5	miR-6900-5p
MSTRG.18699.5	miR-301b-5p
MSTRG.18699.5	miR-744-5p
MSTRG.18699.5	miR-466i-5p
MSTRG.18699.5	miR-703
MSTRG.18699.5	miR-10b-5p
MSTRG.18699.5	miR-10a-5p
MSTRG.18699.5	miR-696
MSTRG.18699.5	miR-19b-3p
MSTRG.18699.5	miR-19a-3p
MSTRG.18699.5	miR-33-3p
MSTRG.18699.5	miR-7042-5p
MSTRG.18699.5	miR-339-5p
MSTRG.18699.5	miR-377-3p
MSTRG.18699.5	miR-338-3p
MSTRG.18699.5	miR-7049-5p
MSTRG.18699.5	miR-7222-3p
MSTRG.18699.5	miR-3473a
MSTRG.18699.5	miR-491-3p
MSTRG.18699.5	miR-1231-3p
MSTRG.18699.5	miR-3089-3p
MSTRG.18699.5	miR-429-3p
MSTRG.18699.5	miR-200c-3p
MSTRG.18699.5	miR-200b-3p
MSTRG.8598.3	miR-188-3p
MSTRG.8598.3	miR-3572-5p
MSTRG.8598.3	miR-3552
MSTRG.8598.3	miR-3066-5p
MSTRG.8598.3	miR-142a-5p
MSTRG.8598.3	miR-19b-3p
MSTRG.8598.3	miR-19a-3p
MSTRG.8598.3	miR-154-3p
MSTRG.8598.3	miR-216a-5p
MSTRG.8598.3	miR-27b-5p
MSTRG.8598.3	miR-141-5p

MSTRG.8598.3	miR-6989-3p
MSTRG.8598.3	miR-670-3p
MSTRG.8598.3	miR-28b
MSTRG.8598.3	miR-20a-5p
MSTRG.8598.3	miR-106b-5p
MSTRG.8598.3	miR-93-5p
MSTRG.8598.3	miR-17-5p
MSTRG.8598.3	miR-106a-5p
MSTRG.8598.3	miR-217-5p
MSTRG.8598.3	miR-6395
MSTRG.8598.3	miR-30e-5p
MSTRG.8598.3	miR-30b-5p
MSTRG.8598.3	miR-30a-5p
MSTRG.8598.3	miR-6896-3p
MSTRG.8598.3	miR-1231-3p
MSTRG.8598.3	miR-324-5p

Table S4. Correlated information of the upregulated lncRNA-associated ceRNA network in the cerebral cortex of 5×FAD mice.

Upregulated lncRNA	Downregulated miRNA	Upregulated mRNA
ENSMUST00000127786	miR-694, miR-329-3p, miR-5101, miR-669f-3p, miR-686, miR-466a-3p, miR-466d-3p, miR-466e-3p, miR-466n-3p, miR-335-3p, miR-466l-3p	Pex19, AW554918, Rnf24, P2ry13, Epha4, Arnt2, Lpl, Dzank1, Cct7, Prkcd, Clec7a, 5730455P16Rik, Tbcel, Rnf165, Brd8, Plek, Eif2s2, Daam1, Tmem175, Dcun1d1, Rin2, Zfp260, Zmym5, Hecw2, R3hdm2, Mgll, Lin54, Gpr34
MSTRG.10455.14	miR-694, miR-466k, miR-669f-3p, miR-686, miR-466a-3p, miR-466d-3p, miR-466e-3p, miR-466n-3p, miR-335-3p, miR-466l-3p	Pex19, AW554918, Rnf24, P2ry13, Epha4, Arnt2, Lpl, Dzank1, Clcn3, Prkcd, Tmem175, Wscd1, Cdc42ep4, Clec7a, Mgll, Rin2, 5730455P16Rik, Zfp260, Zmym5, Rnf165, Plek, Hecw2, Daam1, R3hdm2, Lin54, Gpr34
MSTRG.15393.1	miR-694, miR-466l-3p	Pex19, AW554918, Rnf24, P2ry13, Epha4, Arnt2, Lpl, Lin54
MSTRG.17500.2	miR-694, miR-329-3p, miR-466k, miR-5101, miR-686, miR-466a-3p, miR-466d-3p, miR-466e-3p, miR-466n-3p, miR-466l-3	Pex19, AW554918, Rnf24, P2ry13, Epha4, Arnt2, Lpl, Dzank1, Cct7, Prkcd, Clec7a, Clcn3, Tmem175, Wscd1, Cdc42ep4, Mgll, Rin2, 5730455P16Rik, Tbcel, Rnf165, Brd8, Plek, Eif2s2, Daam1, Dcun1d1, R3hdm2, Zmym5, Lin54, Hecw2
MSTRG.12843.10	miR-694, miR-329-3p, miR-466k, miR-5101, miR-669f-3p, miR-686, miR-466a-3p, miR-466d-3p, miR-466e-3p, miR-466n-3p, miR-335-3p, miR-466l-3p	Pex19, AW554918, Rnf24, P2ry13, Epha4, Arnt2, Lpl, Dzank1, Cct7, Prkcd, Clec7a, Clcn3, Tmem175, Wscd1, Cdc42ep4, Mgll, Rin2, 5730455P16Rik, Tbcel, Rnf165, Brd8, Plek, Eif2s2, Daam1, Dcun1d1, Zfp260, Zmym5, Hecw2, R3hdm2, Lin54, Gpr34

MSTRG.3640.2	miR-694, miR-466k, miR-5101, miR-669f-3p, miR-686, miR-466a-3p, miR-466d-3p, miR-466e-3p, miR-466n-3p, miR-335-3p, miR-466l-3p	Pex19, AW554918, Rnf24, P2ry13, Epha4, Arnt2, Lpl, Dzank1, Clcn3, Prkcd, Tmem175, Wscd1, Cdc42ep4, Clec7a, Mgll, Rin2, 5730455P16Rik, Tbcel, Rnf165, Brd8, Plek, Eif2s2, Daam1, Dcun1d1, Zfp260, Zmym5, Hecw2, R3hdm2, Lin54, Gpr34
MSTRG.3877.7	miR-694, miR-466k, miR-5101, miR-335-3p	Pex19, AW554918, Rnf24, P2ry13, Epha4, Arnt2, Lpl, Dzank1, Clcn3, Prkcd, Tmem175, Wscd1, Cdc42ep4, Clec7a, Mgll, Rin2, 5730455P16Rik, Tbcel, Rnf165, Brd8, Plek, Eif2s2, Daam1, Dcun1d1, Hecw2, Gpr34
ENSMUST00000182231	miR-329-3p	Dzank1, Cct7, Epha4, Prkcd, Clec7a
ENSMUST00000155277	miR-329-3p, miR-466a-3p, miR-466d-3p, miR-466e-3p, miR-466n-3p	Dzank1, Cct7, Epha4, Prkcd, Clec7a, Zmym5, Lin54, Hecw2
MSTRG.1243.32	miR-329-3p, miR-5101, miR-669f-3p, miR-466a-3p, miR-466d-3p, miR-466e-3p, miR-466n-3p, miR-335-3p	Dzank1, Cct7, Epha4, Prkcd, Clec7a, 5730455P16Rik, Tbcel, Rnf165, Brd8, Plek, Eif2s2, Daam1, Tmem175, Dcun1d1, Lpl, Rin2, Zfp260, Zmym5, Hecw2, Lin54, Gpr34
MSTRG.16327.2	miR-466k	Dzank1, Epha4, Clcn3, Prkcd, Tmem175, Wscd1, Cdc42ep4, Clec7a, Mgll, Rin2
ENSMUST00000126335	miR-5101	5730455P16Rik, Tbcel, Rnf165, Brd8, Plek, Eif2s2, Daam1, Tmem175, Dcun1d1, Lpl, Rin2
ENSMUST00000189564	miR-669f-3p, miR-335-3p	5730455P16Rik, Zfp260, Zmym5, Rnf165, Plek, Hecw2, Prkcd, Gpr34, Epha4, Tmem175
ENSMUST00000111019	miR-669f-3p, miR-686, miR-466a-3p, miR-466d-3p, miR-466e-3p, miR-335-3p	5730455P16Rik, Zfp260, Zmym5, Rnf165, Plek, Hecw2, Prkcd, Daam1, R3hdm2, Mgll, Lin54, Gpr34, Epha4, Tmem175
ENSMUST00000205779	miR-686	5730455P16Rik, Daam1, R3hdm2, Mgll

ENSMUST00000221971	miR-466a-3p, miR-466d-3p, miR-466e-3p	Zmym5, Lin54, Hecw2, Prkcd

Table S5. Correlated information of the downregulated lncRNA-associated ceRNA network in the cerebral cortex of 5×FAD mice.

Downregulated lncRNA	Upregulated miRNA	Downregulated mRNA
ENSMUST00000145741	miR-19b-3p, miR-743b-3p	Dpp6, Zcchc2, Hecw2, Npnt, Daam1, Tardbp, Hnrnpa3, Ttc14, Tmem175, Dcun1d1
MSTRG.18699.5	miR-19b-3p, miR-340-5p, miR-339-5p, miR-466i-5p	Dpp6, Zcchc2, Hecw2, Npnt, Daam1, Tardbp, R3hdm2, Brsk2, Slc4a10, Ank3, Dclk1, Slc1a2, Tmem175, Zfp68
MSTRG.8598.3	miR-19b-3p	Dpp6, Zcchc2, Hecw2, Npnt, Daam1
MSTRG.17500.1	miR-340-5p, miR-466i-5p	Tardbp, Npnt, R3hdm2, Brsk2, Slc4a10, Ank3, Slc1a2, Tmem175, Zfp68
MSTRG.7748.1	miR-340-5p, miR-743b-3p, miR-339-5p, miR-337-3p, miR-466i-5p	Tardbp, Npnt, R3hdm2, Brsk2, Slc4a10, Ank3, Hnrnpa3, Ttc14, Tmem175, Dcun1d1, Dclk1, Slc1a2, Zfp68
ENSMUST00000184170	miR-743b-3p, miR-214-3p	Tardbp, Hnrnpa3, Ttc14, Tmem175, Dcun1d1, Klc1
ENSMUST00000141357	miR-743b-3p, miR-339-5p, miR-337-3p	Tardbp, Hnrnpa3, Ttc14, Tmem175, Dcun1d1, Dclk1, Slc1a2
ENSMUST00000182451	miR-666-3p	Hnrnpa3, Akt2, Sulf2
ENSMUST00000161637	miR-666-3p, miR-337-3p	Hnrnpa3, Akt2, Sulf2, Slc1a2
ENSMUST00000129857	miR-666-3p, miR-337-3p	Hnrnpa3, Akt2, Sulf2, Slc1a2
ENSMUST00000199237	miR-666-3p	Hnrnpa3, Akt2, Sulf2
ENSMUST00000162401	miR-337-3p, miR-214-3p	Slc1a2, Klc1
ENSMUST00000141539	miR-214-3p	Klc1

Table S6. Information of the upregulated lncRNA-miRNA network in the hippocampus of 7-month-old 5×FAD mice.

Upregulated lncRNA	miRNA targets
ENSMUST00000180635	miR-3059-5p
ENSMUST00000180635	miR-1943-5p
ENSMUST00000180635	miR-7016-5p
ENSMUST00000180635	miR-3089-5p
ENSMUST00000180635	miR-330-5p
ENSMUST00000180635	miR-6915-5p
ENSMUST00000180635	miR-540-3p
ENSMUST00000180635	miR-1934-3p
ENSMUST00000180635	miR-505-5p
ENSMUST00000180635	miR-344g-5p
ENSMUST00000180635	miR-344d-1-5p
ENSMUST00000180635	miR-344d-3-5p
ENSMUST00000180635	miR-344-5p
ENSMUST00000180635	miR-3076-5p
ENSMUST00000180635	miR-7058-3p
ENSMUST00000180635	miR-669p-5p
ENSMUST00000180635	miR-31-5p
ENSMUST00000180635	miR-3072-5p
ENSMUST00000180635	miR-3068-5p
ENSMUST00000180635	miR-7055-5p
ENSMUST00000180635	miR-6979-3p
ENSMUST00000180635	miR-6918-5p
ENSMUST00000180635	miR-7687-5p
ENSMUST00000180635	miR-7231-5p
ENSMUST00000180635	miR-7029-5p
ENSMUST00000180635	miR-1968-3p
ENSMUST00000180635	miR-668-3p
ENSMUST00000180635	miR-6975-3p
ENSMUST00000180635	miR-698-5p
ENSMUST00000180635	miR-7036a-3p
ENSMUST00000180635	miR-3069-5p
ENSMUST00000180635	miR-335-3p
ENSMUST00000180635	miR-6936-5p
ENSMUST00000180635	miR-5128
ENSMUST00000180635	miR-1964-5p
ENSMUST00000180635	miR-6896-3p
ENSMUST00000180635	miR-664-5p
ENSMUST00000180635	miR-6987-5p
ENSMUST00000180635	miR-7115-5p
ENSMUST00000180635	miR-7093-5p
ENSMUST00000180635	miR-674-3p

ENSMUST00000180635	miR-3085-3p
ENSMUST00000180635	miR-193a-5p
ENSMUST00000180635	miR-3064-5p
ENSMUST00000197854	miR-1955-5p
ENSMUST00000197854	miR-760-3p
ENSMUST00000197854	miR-3084-3p
ENSMUST00000197854	miR-302b-3p
ENSMUST00000197854	miR-140-5p
ENSMUST00000197854	miR-330-5p
ENSMUST00000197854	miR-673-5p
ENSMUST00000197854	miR-7a-5p
ENSMUST00000197854	miR-7b-5p
ENSMUST00000197854	miR-674-3p
ENSMUST00000197854	miR-488-3p
ENSMUST00000197854	miR-181b-5p
ENSMUST00000197854	miR-3102-3p
ENSMUST00000197854	miR-485-5p
ENSMUST00000197854	miR-1943-5p
ENSMUST00000197854	miR-7016-5p
ENSMUST00000197854	miR-3109-5p
ENSMUST00000197854	miR-5617-3p
ENSMUST00000197854	miR-7214-5p
ENSMUST00000197854	miR-3154
ENSMUST00000197854	miR-5619-3p
ENSMUST00000197854	miR-7037-5p
ENSMUST00000197854	miR-137-3p
ENSMUST00000197854	miR-7015-3p
ENSMUST00000127786	miR-1198-5p
ENSMUST00000127786	miR-27b-3p
ENSMUST00000127786	miR-758-3p
ENSMUST00000127786	miR-6540-5p
ENSMUST00000127786	miR-23a-3p
ENSMUST00000127786	miR-23b-3p
ENSMUST00000127786	miR-145b
ENSMUST00000127786	miR-145a-5p
ENSMUST00000127786	miR-300-3p
ENSMUST00000127786	miR-505-5p
ENSMUST00000127786	miR-362-5p
ENSMUST00000127786	miR-302b-3p
ENSMUST00000127786	miR-221-3p
ENSMUST00000127786	miR-222-3p
ENSMUST00000127786	miR-26a-5p
ENSMUST00000127786	miR-140-5p
ENSMUST00000127786	miR-338-5p

ENSMUST00000127786	let-7i-5p
ENSMUST00000127786	let-7d-5p
ENSMUST00000127786	let-7a-5p
ENSMUST00000127786	let-7e-5p
ENSMUST00000127786	let-7f-5p
ENSMUST00000127786	miR-98-5p
ENSMUST00000127786	let-7g-5p
ENSMUST00000127786	miR-664-3p
ENSMUST00000127786	miR-384-3p
ENSMUST00000127786	miR-501-3p
ENSMUST00000127786	miR-3064-5p
ENSMUST00000127786	miR-3085-3p
ENSMUST00000127786	miR-664-5p
ENSMUST00000127786	miR-137-3p
ENSMUST00000127786	miR-3098-5p
ENSMUST00000127786	miR-1943-5p
ENSMUST00000127786	miR-7016-5p
ENSMUST00000127786	miR-344d-1-5p
ENSMUST00000127786	miR-344d-3-5p
ENSMUST00000127786	miR-344g-5p
ENSMUST00000127786	miR-450a-2-3p
ENSMUST00000127786	miR-471-5p
ENSMUST00000127786	miR-470-5p
ENSMUST00000127786	miR-3059-5p
ENSMUST00000127786	miR-342-3p
ENSMUST00000127786	miR-377-3p
ENSMUST00000127786	miR-425-5p
ENSMUST00000127786	miR-489-3p
ENSMUST00000127786	miR-493-5p
ENSMUST00000127786	miR-466b-3p
ENSMUST00000127786	miR-466c-3p
ENSMUST00000127786	miR-466p-3p
ENSMUST00000127786	miR-466m-3p
ENSMUST00000127786	miR-466o-3p
ENSMUST00000127786	miR-494-3p
ENSMUST00000127786	miR-539-5p
ENSMUST00000127786	miR-411-3p
ENSMUST00000127786	miR-379-3p
ENSMUST00000127786	miR-186-5p
ENSMUST00000127786	miR-344-5p
ENSMUST00000127786	miR-340-5p
ENSMUST00000127786	miR-9-3p
ENSMUST00000127786	miR-148a-3p
ENSMUST00000127786	miR-148b-3p

ENSMUST00000127786	miR-30a-5p
ENSMUST00000127786	miR-30e-5p
ENSMUST00000127786	miR-30d-5p
ENSMUST00000127786	miR-30c-5p
ENSMUST00000127786	miR-384-5p
ENSMUST00000127786	miR-185-5p
ENSMUST00000127786	miR-331-3p
ENSMUST00000127786	miR-146b-5p
ENSMUST00000127786	miR-31-5p
ENSMUST00000127786	miR-1971
ENSMUST00000127786	miR-1981-5p
ENSMUST00000127786	miR-1933-3p
ENSMUST00000127786	miR-29b-3p
ENSMUST00000127786	miR-29a-3p
ENSMUST00000127786	miR-466d-3p
ENSMUST00000127786	miR-467g
ENSMUST00000127786	miR-466a-3p
ENSMUST00000127786	miR-466e-3p
ENSMUST00000127786	miR-3102-3p
ENSMUST00000127786	miR-133a-3p
ENSMUST00000127786	miR-486a-5p
ENSMUST00000127786	miR-486b-5p
ENSMUST00000127786	miR-191-5p
ENSMUST00000127786	miR-106a-5p
ENSMUST00000127786	miR-20a-5p
ENSMUST00000127786	miR-7a-5p
ENSMUST00000127786	miR-7b-5p
ENSMUST00000127786	miR-125a-5p
ENSMUST00000127786	miR-125b-5p
ENSMUST00000127786	miR-351-5p
ENSMUST00000127786	miR-301a-3p
ENSMUST00000127786	miR-19b-3p
ENSMUST00000127786	miR-19a-3p
ENSMUST00000127786	miR-488-3p
ENSMUST00000127786	miR-743b-3p
ENSMUST00000127786	miR-679-3p
ENSMUST00000127786	miR-582-5p
ENSMUST00000127786	miR-350-5p
ENSMUST00000127786	miR-3093-3p
ENSMUST00000127786	miR-329-5p
ENSMUST00000127786	miR-3083-5p
ENSMUST00000127786	miR-346-3p
ENSMUST00000127786	miR-361-5p
ENSMUST00000127786	miR-127-3p

ENSMUST00000127786	miR-3087-3p
ENSMUST00000127786	miR-615-3p
ENSMUST00000127786	miR-129-2-3p
ENSMUST00000127786	miR-129-1-3p
ENSMUST00000127786	miR-329-3p
ENSMUST00000127786	miR-3078-5p
ENSMUST00000127786	miR-15b-5p
ENSMUST00000127786	miR-322-5p
ENSMUST00000127786	miR-1197-3p
ENSMUST00000127786	miR-6919-5p
ENSMUST00000127786	miR-152-5p
ENSMUST00000127786	miR-3473d
ENSMUST00000127786	miR-6984-5p
ENSMUST00000127786	miR-665-5p
ENSMUST00000127786	miR-7092-3p
ENSMUST00000127786	miR-330-3p
ENSMUST00000127786	miR-383-3p
ENSMUST00000127786	miR-1948-5p
ENSMUST00000127786	miR-30c-2-3p
ENSMUST00000127786	miR-760-5p
ENSMUST00000127786	miR-103-3p
ENSMUST00000127786	miR-880-3p
ENSMUST00000127786	miR-465a-5p
ENSMUST00000127786	miR-7116-5p
ENSMUST00000127786	miR-6371
ENSMUST00000127786	miR-543-3p
ENSMUST00000127786	miR-1191a
ENSMUST00000127786	miR-6936-5p
ENSMUST00000127786	miR-221-5p
ENSMUST00000127786	miR-673-5p
ENSMUST00000127786	miR-135a-5p
ENSMUST00000127786	miR-129b-3p
ENSMUST00000127786	miR-1191b-5p
ENSMUST00000127786	miR-543-5p
ENSMUST00000127786	miR-3069-5p
ENSMUST00000127786	miR-3066-5p
ENSMUST00000127786	miR-6933-3p
ENSMUST00000127786	miR-201-5p
ENSMUST00000127786	miR-551b-5p
ENSMUST00000127786	miR-217-5p
ENSMUST00000127786	miR-410-3p
ENSMUST00000127786	miR-6918-5p
ENSMUST00000127786	miR-148b-5p
ENSMUST00000127786	miR-423-5p

ENSMUST00000127786	miR-7080-3p
ENSMUST00000127786	miR-3057-5p
ENSMUST00000127786	miR-7655-3p
ENSMUST00000127786	miR-344b-3p
ENSMUST00000127786	miR-20a-3p
ENSMUST00000127786	miR-7665-5p
ENSMUST00000127786	miR-6896-5p
ENSMUST00000127786	miR-3089-5p
ENSMUST00000127786	miR-7675-3p
ENSMUST00000127786	miR-6911-5p
ENSMUST00000127786	miR-7674-3p
ENSMUST00000127786	miR-3967
ENSMUST00000127786	miR-1927
ENSMUST00000127786	miR-1946b
ENSMUST00000127786	miR-3086-3p
ENSMUST00000127786	miR-421-3p
ENSMUST00000127786	miR-153-3p
ENSMUST00000127786	miR-338-3p
ENSMUST00000127786	miR-128-3p
ENSMUST00000127786	miR-6982-5p
ENSMUST00000127786	miR-330-5p
ENSMUST00000127786	miR-381-3p
ENSMUST00000127786	miR-6899-3p
ENSMUST00000127786	miR-6976-5p
ENSMUST00000127786	miR-6896-3p
ENSMUST00000127786	miR-694
ENSMUST00000127786	miR-5617-5p
ENSMUST00000127786	miR-3069-3p
ENSMUST00000127786	miR-3094-3p
ENSMUST00000127786	miR-344e-3p
ENSMUST00000127786	miR-5709-5p
ENSMUST00000127786	miR-493-3p
ENSMUST00000127786	miR-7236-3p
ENSMUST00000127786	miR-1843a-5p
ENSMUST00000127786	miR-192-5p
ENSMUST00000127786	miR-1912-3p
ENSMUST00000127786	miR-210-5p
ENSMUST00000127786	miR-7054-5p
ENSMUST00000127786	miR-6537-3p
ENSMUST00000127786	miR-8113
ENSMUST00000127786	miR-505-3p
ENSMUST00000127786	miR-7019-5p
ENSMUST00000127786	miR-501-5p
ENSMUST00000127786	miR-6539

ENSMUST00000127786	miR-125b-2-3p
ENSMUST00000127786	let-7f-2-3p
ENSMUST00000127786	miR-669l-5p
ENSMUST00000127786	miR-335-3p
ENSMUST00000127786	miR-7013-3p
ENSMUST00000127786	miR-409-5p
ENSMUST00000127786	miR-503-3p
ENSMUST00000127786	miR-5621-3p
ENSMUST00000127786	miR-382-3p
ENSMUST00000127786	miR-6979-3p
ENSMUST00000127786	miR-6906-5p
ENSMUST00000127786	miR-3109-5p
ENSMUST00000127786	miR-3970
ENSMUST00000127786	miR-1955-3p
ENSMUST00000127786	miR-1931
ENSMUST00000127786	miR-345-5p
ENSMUST00000127786	miR-7056-3p
ENSMUST00000127786	miR-674-3p
ENSMUST00000127786	miR-544-3p
ENSMUST00000127786	miR-7117-5p
ENSMUST00000127786	miR-7226-3p
ENSMUST00000127786	miR-3074-1-3p
ENSMUST00000127786	miR-1969
ENSMUST00000127786	miR-299a-5p
ENSMUST00000127786	miR-8111
ENSMUST00000127786	miR-344d-2-5p
ENSMUST00000127786	miR-3095-3p
ENSMUST00000127786	miR-215-5p
ENSMUST00000127786	miR-3154
ENSMUST00000127786	miR-155-3p
ENSMUST00000127786	miR-3475-3p
ENSMUST00000127786	miR-7015-3p
ENSMUST00000127786	miR-3112-5p
ENSMUST00000127786	miR-7037-5p
ENSMUST00000127786	miR-669e-5p
ENSMUST00000127786	miR-511-5p
ENSMUST00000127786	miR-669a-5p
ENSMUST00000127786	miR-669p-5p
ENSMUST00000127786	miR-1298-3p
ENSMUST00000127786	miR-6937-3p
ENSMUST00000127786	miR-107-5p
ENSMUST00000127786	miR-6914-3p
ENSMUST00000127786	miR-7116-3p
ENSMUST00000127786	miR-7013-5p

ENSMUST00000127786	miR-8114
ENSMUST00000127786	miR-30f
ENSMUST00000127786	miR-187-5p
ENSMUST00000127786	miR-541-3p
ENSMUST00000127786	miR-3086-5p
ENSMUST00000127786	miR-344c-3p
ENSMUST00000127786	miR-874-3p
ENSMUST00000127786	miR-216b-5p
ENSMUST00000127786	miR-21a-3p
ENSMUST00000127786	miR-1983
ENSMUST00000127786	miR-6902-3p
ENSMUST00000127786	miR-411-5p
ENSMUST00000127786	miR-181b-5p
ENSMUST00000127786	miR-222-5p
ENSMUST00000127786	miR-6911-3p
ENSMUST00000127786	miR-412-3p
ENSMUST00000127786	miR-7682-3p
ENSMUST00000127786	miR-7077-5p
ENSMUST00000127786	miR-3062-5p
ENSMUST00000127786	miR-540-5p
ENSMUST00000127786	miR-328-3p
ENSMUST00000127786	miR-7038-3p
ENSMUST00000127786	miR-431-3p
ENSMUST00000127786	miR-6913-5p
ENSMUST00000127786	miR-7039-3p
ENSMUST00000127786	miR-186-3p
ENSMUST00000127786	miR-6991-5p
ENSMUST00000127786	miR-344-3p
ENSMUST00000127786	miR-344d-3p
ENSMUST00000127786	miR-203-5p
ENSMUST00000127786	miR-1b-5p
ENSMUST00000127786	miR-7115-3p
ENSMUST00000127786	miR-201-3p
ENSMUST00000127786	miR-3064-3p
ENSMUST00000127786	miR-3058-3p
ENSMUST00000127786	miR-7651-5p
ENSMUST00000127786	miR-693-3p
ENSMUST00000127786	miR-6975-3p
ENSMUST00000127786	miR-702-5p
ENSMUST00000127786	miR-345-3p
ENSMUST00000127786	miR-667-3p
ENSMUST00000127786	miR-7224-5p
ENSMUST00000127786	miR-5623-5p
ENSMUST00000144016	miR-6989-3p

ENSMUST00000141374	miR-125b-2-3p
ENSMUST00000141374	let-7a-2-3p
ENSMUST00000141374	miR-210-5p
ENSMUST00000141374	miR-5134-5p
ENSMUST00000028291	miR-3085-3p
ENSMUST00000028291	miR-3064-5p
ENSMUST00000028291	miR-344d-2-5p
ENSMUST00000028291	miR-1968-5p
ENSMUST00000028291	miR-3544-3p
ENSMUST00000028291	miR-3070-5p
ENSMUST00000028291	miR-346-3p
ENSMUST00000028291	miR-667-5p
ENSMUST00000028291	miR-6987-5p
ENSMUST00000028291	miR-7685-3p
ENSMUST00000028291	miR-466k
ENSMUST00000028291	miR-328-3p
ENSMUST00000028291	miR-1191a
ENSMUST00000154192	miR-7069-3p
ENSMUST00000154192	miR-1968-5p
ENSMUST00000154192	miR-330-5p
ENSMUST00000154192	miR-7115-5p
ENSMUST00000154192	miR-5123
ENSMUST00000154192	miR-5619-3p
ENSMUST00000153923	miR-20a-5p
ENSMUST00000153923	miR-106a-5p
ENSMUST00000153923	miR-667-5p
ENSMUST00000153923	miR-6896-5p
ENSMUST00000153923	miR-322-5p
ENSMUST00000153923	miR-15b-5p
ENSMUST00000153923	miR-21a-3p
ENSMUST00000153923	miR-6979-3p
ENSMUST00000153923	miR-23b-3p
ENSMUST00000153923	miR-23a-3p
ENSMUST00000153923	miR-3473d
ENSMUST00000153923	miR-694
ENSMUST00000153923	miR-3094-3p
ENSMUST00000153923	miR-7093-5p
ENSMUST00000218620	miR-7651-5p
ENSMUST00000218620	miR-29b-3p
ENSMUST00000218620	miR-29a-3p
ENSMUST00000218620	let-7j
ENSMUST00000218620	miR-485-5p
ENSMUST00000218620	miR-6958-3p
ENSMUST00000218620	miR-125b-2-3p

ENSMUST00000218620	miR-541-5p
ENSMUST00000218620	miR-7687-5p
ENSMUST00000218620	miR-193a-5p
ENSMUST00000218620	miR-29a-5p
ENSMUST00000211218	miR-7029-3p
ENSMUST00000150127	miR-504-3p
ENSMUST00000150127	miR-664-5p
ENSMUST00000150127	miR-7054-5p
ENSMUST00000150127	miR-484
ENSMUST00000150127	miR-330-5p
ENSMUST00000150127	miR-7116-3p
ENSMUST00000150127	miR-7052-3p
ENSMUST00000150127	miR-383-3p
ENSMUST00000150127	miR-185-5p
ENSMUST00000150127	miR-3087-5p
ENSMUST00000150127	miR-3090-3p
ENSMUST00000150127	miR-6984-5p
ENSMUST00000150127	miR-1249-5p
ENSMUST00000150127	miR-3085-3p
ENSMUST00000150127	miR-698-5p
ENSMUST00000150127	miR-329-5p
ENSMUST00000150127	miR-7671-5p
ENSMUST00000150127	miR-98-3p
ENSMUST00000150127	let-7f-1-3p
ENSMUST00000150127	let-7c-2-3p
ENSMUST00000150127	let-7a-1-3p
ENSMUST00000150127	miR-450a-2-3p
ENSMUST00000150127	miR-551b-5p
ENSMUST00000150127	miR-7093-3p
ENSMUST00000150127	miR-667-3p
ENSMUST00000150127	miR-5113
ENSMUST00000150127	miR-1968-3p
ENSMUST00000150127	miR-3064-5p
ENSMUST00000150127	miR-103-3p
ENSMUST00000152985	miR-5134-5p
ENSMUST00000152985	miR-6896-3p
ENSMUST00000152985	miR-344d-2-5p
ENSMUST00000152985	miR-30c-5p
ENSMUST00000152985	miR-325-3p
ENSMUST00000152985	miR-29b-1-5p
ENSMUST00000152985	miR-384-5p
ENSMUST00000152985	miR-30e-5p
ENSMUST00000152985	miR-30d-5p
ENSMUST00000152985	miR-30a-5p

ENSMUST00000152985	miR-423-3p
ENSMUST00000152985	miR-7226-3p
ENSMUST00000152985	miR-3069-5p
ENSMUST00000152985	miR-365-3p
ENSMUST00000152985	miR-7214-5p
ENSMUST00000152985	miR-5623-5p
ENSMUST00000152985	miR-7093-3p
ENSMUST00000152985	miR-6919-5p
ENSMUST00000145174	miR-7116-3p
ENSMUST00000145174	miR-185-5p
ENSMUST00000145174	miR-7055-5p
ENSMUST00000145174	miR-6900-5p
ENSMUST00000145174	miR-3083-5p
ENSMUST00000145174	miR-5617-3p
ENSMUST00000145174	miR-504-3p
ENSMUST00000132577	miR-1968-3p
ENSMUST00000132577	miR-1968-5p
ENSMUST00000132577	miR-1843b-5p
ENSMUST00000132577	miR-3084-3p
ENSMUST00000132577	miR-3093-3p
ENSMUST00000132577	miR-185-5p
ENSMUST00000132577	miR-666-5p
ENSMUST00000132577	miR-3097-3p
ENSMUST00000132577	miR-6912-5p
ENSMUST00000132577	miR-27b-3p
ENSMUST00000132577	miR-365-2-5p
ENSMUST00000132577	miR-365-1-5p
ENSMUST00000132577	miR-6946-3p
ENSMUST00000132577	miR-511-5p
ENSMUST00000132577	miR-667-5p
MSTRG.11359.1	miR-365-2-5p
MSTRG.11359.1	miR-365-1-5p
MSTRG.11359.1	miR-3069-3p
MSTRG.11359.1	miR-3093-3p
MSTRG.17500.2	miR-466k
MSTRG.17500.2	miR-7116-3p
MSTRG.17500.2	miR-466o-3p
MSTRG.17500.2	miR-466m-3p
MSTRG.17500.2	miR-7116-5p
MSTRG.17500.2	miR-6902-5p
MSTRG.17500.2	miR-7117-5p
MSTRG.17500.2	miR-6979-3p
MSTRG.17500.2	miR-6944-3p
MSTRG.17500.2	miR-3083-5p

MSTRG.17500.2	miR-3059-5p
MSTRG.17500.2	miR-3090-3p
MSTRG.17500.2	miR-6940-3p
MSTRG.17500.2	miR-185-5p
MSTRG.17500.2	miR-7675-3p
MSTRG.17500.2	miR-6896-3p
MSTRG.17500.2	miR-6948-5p
MSTRG.17500.2	miR-7013-5p
MSTRG.17500.2	miR-467g
MSTRG.17500.2	miR-466e-3p
MSTRG.17500.2	miR-466d-3p
MSTRG.17500.2	miR-466a-3p
MSTRG.17500.2	miR-210-5p
MSTRG.17500.2	miR-664-5p
MSTRG.17500.2	miR-1194
MSTRG.17500.2	miR-7214-5p
MSTRG.17500.2	miR-1968-5p
MSTRG.17500.2	miR-340-5p
MSTRG.17500.2	miR-6975-3p
MSTRG.17500.2	miR-6899-3p
MSTRG.17500.2	miR-5113
MSTRG.17500.2	miR-331-3p
MSTRG.17500.2	miR-1198-5p
MSTRG.17500.2	miR-3106-3p
MSTRG.17500.2	miR-365-2-5p
MSTRG.17500.2	miR-365-1-5p
MSTRG.17500.2	miR-7227-3p
MSTRG.17500.2	miR-6908-5p
MSTRG.17500.2	miR-539-5p
MSTRG.17500.2	miR-3057-5p
MSTRG.17500.2	miR-139-5p
MSTRG.17500.2	miR-450b-5p
MSTRG.17500.2	miR-6918-5p
MSTRG.17500.2	miR-342-3p
MSTRG.17500.2	miR-344g-5p
MSTRG.17500.2	miR-344d-3-5p
MSTRG.17500.2	miR-344d-1-5p
MSTRG.17500.2	miR-344-5p
MSTRG.17500.2	miR-3087-3p
MSTRG.17500.2	miR-344d-2-5p
MSTRG.17500.2	miR-6937-3p
MSTRG.17500.2	miR-7231-5p
MSTRG.17500.2	miR-377-3p
MSTRG.17500.2	miR-7118-5p

MSTRG.17500.2	miR-363-5p
MSTRG.17500.2	miR-3076-5p
MSTRG.17500.2	miR-1964-5p
MSTRG.17500.2	miR-5619-3p
MSTRG.17500.2	miR-6946-3p
MSTRG.17500.2	miR-7026-5p
MSTRG.17500.2	miR-6952-3p
MSTRG.17500.2	miR-1839-5p
MSTRG.17500.2	miR-7243-3p
MSTRG.17500.2	miR-433-5p
MSTRG.17500.2	miR-6900-3p
MSTRG.17500.2	miR-346-3p
MSTRG.17500.2	miR-7224-5p
MSTRG.17500.2	miR-694
MSTRG.17500.2	miR-338-5p
MSTRG.17500.2	miR-301b-5p
MSTRG.17500.2	miR-301a-5p
MSTRG.17500.2	miR-145b
MSTRG.17500.2	miR-145a-5p
MSTRG.17500.2	miR-133a-5p
MSTRG.17500.2	miR-450a-2-3p
MSTRG.17500.2	miR-1249-5p
MSTRG.17500.2	miR-7052-3p
MSTRG.17500.2	miR-6951-5p
MSTRG.17500.2	miR-466p-3p
MSTRG.17500.2	miR-466c-3p
MSTRG.17500.2	miR-466b-3p
MSTRG.17500.2	miR-7063-5p
MSTRG.17500.2	miR-329-3p
MSTRG.17500.2	miR-6958-3p
MSTRG.17500.2	miR-344i
MSTRG.17500.2	miR-1193-3p
MSTRG.17500.2	miR-7084-5p
MSTRG.17500.2	miR-7015-3p
MSTRG.17500.2	miR-493-5p
MSTRG.17500.2	miR-7093-3p
MSTRG.17500.2	miR-6994-5p
MSTRG.17500.2	miR-551b-5p
MSTRG.17500.2	miR-7077-3p
MSTRG.17500.2	miR-433-3p
MSTRG.17500.2	miR-1943-5p
MSTRG.17500.2	miR-3064-3p
MSTRG.17500.2	miR-330-5p
MSTRG.17500.2	miR-383-3p

MSTRG.17500.2	miR-7016-3p
MSTRG.17500.2	miR-7065-3p
MSTRG.17500.2	miR-706
MSTRG.17500.2	miR-222-5p
MSTRG.17500.2	miR-1948-5p
MSTRG.17500.2	miR-3085-3p
MSTRG.17500.2	miR-3064-5p
MSTRG.17500.2	miR-3112-3p
MSTRG.17500.2	miR-935
MSTRG.17500.2	miR-300-3p
MSTRG.17500.2	miR-152-5p
MSTRG.17500.2	miR-15b-5p
MSTRG.17500.2	miR-98-5p
MSTRG.17500.2	let-7i-5p
MSTRG.17500.2	let-7g-5p
MSTRG.17500.2	let-7f-5p
MSTRG.17500.2	let-7e-5p
MSTRG.17500.2	let-7a-5p
MSTRG.17500.2	miR-148b-3p
MSTRG.17500.2	miR-148a-3p
MSTRG.17500.2	miR-3094-3p
MSTRG.17500.2	miR-1941-3p
MSTRG.17500.2	miR-1983
MSTRG.17500.2	miR-6912-5p
MSTRG.17500.2	miR-488-5p
MSTRG.17500.2	miR-7658-3p
MSTRG.17500.2	miR-322-5p
MSTRG.17500.2	miR-412-3p
MSTRG.17500.2	miR-6953-3p
MSTRG.17500.2	miR-877-5p
MSTRG.17500.2	miR-3066-3p
MSTRG.17500.2	miR-8107
MSTRG.17500.2	miR-365-3p
MSTRG.17500.2	miR-7077-5p
MSTRG.17500.2	miR-7689-3p
MSTRG.17500.2	miR-3084-3p
MSTRG.17500.2	miR-698-5p
MSTRG.17500.2	miR-6948-3p
MSTRG.17500.2	miR-7016-5p
MSTRG.17500.2	miR-384-3p
MSTRG.17500.2	miR-377-5p
MSTRG.17500.2	let-7d-5p
MSTRG.17500.2	miR-7055-3p
MSTRG.17500.2	miR-370-5p

MSTRG.17500.2	miR-7656-5p
MSTRG.17500.2	let-7j
MSTRG.17500.2	miR-673-5p
MSTRG.17500.2	miR-873a-3p
MSTRG.17500.2	miR-346-5p
MSTRG.17500.2	miR-361-3p
MSTRG.17500.2	miR-186-5p
MSTRG.16327.2	miR-466k
MSTRG.16327.2	miR-28c
MSTRG.16327.2	miR-28b
MSTRG.16327.2	miR-23b-3p
MSTRG.16327.2	miR-23a-3p
MSTRG.16327.2	miR-3057-5p
MSTRG.16327.2	miR-7666-3p
MSTRG.16327.2	miR-6913-5p
MSTRG.16327.2	miR-30f
MSTRG.16327.2	miR-7651-5p
MSTRG.16327.2	miR-1964-5p
MSTRG.6530.1	miR-338-3p
MSTRG.6530.1	miR-98-3p
MSTRG.6530.1	let-7f-1-3p
MSTRG.6530.1	let-7c-2-3p
MSTRG.6530.1	let-7a-1-3p
MSTRG.6530.1	miR-5134-5p
MSTRG.6530.1	miR-6970-3p
MSTRG.6530.1	miR-1912-3p
MSTRG.6530.1	miR-186-5p
MSTRG.6775.12	miR-466o-3p
MSTRG.6775.12	miR-466m-3p
MSTRG.6775.12	miR-466p-3p
MSTRG.6775.12	miR-466c-3p
MSTRG.6775.12	miR-466b-3p
MSTRG.6775.12	miR-467g
MSTRG.6775.12	miR-7116-3p
MSTRG.6775.12	miR-466e-3p
MSTRG.6775.12	miR-466d-3p
MSTRG.6775.12	miR-466a-3p
MSTRG.6775.12	miR-1b-5p
MSTRG.6775.12	miR-466k
MSTRG.6775.12	miR-5619-5p
MSTRG.6775.12	miR-6951-5p
MSTRG.6775.12	miR-15b-3p
MSTRG.6775.12	miR-6944-3p
MSTRG.6775.12	miR-376b-5p

MSTRG.6775.12	miR-494-3p
MSTRG.6775.12	miR-7041-5p
MSTRG.6775.12	miR-383-5p
MSTRG.6775.12	miR-219c-3p
MSTRG.6775.12	miR-544-3p
MSTRG.6775.12	miR-6940-3p
MSTRG.6775.12	miR-361-5p
MSTRG.6775.12	miR-411-5p
MSTRG.6775.12	miR-137-3p
MSTRG.6775.12	miR-7092-3p
MSTRG.6775.12	miR-1198-5p
MSTRG.6775.12	miR-6946-3p
MSTRG.6775.12	miR-3094-3p
MSTRG.6775.12	miR-450b-5p
MSTRG.6775.12	miR-3084-3p
MSTRG.6775.12	miR-694
MSTRG.6775.12	miR-29a-5p
MSTRG.6775.12	miR-467f
MSTRG.6775.12	miR-7015-3p
MSTRG.6775.12	miR-338-3p
MSTRG.6775.12	miR-329-3p
MSTRG.6775.12	miR-6896-3p
MSTRG.6775.12	miR-3069-5p
MSTRG.6775.12	miR-664-3p
MSTRG.6775.12	miR-7a-1-3p
MSTRG.6775.12	miR-7018-3p
MSTRG.6775.12	miR-3066-3p
MSTRG.6775.12	miR-7116-5p
MSTRG.6775.12	miR-7669-5p
MSTRG.6775.12	miR-881-3p
MSTRG.6775.12	miR-7668-5p
MSTRG.6775.12	miR-7092-5p
MSTRG.6775.12	miR-186-5p
MSTRG.6775.12	miR-1231-3p
MSTRG.6775.12	miR-679-5p
MSTRG.6775.12	miR-410-3p
MSTRG.6775.12	miR-5123
MSTRG.6775.12	miR-3061-5p
MSTRG.6775.12	miR-344h-5p
MSTRG.6775.12	miR-344e-5p
MSTRG.6775.12	miR-668-3p
MSTRG.6775.12	miR-1927
MSTRG.6775.12	miR-7689-3p
MSTRG.6775.12	miR-7019-5p

MSTRG.6775.12	miR-6979-3p
MSTRG.6775.12	miR-543-3p
MSTRG.6775.12	miR-551b-5p
MSTRG.6775.12	miR-1929-5p
MSTRG.6775.12	miR-152-5p
MSTRG.6775.12	miR-880-3p
MSTRG.6775.12	miR-7013-5p
MSTRG.6775.12	miR-7118-3p
MSTRG.6775.12	miR-3058-3p
MSTRG.6775.12	miR-3535
MSTRG.6775.12	miR-7655-3p
MSTRG.6775.12	miR-365-3p
MSTRG.6775.12	miR-7063-5p
MSTRG.6775.12	miR-421-3p
MSTRG.6775.12	miR-7227-3p
MSTRG.6775.12	miR-6915-5p
MSTRG.6775.12	miR-540-3p
MSTRG.6775.12	miR-344g-5p
MSTRG.6775.12	miR-344d-3-5p
MSTRG.6775.12	miR-344d-1-5p
MSTRG.6775.12	miR-344-5p
MSTRG.10674.1	miR-466k
MSTRG.10674.1	miR-6901-5p
MSTRG.10674.1	miR-466p-3p
MSTRG.10674.1	miR-466c-3p
MSTRG.10674.1	miR-466b-3p
MSTRG.10674.1	miR-466m-3p
MSTRG.10674.1	miR-7054-5p
MSTRG.10674.1	miR-7116-3p
MSTRG.10674.1	miR-466o-3p
MSTRG.10674.1	miR-6540-5p
MSTRG.10674.1	miR-383-3p
MSTRG.10674.1	miR-6946-3p
MSTRG.10674.1	miR-30f
MSTRG.10674.1	miR-467f
MSTRG.10674.1	miR-7092-5p
MSTRG.10674.1	miR-7026-5p
MSTRG.10674.1	miR-582-3p
MSTRG.10674.1	miR-6912-5p
MSTRG.10674.1	miR-377-3p
MSTRG.10674.1	miR-6979-3p
MSTRG.10674.1	miR-3059-5p
MSTRG.10674.1	miR-8113
MSTRG.10674.1	miR-7012-5p

MSTRG.10674.1	miR-342-3p
MSTRG.10674.1	miR-6902-5p
MSTRG.10674.1	miR-329-3p
MSTRG.10674.1	miR-7b-5p
MSTRG.10674.1	miR-7a-5p
MSTRG.10674.1	miR-7227-3p
MSTRG.10674.1	miR-7116-5p
MSTRG.10674.1	miR-1198-5p
MSTRG.10674.1	miR-185-5p
MSTRG.10674.1	miR-6900-5p
MSTRG.10674.1	miR-6906-5p
MSTRG.10674.1	miR-201-5p
MSTRG.10674.1	miR-706
MSTRG.17055.1	miR-495-3p
MSTRG.17055.1	miR-3112-5p
MSTRG.17055.1	miR-466o-3p
MSTRG.17055.1	miR-466m-3p
MSTRG.17055.1	miR-706
MSTRG.17055.1	miR-351-5p
MSTRG.17055.1	miR-125b-5p
MSTRG.17055.1	miR-125a-5p
MSTRG.17055.1	miR-374c-3p
MSTRG.17055.1	miR-3070-3p
MSTRG.17055.1	miR-381-3p
MSTRG.17055.1	miR-7026-5p
MSTRG.17055.1	miR-488-5p
MSTRG.17055.1	miR-338-3p
MSTRG.17055.1	miR-346-3p
MSTRG.17055.1	miR-7116-3p
MSTRG.17055.1	miR-466p-3p
MSTRG.17055.1	miR-466c-3p
MSTRG.17055.1	miR-466b-3p
MSTRG.17055.1	miR-760-5p
MSTRG.17055.1	miR-673-5p
MSTRG.17055.1	miR-383-3p
MSTRG.17055.1	miR-5621-3p
MSTRG.14838.1	miR-3084-3p
MSTRG.14838.1	miR-7214-5p
MSTRG.14838.1	miR-377-5p
MSTRG.14838.1	miR-7671-5p
MSTRG.14838.1	miR-346-3p
MSTRG.1243.32	miR-7226-3p
MSTRG.1243.32	miR-467g
MSTRG.1243.32	miR-466o-3p

MSTRG.1243.32	miR-466m-3p
MSTRG.1243.32	miR-706
MSTRG.1243.32	miR-1b-5p
MSTRG.1243.32	miR-466e-3p
MSTRG.1243.32	miR-466d-3p
MSTRG.1243.32	miR-466a-3p
MSTRG.1243.32	miR-7092-3p
MSTRG.1243.32	miR-3967
MSTRG.1243.32	miR-222-3p
MSTRG.1243.32	miR-221-3p
MSTRG.1243.32	miR-466p-3p
MSTRG.1243.32	miR-466c-3p
MSTRG.1243.32	miR-466b-3p
MSTRG.1243.32	miR-7054-5p
MSTRG.1243.32	miR-484
MSTRG.1243.32	miR-181b-5p
MSTRG.1243.32	miR-6913-5p
MSTRG.1243.32	miR-7052-3p
MSTRG.1243.32	miR-7027-5p
MSTRG.1243.32	miR-329-3p
MSTRG.1243.32	miR-383-3p
MSTRG.1243.32	miR-6946-3p
MSTRG.1243.32	miR-504-3p
MSTRG.1243.32	miR-6958-3p
MSTRG.1243.32	miR-30c-2-3p
MSTRG.1243.32	miR-7116-3p
MSTRG.1243.32	miR-7b-5p
MSTRG.1243.32	miR-7a-5p
MSTRG.1243.32	miR-187-5p
MSTRG.1243.32	miR-1194
MSTRG.1243.32	miR-7669-5p
MSTRG.1243.32	miR-7015-5p
MSTRG.1243.32	miR-7115-3p
MSTRG.1243.32	miR-702-5p
MSTRG.1243.32	miR-335-3p
MSTRG.1243.32	miR-6902-3p
MSTRG.1243.32	miR-760-5p
MSTRG.1243.32	miR-501-5p
MSTRG.1243.32	miR-128-3p
MSTRG.1243.32	miR-6539
MSTRG.1243.32	miR-664-3p
MSTRG.1243.32	miR-743b-3p
MSTRG.1243.32	miR-488-3p
MSTRG.1243.32	miR-6983-5p

MSTRG.1243.32	miR-551b-5p
MSTRG.1243.32	miR-3068-5p
MSTRG.1243.32	miR-6911-5p
MSTRG.1243.32	miR-7055-5p
MSTRG.1243.32	miR-874-3p
MSTRG.1243.32	miR-8113
MSTRG.1243.32	miR-7012-5p
MSTRG.1243.32	miR-412-3p
MSTRG.1243.32	miR-148b-3p
MSTRG.1243.32	miR-148a-3p
MSTRG.1243.32	miR-7668-5p
MSTRG.1243.32	miR-330-5p
MSTRG.1243.32	miR-338-3p
MSTRG.1243.32	miR-1983
MSTRG.1243.32	miR-7081-3p
MSTRG.1243.32	miR-219a-1-3p
MSTRG.1243.32	miR-6901-5p
MSTRG.1243.32	miR-222-5p
MSTRG.1243.32	miR-365-3p
MSTRG.1243.32	miR-7043-3p
MSTRG.1243.32	miR-1981-3p
MSTRG.1243.32	miR-7018-3p
MSTRG.1243.32	miR-3090-3p
MSTRG.1243.32	miR-539-5p
MSTRG.1243.32	miR-129b-3p
MSTRG.1243.32	miR-495-5p
MSTRG.1243.32	miR-3082-3p
MSTRG.1243.32	miR-186-5p
MSTRG.1243.32	miR-1291
MSTRG.1243.32	let-7f-2-3p
MSTRG.1243.32	miR-7227-3p
MSTRG.1243.32	miR-679-5p
MSTRG.1243.32	miR-6991-5p
MSTRG.1243.32	miR-1668
MSTRG.1243.32	miR-673-5p
MSTRG.1243.32	miR-6918-5p
MSTRG.1243.32	miR-345-3p
MSTRG.1243.32	miR-139-5p
MSTRG.1243.32	miR-7651-5p
MSTRG.1243.32	miR-330-3p
MSTRG.1243.32	miR-298-3p
MSTRG.1243.32	miR-7665-5p

Table S7. Information of the downregulated lncRNA-miRNA network in the hippocampus of 7-month-old 5×FAD mice.

Downregulated lncRNA	miRNA targets
ENSMUST00000184170	miR-743a-3p
ENSMUST00000184170	miR-669b-5p
ENSMUST00000184170	miR-214-3p
ENSMUST00000184170	miR-3079-3p
ENSMUST00000184170	miR-24-3p
ENSMUST00000184170	miR-3060-3p
ENSMUST00000184170	miR-195a-5p
ENSMUST00000184170	miR-6419
ENSMUST00000184170	miR-15a-5p
ENSMUST00000184170	miR-16-5p
ENSMUST00000184170	miR-497a-5p
ENSMUST00000184170	miR-195b
ENSMUST00000184170	miR-3065-5p
ENSMUST00000184170	miR-193a-3p
ENSMUST00000184170	miR-193b-3p
ENSMUST00000184170	miR-465a-3p
ENSMUST00000184170	miR-465b-3p
ENSMUST00000184170	miR-465c-3p
ENSMUST00000184170	miR-18a-5p
ENSMUST00000184170	miR-7010-5p
ENSMUST00000184170	miR-7075-5p
ENSMUST00000184170	miR-8120
ENSMUST00000184170	miR-127-5p
ENSMUST00000184170	miR-7211-3p
ENSMUST00000184170	miR-151-5p
ENSMUST00000197200	miR-7008-3p
ENSMUST00000197200	miR-5101
ENSMUST00000197200	miR-6715-5p
ENSMUST00000197200	miR-320-3p
ENSMUST00000197200	miR-3093-5p
ENSMUST00000197200	miR-3470b
ENSMUST00000197200	miR-6384
ENSMUST00000197200	miR-6986-5p
ENSMUST00000197200	miR-7000-3p
ENSMUST00000197200	miR-5616-5p
ENSMUST00000197200	miR-7674-5p
ENSMUST00000197200	miR-669b-5p
ENSMUST00000197200	miR-7010-5p
ENSMUST00000197200	miR-6922-3p
ENSMUST00000197200	miR-6971-3p
ENSMUST00000197200	miR-6964-3p

ENSMUST00000197200	miR-6999-3p
ENSMUST00000197200	miR-143-3p
ENSMUST00000197200	miR-6928-3p
ENSMUST00000197200	miR-124-3p
ENSMUST00000197200	miR-224-5p
ENSMUST00000197200	miR-3572-3p
ENSMUST00000197200	miR-1231-5p
ENSMUST00000197200	miR-150-5p
ENSMUST00000197200	miR-146b-3p
ENSMUST00000197200	miR-7237-3p
ENSMUST00000197200	miR-6920-5p
ENSMUST00000197200	miR-5107-3p
ENSMUST00000197200	miR-5106
ENSMUST00000197200	miR-7056-5p
ENSMUST00000197200	miR-7061-5p
ENSMUST00000197200	miR-6996-5p
ENSMUST00000197200	miR-6769b-5p
ENSMUST00000197200	miR-7024-3p
ENSMUST00000197200	miR-3113-5p
ENSMUST00000197200	miR-5710
ENSMUST00000197200	miR-3079-5p
ENSMUST00000197200	miR-7649-3p
ENSMUST00000197200	miR-672-3p
ENSMUST00000197200	miR-3084-5p
ENSMUST00000197200	miR-3473f
ENSMUST00000197200	miR-7042-5p
ENSMUST00000197200	miR-1946a
ENSMUST00000197200	miR-7022-5p
ENSMUST00000197200	miR-8103
ENSMUST00000197200	miR-7087-5p
ENSMUST00000197200	miR-6998-5p
ENSMUST00000197200	miR-6947-5p
ENSMUST00000197200	miR-7066-3p
ENSMUST00000197200	miR-6516-3p
ENSMUST00000197200	miR-362-3p
ENSMUST00000197200	miR-6932-3p
ENSMUST00000197200	miR-6941-3p
ENSMUST00000197200	miR-6937-5p
ENSMUST00000197200	miR-3474
ENSMUST00000197200	miR-1934-5p
ENSMUST00000197200	miR-191-3p
ENSMUST00000197200	miR-5621-5p
ENSMUST00000197200	miR-7664-3p
ENSMUST00000197200	miR-1941-5p

ENSMUST00000197200	miR-6898-3p
ENSMUST00000197200	miR-7031-3p
ENSMUST00000197200	miR-25-3p
ENSMUST00000197200	miR-3100-5p
ENSMUST00000197200	miR-6985-5p
ENSMUST00000197200	miR-300-5p
ENSMUST00000197200	miR-103-1-5p
ENSMUST00000197200	miR-103-2-5p
ENSMUST00000197200	miR-136-5p
ENSMUST00000197200	miR-483-3p
ENSMUST00000197200	miR-7051-5p
ENSMUST00000197200	miR-205-5p
ENSMUST00000125930	miR-26a-2-3p
ENSMUST00000125930	miR-26b-3p
ENSMUST00000125930	miR-6964-3p
ENSMUST00000125930	miR-6935-3p
ENSMUST00000182575	miR-669c-3p
ENSMUST00000182575	miR-7649-3p
ENSMUST00000182575	miR-7073-5p
ENSMUST00000182575	miR-6925-5p
ENSMUST00000182575	miR-3473g
ENSMUST00000182575	miR-499-5p
ENSMUST00000182575	miR-323-5p
ENSMUST00000182575	miR-877-3p
ENSMUST00000182575	miR-448-3p
ENSMUST00000182575	miR-671-3p
ENSMUST00000182575	miR-1188-5p
ENSMUST00000182575	miR-3113-5p
ENSMUST00000182575	miR-6540-3p
ENSMUST00000182575	miR-6901-3p
ENSMUST00000182575	miR-7048-5p
ENSMUST00000182575	miR-185-3p
ENSMUST00000182575	let-7g-3p
ENSMUST00000182575	miR-3065-5p
ENSMUST00000182575	miR-181b-2-3p
ENSMUST00000182575	miR-181b-1-3p
ENSMUST00000182575	miR-7008-5p
ENSMUST00000182575	miR-3473e
ENSMUST00000182575	miR-3473b
ENSMUST00000182575	miR-6998-3p
ENSMUST00000182575	miR-25-5p
ENSMUST00000182575	miR-7017-5p
ENSMUST00000119305	miR-7054-3p
ENSMUST00000119305	miR-107-3p

ENSMUST00000119305	miR-30b-5p
ENSMUST00000119305	miR-3113-5p
ENSMUST00000119305	miR-7047-3p
ENSMUST00000119305	miR-30c-1-3p
ENSMUST00000119305	miR-547-5p
ENSMUST00000218432	miR-486b-3p
ENSMUST00000218432	miR-486a-3p
ENSMUST00000218432	miR-6996-5p
ENSMUST00000218432	miR-1930-3p
ENSMUST00000218432	miR-143-5p
ENSMUST00000218432	miR-7688-5p
ENSMUST00000138202	miR-191-3p
ENSMUST00000138202	miR-466l-3p
ENSMUST00000138202	miR-34a-3p
ENSMUST00000138202	miR-7075-5p
ENSMUST00000138202	miR-216b-3p
ENSMUST00000138202	miR-24-3p
ENSMUST00000138202	miR-7032-3p
ENSMUST00000138202	miR-466o-5p
ENSMUST00000138202	miR-466b-5p
ENSMUST00000138202	miR-670-5p
ENSMUST00000138202	miR-31-3p
ENSMUST00000138202	miR-7019-3p
ENSMUST00000138202	miR-378a-5p
ENSMUST00000138202	miR-3074-5p
ENSMUST00000138202	miR-7021-3p
ENSMUST00000138202	miR-6481
ENSMUST00000138202	miR-8103
ENSMUST00000138202	miR-18a-3p
ENSMUST00000138202	miR-7047-5p
ENSMUST00000138202	miR-7679-5p
ENSMUST00000138202	miR-3058-5p
ENSMUST00000138202	miR-6950-3p
ENSMUST00000182642	miR-20b-3p
ENSMUST00000182642	miR-17-3p
ENSMUST00000182642	miR-296-5p
ENSMUST00000182642	miR-5621-5p
ENSMUST00000182642	miR-103-2-5p
ENSMUST00000182642	miR-103-1-5p
ENSMUST00000182642	miR-351-3p
ENSMUST00000182642	miR-7674-5p
ENSMUST00000182642	miR-324-3p
ENSMUST00000182642	miR-378d
ENSMUST00000182642	miR-7021-3p

ENSMUST00000182642	miR-7659-5p
ENSMUST00000182642	miR-666-3p
ENSMUST00000182642	miR-6540-3p
ENSMUST00000182642	miR-466f-3p
ENSMUST00000182642	miR-7004-5p
ENSMUST00000182642	miR-669c-3p
ENSMUST00000182642	miR-6909-5p
ENSMUST00000162088	miR-669c-3p
ENSMUST00000162088	miR-7021-3p
ENSMUST00000162088	miR-744-3p
ENSMUST00000162088	miR-7679-5p
ENSMUST00000162088	miR-216a-5p
ENSMUST00000162088	miR-6419
ENSMUST00000162088	miR-195b
ENSMUST00000162088	miR-195a-5p
ENSMUST00000162088	miR-16-5p
ENSMUST00000162088	miR-15a-5p
ENSMUST00000162088	miR-324-5p
ENSMUST00000162088	miR-497a-5p
ENSMUST00000162088	miR-6984-3p
ENSMUST00000162088	miR-1961
ENSMUST00000162088	miR-127-5p
ENSMUST00000162088	miR-3964
ENSMUST00000203561	miR-503-5p
ENSMUST00000203561	miR-6921-3p
ENSMUST00000203561	miR-148a-5p
ENSMUST00000203561	miR-21b
ENSMUST00000203561	miR-26b-5p
ENSMUST00000203561	miR-669c-3p
ENSMUST00000203561	miR-126a-5p
ENSMUST00000203561	miR-8118
ENSMUST00000203561	miR-672-3p
ENSMUST00000203561	miR-7010-5p
ENSMUST00000203561	miR-7048-3p
ENSMUST00000203561	miR-3471
ENSMUST00000203561	miR-1954
ENSMUST00000203561	miR-5616-5p
ENSMUST00000203561	miR-298-5p
ENSMUST00000203561	miR-6997-5p
ENSMUST00000203561	miR-15a-5p
ENSMUST00000126582	miR-8103
ENSMUST00000126344	miR-7017-5p
ENSMUST00000126344	miR-7032-3p
ENSMUST00000126344	miR-7008-3p

ENSMUST00000126344	miR-743a-3p
ENSMUST00000126344	miR-5616-5p
ENSMUST00000126344	miR-146a-5p
ENSMUST00000126344	miR-5107-3p
ENSMUST00000126344	miR-3110-5p
ENSMUST00000126344	miR-6925-5p
ENSMUST00000126344	miR-34a-3p
ENSMUST00000168634	miR-188-3p
ENSMUST00000168634	miR-7022-5p
ENSMUST00000168634	miR-3474
ENSMUST00000168634	miR-7008-3p
ENSMUST00000151020	miR-302c-3p
ENSMUST00000151020	miR-204-3p
ENSMUST00000151020	miR-5101
ENSMUST00000151020	miR-149-5p
ENSMUST00000151020	miR-1264-5p
ENSMUST00000151020	miR-296-5p
ENSMUST00000151020	miR-466l-3p
ENSMUST00000151020	miR-326-3p
ENSMUST00000151020	miR-490-5p
ENSMUST00000151020	miR-199b-5p
ENSMUST00000151020	miR-199a-5p
ENSMUST00000151020	miR-3106-5p
ENSMUST00000151020	miR-27a-3p
ENSMUST00000151020	miR-7056-5p
ENSMUST00000151020	miR-669e-3p
ENSMUST00000151020	miR-339-5p
ENSMUST00000151020	miR-497a-3p
ENSMUST00000151020	miR-375-3p
ENSMUST00000151020	miR-7022-5p
ENSMUST00000151020	miR-6967-5p
ENSMUST00000151020	miR-7037-3p
ENSMUST00000151020	miR-7649-3p
ENSMUST00000151020	miR-130a-5p
ENSMUST00000151020	miR-6392-5p
ENSMUST00000151020	miR-7025-3p
ENSMUST00000134954	miR-6985-5p
ENSMUST00000134954	miR-5624-3p
ENSMUST00000134954	miR-669c-3p
ENSMUST00000134954	miR-669e-3p
ENSMUST00000134954	miR-214-3p
ENSMUST00000218286	miR-195b
ENSMUST00000218286	miR-195a-5p
ENSMUST00000218286	miR-16-5p

ENSMUST00000218286	miR-15a-5p
ENSMUST00000218286	miR-3474
ENSMUST00000218286	miR-6419
ENSMUST00000218286	miR-497a-5p
ENSMUST00000218286	miR-24-3p
ENSMUST00000218286	miR-700-5p
ENSMUST00000218286	miR-107-3p
ENSMUST00000218286	miR-6910-3p
ENSMUST00000218286	miR-490-5p
ENSMUST00000185523	miR-1954
ENSMUST00000185523	miR-3101-3p
ENSMUST00000185523	miR-3966
ENSMUST00000185523	miR-3095-5p
ENSMUST00000131037	miR-3075-5p
ENSMUST00000131037	miR-3552
ENSMUST00000131037	miR-449a-5p
ENSMUST00000131037	miR-34a-5p
ENSMUST00000131037	miR-7656-3p
ENSMUST00000131037	miR-449c-5p
ENSMUST00000131037	miR-449b
ENSMUST00000131037	miR-34c-5p
ENSMUST00000131037	miR-34b-5p
ENSMUST00000131037	miR-7213-5p
ENSMUST00000131037	miR-7679-5p
ENSMUST00000131037	miR-149-5p
ENSMUST00000131037	miR-3473e
ENSMUST00000131037	miR-3473b
ENSMUST00000131037	miR-652-3p
ENSMUST00000131037	miR-666-3p
ENSMUST00000173081	miR-1231-5p
ENSMUST00000173081	miR-6903-3p
ENSMUST00000173081	miR-205-5p
ENSMUST00000173081	miR-125a-3p
ENSMUST00000173081	miR-743a-3p
ENSMUST00000173081	miR-6982-3p
ENSMUST00000173081	miR-6950-3p
ENSMUST00000173081	miR-3113-5p
ENSMUST00000173081	miR-500-5p
ENSMUST00000173081	miR-6935-3p
ENSMUST00000173081	miR-466i-5p
ENSMUST00000173081	miR-466d-5p
ENSMUST00000173081	miR-20b-3p
ENSMUST00000173081	miR-17-3p
ENSMUST00000173081	miR-5621-5p

ENSMUST00000173081	miR-6984-3p
ENSMUST00000199803	miR-8118
ENSMUST00000199803	miR-6925-5p
ENSMUST00000199803	miR-448-3p
ENSMUST00000199803	miR-33-5p
ENSMUST00000199803	let-7b-3p
ENSMUST00000199803	miR-6985-3p
ENSMUST00000199803	miR-7034-3p
ENSMUST00000199803	miR-6944-5p
ENSMUST00000199803	miR-211-5p
ENSMUST00000199803	miR-204-5p
ENSMUST00000199803	miR-152-3p
ENSMUST00000199803	miR-1960
ENSMUST00000199803	miR-466i-3p
ENSMUST00000199803	miR-466l-3p
ENSMUST00000199803	miR-188-5p
ENSMUST00000199803	miR-669f-3p
ENSMUST00000199803	miR-669b-3p
ENSMUST00000199803	miR-467a-3p
ENSMUST00000199803	miR-6910-3p
ENSMUST00000199803	miR-322-3p
ENSMUST00000199803	miR-7051-5p
ENSMUST00000199803	miR-669c-3p
ENSMUST00000199803	miR-8097
ENSMUST00000199803	miR-487b-3p
ENSMUST00000220428	miR-3966
ENSMUST00000220428	miR-6922-3p
MSTRG.2483.2	miR-7037-3p
MSTRG.2483.2	miR-7213-5p
MSTRG.2483.2	miR-486b-3p
MSTRG.2483.2	miR-486a-3p
MSTRG.2483.2	miR-326-3p
MSTRG.2483.2	miR-22-3p
MSTRG.2483.2	miR-7674-5p
MSTRG.2483.2	miR-3110-5p
MSTRG.2483.2	miR-7021-3p
MSTRG.2483.2	miR-149-5p
MSTRG.2483.2	miR-7008-5p
MSTRG.2483.2	miR-24-3p
MSTRG.2483.2	miR-7062-5p
MSTRG.2483.2	miR-135a-1-3p
MSTRG.2483.2	miR-7009-3p
MSTRG.2483.2	miR-324-5p
MSTRG.2483.2	miR-18a-5p

MSTRG.2483.2	miR-143-3p
MSTRG.2483.2	miR-669c-3p
MSTRG.2483.2	miR-1949
MSTRG.2483.2	miR-764-3p
MSTRG.2483.2	miR-7075-5p
MSTRG.2483.2	miR-344c-5p
MSTRG.2483.2	miR-211-5p
MSTRG.2483.2	miR-204-5p
MSTRG.2483.2	miR-3102-3p.2-3p
MSTRG.2483.2	miR-6973b-3p
MSTRG.2483.2	miR-138-2-3p
MSTRG.2483.2	miR-1258-3p
MSTRG.2483.2	miR-6985-3p
MSTRG.2483.2	miR-878-5p
MSTRG.2483.2	miR-7649-3p
MSTRG.2483.2	miR-3470a
MSTRG.2483.2	miR-214-3p
MSTRG.2483.2	miR-483-3p
MSTRG.2483.2	miR-6715-5p
MSTRG.2483.2	miR-3095-5p
MSTRG.2483.2	miR-6997-5p
MSTRG.2483.2	miR-8097
MSTRG.2483.2	miR-7024-3p
MSTRG.2483.2	miR-3470b
MSTRG.2483.2	miR-665-3p
MSTRG.2483.2	miR-452-3p
MSTRG.2483.2	miR-27a-3p
MSTRG.2483.2	miR-3093-5p
MSTRG.2483.2	miR-136-5p
MSTRG.2483.2	miR-878-3p
MSTRG.2483.2	miR-670-3p
MSTRG.2483.2	miR-5125
MSTRG.2483.2	miR-378a-5p
MSTRG.2483.2	miR-6943-5p
MSTRG.2483.2	miR-6925-5p
MSTRG.2483.2	miR-1224-3p
MSTRG.2483.2	miR-3078-3p
MSTRG.2483.2	miR-20b-3p
MSTRG.2483.2	miR-17-3p
MSTRG.2483.2	miR-193b-5p
MSTRG.16159.9	miR-7019-3p
MSTRG.16159.9	miR-3552
MSTRG.16159.9	miR-7236-5p
MSTRG.16159.9	miR-6998-3p

MSTRG.17585.1	miR-466i-5p
MSTRG.17585.1	miR-466d-5p
MSTRG.17585.1	miR-126a-5p
MSTRG.17585.1	miR-200b-5p
MSTRG.17585.1	miR-6903-3p
MSTRG.17585.1	miR-200a-5p
MSTRG.17585.1	miR-181d-5p
MSTRG.17585.1	miR-181c-5p
MSTRG.17585.1	miR-181a-5p
MSTRG.17585.1	miR-6928-5p
MSTRG.17585.1	miR-203-3p
MSTRG.17585.1	miR-200c-5p
MSTRG.17585.1	miR-743a-3p
MSTRG.17585.1	miR-19b-1-5p
MSTRG.17585.1	miR-7087-5p
MSTRG.17585.1	miR-466h-5p
MSTRG.17585.1	miR-6967-5p
MSTRG.17585.1	miR-3082-5p
MSTRG.17585.1	miR-7656-3p
MSTRG.17585.1	miR-7057-3p
MSTRG.17585.1	miR-709
MSTRG.17585.1	miR-3108-5p
MSTRG.17585.1	miR-669m-5p
MSTRG.17585.1	miR-466m-5p
MSTRG.17585.1	miR-466j
MSTRG.17585.1	miR-7073-5p
MSTRG.17585.1	miR-421-5p
MSTRG.17585.1	miR-466n-5p
MSTRG.17585.1	miR-5101
MSTRG.17585.1	miR-146b-3p
MSTRG.17585.1	miR-465c-5p
MSTRG.17585.1	miR-465b-5p
MSTRG.17585.1	miR-155-5p
MSTRG.17585.1	miR-299b-3p
MSTRG.17585.1	miR-299a-3p
MSTRG.17585.1	miR-3472
MSTRG.17585.1	miR-6959-5p
MSTRG.17585.1	miR-466q
MSTRG.17585.1	miR-7024-5p
MSTRG.17585.1	miR-466p-5p
MSTRG.17585.1	miR-466a-5p
MSTRG.17585.1	miR-6926-5p
MSTRG.17585.1	miR-466e-5p
MSTRG.17585.1	miR-3474

MSTRG.17585.1	miR-3079-3p
MSTRG.17585.1	miR-194-2-3p
MSTRG.17585.1	miR-3100-5p
MSTRG.17585.1	miR-7008-3p
MSTRG.17585.1	miR-7009-3p
MSTRG.17585.1	miR-3065-5p
MSTRG.17585.1	miR-669c-3p
MSTRG.17585.1	miR-5624-3p
MSTRG.17585.1	miR-6516-3p
MSTRG.17585.1	miR-376c-3p
MSTRG.17585.1	miR-6941-5p
MSTRG.17585.1	miR-1187
MSTRG.17585.1	miR-3099-5p
MSTRG.17585.1	miR-669e-3p
MSTRG.17585.1	miR-542-3p
MSTRG.17585.1	miR-3552
MSTRG.17585.1	miR-669b-5p
MSTRG.17585.1	miR-3964
MSTRG.17585.1	miR-127-5p
MSTRG.17585.1	miR-136-5p
MSTRG.17585.1	miR-7025-3p
MSTRG.9309.1	miR-7236-5p
MSTRG.9309.1	miR-5101
MSTRG.9309.1	miR-669c-3p
MSTRG.9309.1	miR-467e-5p
MSTRG.9309.1	miR-669b-5p
MSTRG.9309.1	miR-669f-5p
MSTRG.9309.1	miR-7042-5p
MSTRG.9309.1	miR-709
MSTRG.9309.1	miR-1231-5p
MSTRG.9309.1	miR-3569-3p
MSTRG.9309.1	miR-669m-3p
MSTRG.9309.1	miR-669l-3p
MSTRG.9309.1	miR-467e-3p
MSTRG.9309.1	miR-467d-3p
MSTRG.9309.1	miR-467c-3p
MSTRG.9309.1	miR-467b-3p
MSTRG.9309.1	miR-6925-5p
MSTRG.9309.1	miR-376c-3p
MSTRG.9309.1	miR-7021-3p
MSTRG.9309.1	miR-216c-5p
MSTRG.9309.1	miR-3108-5p
MSTRG.9309.1	miR-350-3p
MSTRG.9309.1	miR-218-2-3p

MSTRG.9309.1	miR-7010-5p
MSTRG.9309.1	miR-6540-3p
MSTRG.9309.1	miR-6967-5p
MSTRG.9309.1	miR-669f-3p
MSTRG.9309.1	miR-669b-3p
MSTRG.9309.1	miR-467a-3p
MSTRG.9309.1	miR-135b-5p
MSTRG.9309.1	miR-6922-3p
MSTRG.9309.1	miR-22-5p
MSTRG.9309.1	miR-101b-3p
MSTRG.9309.1	miR-6937-5p
MSTRG.9309.1	miR-181d-5p
MSTRG.9309.1	miR-181c-5p
MSTRG.9309.1	miR-181a-5p
MSTRG.9309.1	miR-466f-3p
MSTRG.9309.1	miR-297c-3p
MSTRG.9309.1	miR-297b-3p
MSTRG.9309.1	miR-297a-3p
MSTRG.9309.1	miR-337-3p
MSTRG.9309.1	miR-22-3p
MSTRG.9309.1	miR-153-5p
MSTRG.9309.1	miR-30b-5p
MSTRG.9309.1	miR-3105-3p
MSTRG.9309.1	miR-3110-5p
MSTRG.9309.1	miR-466l-3p
MSTRG.9309.1	miR-452-3p
MSTRG.9309.1	miR-211-5p
MSTRG.9309.1	miR-204-5p
MSTRG.9309.1	miR-93-3p
MSTRG.15980.1	miR-466i-5p
MSTRG.15980.1	miR-466d-5p
MSTRG.15980.1	miR-6903-3p
MSTRG.15980.1	miR-669i
MSTRG.15980.1	miR-7061-5p
MSTRG.15980.1	miR-448-3p
MSTRG.15980.1	miR-7008-5p
MSTRG.15980.1	miR-6967-5p
MSTRG.15980.1	miR-3106-5p
MSTRG.15980.1	miR-471-3p
MSTRG.15980.1	miR-6998-3p
MSTRG.15980.1	miR-6985-5p
MSTRG.15980.1	miR-6950-3p
MSTRG.15980.1	miR-7008-3p
MSTRG.15980.1	miR-6540-3p

MSTRG.15980.1	miR-466l-3p
MSTRG.15980.1	miR-302a-5p
MSTRG.15980.1	miR-7047-5p
MSTRG.15980.1	miR-3079-3p
MSTRG.15980.1	miR-3074-5p
MSTRG.15980.1	miR-218-5p
MSTRG.15980.1	miR-5616-5p
MSTRG.15980.1	miR-455-3p
MSTRG.15980.1	miR-500-5p
MSTRG.15980.1	miR-466p-5p
MSTRG.15980.1	miR-466a-5p
MSTRG.15980.1	miR-1187
MSTRG.18159.11	miR-669c-3p
MSTRG.18159.11	miR-490-5p
MSTRG.18159.11	miR-452-5p
MSTRG.18159.11	miR-429-3p
MSTRG.18159.11	miR-200c-3p
MSTRG.18159.11	miR-200b-3p
MSTRG.18159.11	miR-188-5p
MSTRG.18159.11	miR-22-5p
MSTRG.18159.11	miR-466i-5p
MSTRG.18159.11	miR-466d-5p
MSTRG.18159.11	miR-466f-3p
MSTRG.18159.11	miR-6980-3p
MSTRG.18159.11	miR-101b-3p
MSTRG.18159.11	miR-3084-5p
MSTRG.18159.11	miR-6999-3p
MSTRG.18159.11	miR-7057-3p
MSTRG.18159.11	miR-672-5p
MSTRG.18159.11	miR-6984-3p
MSTRG.18159.11	miR-669e-3p
MSTRG.18159.11	miR-34c-3p
MSTRG.18159.11	miR-34b-3p
MSTRG.18159.11	miR-466l-3p
MSTRG.18159.11	miR-6983-3p
MSTRG.18159.11	miR-452-3p
MSTRG.18159.11	miR-3963
MSTRG.18159.11	miR-124-3p
MSTRG.18159.11	miR-7022-5p
MSTRG.18159.11	miR-7051-5p
MSTRG.18159.11	miR-7224-3p
MSTRG.18159.11	miR-33-3p
MSTRG.18159.11	miR-374c-5p
MSTRG.18159.11	miR-669h-3p

MSTRG.18159.11	miR-362-3p
MSTRG.18159.11	miR-7024-5p
MSTRG.18159.11	miR-7048-3p
MSTRG.18159.11	miR-669i
MSTRG.18159.11	miR-466i-3p
MSTRG.18159.11	miR-145a-3p
MSTRG.18159.11	miR-450b-3p
MSTRG.18159.11	miR-450a-1-3p
MSTRG.18159.11	miR-8097
MSTRG.18159.11	miR-96-5p
MSTRG.18159.11	miR-208b-3p
MSTRG.18159.11	miR-208a-3p
MSTRG.18159.11	miR-8106
MSTRG.18159.11	miR-6941-5p

Table S8. Correlated information of the upregulated lncRNA-associated ceRNA network in the hippocampus of 5×FAD mice.

Upregulated lncRNA	Downregulated miRNA	Upregulated mRNA
ENSMUST00000180635	miR-3085-3p, miR-3064-5p, miR-344g-5p, miR-3069-5p	Wdfy1, Tmed1, Mgll, Fbln2, Cntn2, Abca3, Ash1l, Col1a2, Tmem55b, Pacsin3, Celf2, Cobll1, Fmnl2
ENSMUST00000127786	miR-3085-3p, miR-3064-5p, miR-673-5p, miR-344g-5p, miR-329-3p, miR-466o-3p, miR-466m-3p, miR-186-5p, miR-3069-5p, miR-338-3p, miR-7b-5p, miR-694, miR-466a-3p, miR-466d-3p, miR-466e-3p, miR-467g, miR-466p-3p, miR-466b-3p, miR-466c-3p,	Wdfy1, Tmed1, Mgll, Fbln2, Cntn2, Abca3, Ash1l, Eps8, Islr2, Celf2, Fbxl17, Col1a2, Tmem55b, Pacsin3, Cobll1, Lzts3, Pak6, Tmod3, Clec7a, Fmnl2, Stard3nl, Aplp2, Auts2, Nr3c2, Gng7, Spry1
ENSMUST00000028291	miR-3085-3p, miR-3064-5p, miR-466k	Wdfy1, Tmed1, Mgll, Fbln2, Cntn2, Abca3, Ash1l, Foxp1, Nde1, Clec7a, Fbxl17
ENSMUST00000150127	miR-3085-3p, miR-3064-5p	Wdfy1, Tmed1, Mgll, Fbln2, Cntn2, Abca3, Ash1l
MSTRG.17500.2	miR-3085-3p, miR-3064-5p, miR-673-5p, miR-706, miR-466k, miR-344g-5p, miR-329-3p, miR-466o-3p, miR-466m-3p, miR-186-5p, miR-694, miR-466a-3p, miR-466d-3p, miR-466e-3p, miR-467g, miR-466p-3p, miR-466b-3p, miR-466c-3p	Wdfy1, Tmed1, Mgll, Fbln2, Cntn2, Abca3, Ash1l, Eps8, Islr2, Celf2, Fbxl17, Elovl2, Parp11, Col1a2, Auts2, Akap11, Foxp1, Nde1, Clec7a, Tmem55b, Pacsin3, Cobll1, Lzts3, Pak6, Tmod3, Fmnl2, Nr3c2, Gng7, Spry1
ENSMUST00000197854	miR-673-5p, miR-7b-5p	Eps8, Islr2, Celf2, Fbxl17, Aplp2, Ash1l
MSTRG.17055.1	miR-673-5p, miR-706, miR-466o-3p, miR-466m-3p, miR-338-3p, miR-466p-3p, miR-466b-3p, miR-466c-3p	Eps8, Islr2, Celf2, Fbxl17, Elovl2, Parp11, Col1a2, Abca3, Auts2, Akap11, Lzts3, Cntn2, Clec7a, Stard3nl
MSTRG.1243.32	miR-673-5p, miR-706, miR-329-3p, miR-466o-3p, miR-466m-3p, miR-186-5p, miR-338-3p, miR-7b-5p, miR-466a-3p, miR-466d-3p, miR-466e-3p, miR-467g, miR-466p-3p, miR-466b-3p, miR-466c-3p	Eps8, Islr2, Celf2, Fbxl17, Elovl2, Parp11, Col1a2, Abca3, Auts2, Akap11, Lzts3, Pak6, Tmod3, Clec7a, Fmnl2, Cntn2, Stard3nl, Aplp2, Ash1l, Spry1

MSTRG.10674.1	miR-706, miR-466k, miR-329-3p, miR-466o-3p, miR-466m-3p, miR-7b-5p, miR-466p-3p, miR-466b-3p, miR-466c-3p	Elovl2, Parp11, Col1a2, Abca3, Auts2, Celf2, Akap11, Foxp1, Nde1, Mgll, Clec7a, Fbxl17, Lzts3, Pak6, Tmod3, Aplp2, Ash1l
MSTRG.16327.2	miR-466k	Foxp1, Nde1, Mgll, Clec7a, Fbxl17
MSTRG.6775.12	miR-466k, miR-344g-5p, miR-329-3p, miR-466o-3p, miR-466m-3p, miR-186-5p, miR-3069-5p, miR-338-3p, miR-694, miR-466a-3p, miR-466d-3p, miR-466e-3p, miR-467g, miR-466p-3p, miR-466b-3p, miR-466c-3p	Foxp1, Nde1, Mgll, Clec7a, Fbxl17, Col1a2, Tmem55b, Pacsin3, Celf2, Cobll1, Lzts3, Pak6, Tmod3, Fmnl2, Cntn2, Auts2, Nr3c2, Gng7, Spry1
MSTRG.6530.1	miR-186-5p, miR-338-3p	Fmnl2, Cntn2, Clec7a, Stard3nl
ENSMUST00000152985	miR-3069-5p	Fmnl2, Cntn2
ENSMUST00000153923	miR-694	Auts2, Nr3c2, Gng7

Table S9. Correlated information of the downregulated lncRNA-associated ceRNA network in the hippocampus of 5×FAD mice.

Downregulated lncRNA	Upregulated miRNA	Downregulated mRNA
MSTRG.2483.2	miR-452-3p, miR-136-5p, miR-3110-5p, miR-24-3p, miR-149-5p	Stxbp5l, Dnajc6, Inpp5e, Pik3ip1, 4932438A13Rik, Mapk10, Slc35c2, Cd99l2, Bcorl1, Adarb1, Ddx3y, Rab43, Rragc, Ctnnd1, Ric3, Fnbp1, Ube2i
MSTRG.9309.1	miR-452-3p, miR-5101, miR-669b-5p, miR-466l-3p, miR-466f-3p, miR-3110-5p	Stxbp5l, Dnajc6, Inpp5e, Pik3ip1, 5730455P16Rik, Ilf3, Srrm4, Mapk10, Ctnnd1, Dusp3, Tlk2, Znrf1, Slitrk2, D130043K22Rik, Uggt1, Wdr35, Pcbp2, Ociad2, Lzts3, Lsm14a, Ppargc1a, Adarb1, Ddx3y
MSTRG.18159.11	miR-452-3p, miR-466d-5p, miR-466i-5p, miR-466l-3p, miR-466f-3p, miR-490-5p	Stxbp5l, Dnajc6, Inpp5e, Pik3ip1, Smarca2, Reps2, Slc25a22, Ctdsp2, Fsd1l, Znrf1, Srrm4, Slitrk2, D130043K22Rik, Uggt1, Wdr35, Pcbp2, Ociad2, Lzts3, Lsm14a, Ppargc1a, Aak1, Abat, Fnbp1, Ube2i
ENSMUST00000173081	miR-466d-5p, miR-466i-5p, miR-743a-3p, miR-3113-5p	Stxbp5l, Smarca2, Reps2, Slc25a22, Ctdsp2, Fsd1l, Srrm4
MSTRG.17585.1	miR-466d-5p, miR-466i-5p, miR-3065-5p, miR-5101, miR-669b-5p, miR-3079-3p, miR-136-5p, miR-3474, miR-743a-3p	Stxbp5l, Smarca2, Reps2, Slc25a22, Ctdsp2, Fsd1l, Srrm4, 5730455P16Rik, Ilf3, Mapk10, Ctnnd1, Dusp3, Tlk2, Alg9, Rab43, Aak1, Abat, Inpp5e, Cd99l2, Fnbp1, Sez6l2
MSTRG.15980.1	miR-466d-5p, miR-466i-5p, miR-3079-3p, miR-466l-3p	Stxbp5l, Smarca2, Reps2, Slc25a22, Ctdsp2, Fsd1l, Ilf3, Srrm4, Znrf1, Slitrk2, D130043K22Rik, Uggt1, Wdr35, Pcbp2, Inpp5e
ENSMUST00000184170	miR-3065-5p, miR-669b-5p, miR-3079-3p, miR-15a-5p, miR-743a-3p, miR-24-3p	Stxbp5l, Srrm4, 5730455P16Rik, Ilf3, Uba1, Lzts3, Slc25a22, Ctdsp2, Gnas, Pitpna, Fnbp1, Rab43, Rragc
ENSMUST00000182575	miR-3065-5p, miR-3113-5p	Stxbp5l, Srrm4, Fsd1l
ENSMUST00000197200	miR-5101, miR-669b-5p, miR-136-5p, miR-3474, miR-3113-5p	5730455P16Rik, Ilf3, Srrm4, Mapk10, Ctnnd1, Dusp3, Tlk2, 4932438A13Rik, Slc35c2, Cd99l2, Bcorl1, Alg9, Rab43, Reps2, Aak1, Abat, Inpp5e, Fnbp1, Sez6l2, Fsd1l

ENSMUST00000151020	miR-5101, miR-466l-3p, miR-149-5p, miR-490-5p	5730455P16Rik, Ilf3, Srrm4, Mapk10, Ctnnd1, Dusp3, Tlk2, Znrf1, Slitrk2, D130043K22Rik, Uggt1, Wdr35, Pcbp2, Inpp5e, Ric3, Rragc, Fnbp1, Ube2i, Aak1, Abat
ENSMUST00000162088	miR-15a-5p	Uba1, Lzts3, Slc25a22, Ctdsp2, Gnas, Pitpna, Fnbp1
ENSMUST00000203561	miR-15a-5p	Uba1, Lzts3, Slc25a22, Ctdsp2, Gnas, Pitpna, Fnbp1
ENSMUST00000218286	miR-15a-5p, miR-3474, miR-24-3p, miR-490-5p	Uba1, Lzts3, Slc25a22, Ctdsp2, Gnas, Pitpna, Fnbp1, Alg9, Rab43, Reps2, Aak1, Abat, Inpp5e, Cd99l2, Sez6l2, Rragc, Uggt1, Pcbp2, Ube2i
ENSMUST00000138202	miR-466l-3p, miR-24-3p	Znrf1, Srrm4, Slitrk2, D130043K22Rik, Uggt1, Wdr35, Pcbp2, Inpp5e, Rab43, Rragc
ENSMUST00000199803	miR-466l-3p	Znrf1, Srrm4, Slitrk2, D130043K22Rik, Uggt1, Wdr35, Pcbp2, Inpp5e
ENSMUST00000182642	miR-466f-3p	Znrf1, Ociad2, Lzts3, Lsm14a, Ppargc1a
ENSMUST00000126344	miR-3110-5p, miR-743a-3p	Adarb1, Ddx3y, Srrm4
ENSMUST00000168634	miR-3474	Alg9, Rab43, Reps2, Aak1, Abat, Inpp5e, Cd99l2, Fnbp1, Sez6l2
ENSMUST00000131037	miR-149-5p	Ctnnd1, Ric3, Rragc, Fnbp1, Ube2i
ENSMUST00000119305	miR-3113-5p	Fsd1l

Table S10. Information of the lncRNA-miRNA network in the cerebral cortex and hippocampus of 7-month-old 5×FAD mice.

lncRNA	miRNA targets
ENSMUSG00000098912	miR-380-3p
ENSMUSG00000098912	miR-1933-3p
ENSMUSG00000098912	miR-743b-3p
ENSMUSG00000098912	miR-877-5p
ENSMUSG00000098912	miR-433-3p
ENSMUSG00000098912	miR-669b-5p
ENSMUSG00000098912	miR-1955-5p
ENSMUSG00000098912	miR-214-3p
ENSMUSG00000098912	miR-3079-3p
ENSMUSG00000098912	miR-7080-3p
ENSMUSG00000098912	miR-24-3p
ENSMUSG00000098912	miR-411-5p
ENSMUSG00000098912	miR-153-3p
ENSMUSG00000098912	miR-3060-3p
ENSMUSG00000098912	miR-195a-5p
ENSMUSG00000098912	miR-15b-5p
ENSMUSG00000098912	miR-15a-5p
ENSMUSG00000098912	miR-16-5p
ENSMUSG00000098912	miR-322-5p
ENSMUSG00000098912	miR-497a-5p
ENSMUSG00000098912	miR-195b
ENSMUSG00000098912	miR-3065-5p
ENSMUSG00000098912	miR-3083-5p
ENSMUSG00000098912	miR-193a-3p
ENSMUSG00000098912	miR-193b-3p
ENSMUSG00000098912	miR-18a-5p
ENSMUSG00000098912	miR-3070-5p
ENSMUSG00000098912	miR-6958-3p
ENSMUSG00000098912	miR-488-3p
ENSMUSG00000098912	miR-7010-5p
ENSMUSG00000098912	miR-145b
ENSMUSG00000098912	miR-8120
ENSMUSG00000098912	miR-6914-3p
ENSMUSG00000098912	miR-212-5p
ENSMUSG00000098912	miR-127-5p
ENSMUSG00000098912	miR-7043-3p
ENSMUSG00000098912	miR-7027-5p
ENSMUSG00000098912	miR-151-5p
ENSMUSG00000098912	miR-669p-5p
ENSMUSG00000098912	miR-29c-5p
ENSMUSG00000098912	miR-3058-3p

ENSMUSG00000098912	miR-540-3p
ENSMUST00000127786	miR-669h-5p
ENSMUST00000127786	miR-674-3p
ENSMUST00000127786	miR-7117-5p
ENSMUST00000127786	miR-547-5p
ENSMUST00000127786	miR-221-3p
ENSMUST00000127786	miR-874-3p
ENSMUST00000127786	miR-7688-5p
ENSMUST00000127786	miR-6937-5p
ENSMUST00000127786	miR-574-5p
ENSMUST00000127786	miR-135a-5p
ENSMUST00000127786	miR-3473e
ENSMUST00000127786	miR-582-5p
ENSMUST00000127786	miR-215-5p
ENSMUST00000127786	miR-7674-3p
ENSMUST00000127786	miR-344g-5p
ENSMUST00000127786	miR-26b-3p
ENSMUST00000127786	miR-344-3p
ENSMUST00000127786	miR-6240
ENSMUST00000127786	miR-3064-5p
ENSMUST00000127786	miR-6979-3p
ENSMUST00000127786	miR-129b-3p
ENSMUST00000127786	miR-7021-5p
ENSMUST00000127786	miR-153-3p
ENSMUST00000127786	miR-30e-5p
ENSMUST00000127786	miR-146a-5p
ENSMUST00000127786	miR-344d-1-5p
ENSMUST00000127786	miR-143-3p
ENSMUST00000127786	miR-7000-3p
ENSMUST00000127786	miR-106b-5p
ENSMUST00000127786	miR-3095-5p
ENSMUST00000127786	miR-669l-5p
ENSMUST00000127786	miR-493-5p
ENSMUST00000127786	miR-673-5p
ENSMUST00000127786	miR-3081-3p
ENSMUST00000127786	miR-1934-5p
ENSMUST00000127786	miR-31-5p
ENSMUST00000127786	miR-1927
ENSMUST00000127786	miR-6537-3p
ENSMUST00000127786	miR-148a-5p
ENSMUST00000127786	miR-3067-5p
ENSMUST00000127786	miR-26a-5p
ENSMUST00000127786	miR-181b-1-3p
ENSMUST00000127786	miR-133a-3p

ENSMUST00000127786	miR-29b-3p
ENSMUST00000127786	miR-302d-3p
ENSMUST00000127786	miR-3112-5p
ENSMUST00000127786	miR-467a-3p
ENSMUST00000127786	miR-3105-3p
ENSMUST00000127786	miR-130a-5p
ENSMUST00000127786	miR-3097-5p
ENSMUST00000127786	miR-137-5p
ENSMUST00000127786	miR-1933-3p
ENSMUST00000127786	miR-1946a
ENSMUST00000127786	miR-682
ENSMUST00000127786	miR-7037-5p
ENSMUST00000127786	miR-704
ENSMUST00000127786	miR-411-3p
ENSMUST00000127786	miR-7016-5p
ENSMUST00000127786	miR-6999-5p
ENSMUST00000127786	miR-6927-3p
ENSMUST00000127786	miR-503-3p
ENSMUST00000127786	miR-30f
ENSMUST00000127786	miR-3098-3p
ENSMUST00000127786	miR-125b-2-3p
ENSMUST00000127786	miR-322-5p
ENSMUST00000127786	miR-500-5p
ENSMUST00000127786	miR-6933-3p
ENSMUST00000127786	miR-7116-3p
ENSMUST00000127786	miR-301b-3p
ENSMUST00000127786	miR-30b-5p
ENSMUST00000127786	miR-344-5p
ENSMUST00000127786	miR-345-5p
ENSMUST00000127786	miR-7115-3p
ENSMUST00000127786	miR-690
ENSMUST00000127786	miR-3102-3p
ENSMUST00000127786	miR-299a-5p
ENSMUST00000127786	miR-3074-1-3p
ENSMUST00000127786	miR-505-5p
ENSMUST00000127786	miR-6395
ENSMUST00000127786	miR-494-3p
ENSMUST00000127786	miR-223-5p
ENSMUST00000127786	miR-488-3p
ENSMUST00000127786	miR-152-5p
ENSMUST00000127786	miR-7019-3p
ENSMUST00000127786	miR-6516-3p
ENSMUST00000127786	miR-6982-5p
ENSMUST00000127786	miR-1946b

ENSMUST00000127786	miR-185-3p
ENSMUST00000127786	miR-3080-3p
ENSMUST00000127786	miR-412-3p
ENSMUST00000127786	miR-501-5p
ENSMUST00000127786	miR-743b-3p
ENSMUST00000127786	miR-1843a-5p
ENSMUST00000127786	miR-337-3p
ENSMUST00000127786	miR-6975-3p
ENSMUST00000127786	miR-6925-5p
ENSMUST00000127786	miR-26a-2-3p
ENSMUST00000127786	miR-669i
ENSMUST00000127786	miR-134-5p
ENSMUST00000127786	miR-669k-5p
ENSMUST00000127786	miR-411-5p
ENSMUST00000127786	miR-191-5p
ENSMUST00000127786	miR-183-3p
ENSMUST00000127786	miR-20a-5p
ENSMUST00000127786	miR-7687-3p
ENSMUST00000127786	miR-124-3p
ENSMUST00000127786	miR-7651-5p
ENSMUST00000127786	miR-3082-5p
ENSMUST00000127786	let-7f-2-3p
ENSMUST00000127786	miR-7663-5p
ENSMUST00000127786	miR-6947-5p
ENSMUST00000127786	miR-216b-5p
ENSMUST00000127786	miR-3103-3p
ENSMUST00000127786	miR-344d-3-5p
ENSMUST00000127786	miR-1930-5p
ENSMUST00000127786	miR-7662-3p
ENSMUST00000127786	miR-296-5p
ENSMUST00000127786	miR-497a-5p
ENSMUST00000127786	miR-210-5p
ENSMUST00000127786	miR-6911-5p
ENSMUST00000127786	miR-665-5p
ENSMUST00000127786	miR-30e-3p
ENSMUST00000127786	miR-500-3p
ENSMUST00000127786	miR-1912-3p
ENSMUST00000127786	miR-224-5p
ENSMUST00000127786	miR-101a-3p
ENSMUST00000127786	miR-551b-5p
ENSMUST00000127786	miR-7031-5p
ENSMUST00000127786	miR-6982-3p
ENSMUST00000127786	miR-5623-5p
ENSMUST00000127786	miR-8118

ENSMUST00000127786	miR-3084-5p
ENSMUST00000127786	miR-592-5p
ENSMUST00000127786	miR-195b
ENSMUST00000127786	miR-7024-3p
ENSMUST00000127786	miR-96-5p
ENSMUST00000127786	miR-1298-5p
ENSMUST00000127786	miR-486b-3p
ENSMUST00000127786	miR-7b-5p
ENSMUST00000127786	miR-1949
ENSMUST00000127786	miR-693-3p
ENSMUST00000127786	miR-3473a
ENSMUST00000127786	miR-3066-5p
ENSMUST00000127786	miR-664-3p
ENSMUST00000127786	miR-384-5p
ENSMUST00000127786	miR-351-5p
ENSMUST00000127786	miR-6921-3p
ENSMUST00000127786	miR-471-5p
ENSMUST00000127786	miR-7032-3p
ENSMUST00000127786	miR-5710
ENSMUST00000127786	miR-3474
ENSMUST00000127786	miR-379-3p
ENSMUST00000127786	miR-381-3p
ENSMUST00000127786	miR-486a-5p
ENSMUST00000127786	miR-7054-5p
ENSMUST00000127786	miR-493-3p
ENSMUST00000127786	miR-6481
ENSMUST00000127786	let-7a-5p
ENSMUST00000127786	miR-181b-5p
ENSMUST00000127786	miR-344c-5p
ENSMUST00000127786	miR-431-3p
ENSMUST00000127786	miR-6913-5p
ENSMUST00000127786	miR-1931
ENSMUST00000127786	miR-346-3p
ENSMUST00000127786	miR-23a-3p
ENSMUST00000127786	miR-466c-3p
ENSMUST00000127786	miR-7675-3p
ENSMUST00000127786	miR-6911-3p
ENSMUST00000127786	miR-7066-3p
ENSMUST00000127786	miR-1231-5p
ENSMUST00000127786	miR-3963
ENSMUST00000127786	miR-409-5p
ENSMUST00000127786	miR-6928-5p
ENSMUST00000127786	miR-345-3p
ENSMUST00000127786	miR-362-3p

ENSMUST00000127786	miR-3074-5p
ENSMUST00000127786	miR-1191a
ENSMUST00000127786	miR-9-5p
ENSMUST00000127786	miR-3970
ENSMUST00000127786	miR-1197-3p
ENSMUST00000127786	miR-7087-5p
ENSMUST00000127786	miR-29c-3p
ENSMUST00000127786	miR-155-5p
ENSMUST00000127786	miR-6976-5p
ENSMUST00000127786	miR-669c-3p
ENSMUST00000127786	miR-183-5p
ENSMUST00000127786	let-7g-5p
ENSMUST00000127786	miR-504-5p
ENSMUST00000127786	miR-3099-3p
ENSMUST00000127786	miR-3085-3p
ENSMUST00000127786	miR-7066-5p
ENSMUST00000127786	miR-6906-5p
ENSMUST00000127786	miR-3089-5p
ENSMUST00000127786	miR-350-5p
ENSMUST00000127786	miR-3080-5p
ENSMUST00000127786	miR-137-3p
ENSMUST00000127786	miR-199a-3p
ENSMUST00000127786	miR-3473d
ENSMUST00000127786	miR-449a-5p
ENSMUST00000127786	miR-135b-5p
ENSMUST00000127786	miR-410-5p
ENSMUST00000127786	miR-146b-3p
ENSMUST00000127786	miR-324-3p
ENSMUST00000127786	miR-466b-3p
ENSMUST00000127786	miR-302b-3p
ENSMUST00000127786	miR-3473g
ENSMUST00000127786	miR-135a-2-3p
ENSMUST00000127786	miR-374b-5p
ENSMUST00000127786	miR-1b-5p
ENSMUST00000127786	miR-34a-3p
ENSMUST00000127786	miR-98-5p
ENSMUST00000127786	miR-741-3p
ENSMUST00000127786	miR-7042-5p
ENSMUST00000127786	miR-150-5p
ENSMUST00000127786	let-7f-5p
ENSMUST00000127786	miR-539-5p
ENSMUST00000127786	miR-3109-5p
ENSMUST00000127786	miR-34a-5p
ENSMUST00000127786	miR-6902-3p

ENSMUST00000127786	miR-1969
ENSMUST00000127786	miR-8111
ENSMUST00000127786	miR-1264-3p
ENSMUST00000127786	miR-669a-5p
ENSMUST00000127786	miR-34c-5p
ENSMUST00000127786	miR-200c-3p
ENSMUST00000127786	miR-5624-3p
ENSMUST00000127786	miR-466i-3p
ENSMUST00000127786	miR-141-3p
ENSMUST00000127786	miR-3064-3p
ENSMUST00000127786	miR-30c-1-3p
ENSMUST00000127786	miR-181b-2-3p
ENSMUST00000127786	miR-125a-5p
ENSMUST00000127786	miR-200b-3p
ENSMUST00000127786	miR-344d-3p
ENSMUST00000127786	miR-361-5p
ENSMUST00000127786	miR-216a-5p
ENSMUST00000127786	miR-221-5p
ENSMUST00000127786	miR-483-5p
ENSMUST00000127786	miR-3093-3p
ENSMUST00000127786	miR-7646-5p
ENSMUST00000127786	miR-101b-3p
ENSMUST00000127786	miR-30a-3p
ENSMUST00000127786	miR-33-5p
ENSMUST00000127786	miR-6946-5p
ENSMUST00000127786	miR-449b
ENSMUST00000127786	miR-16-5p
ENSMUST00000127786	miR-127-3p
ENSMUST00000127786	miR-140-5p
ENSMUST00000127786	miR-7664-5p
ENSMUST00000127786	miR-222-5p
ENSMUST00000127786	miR-30a-5p
ENSMUST00000127786	miR-872-5p
ENSMUST00000127786	miR-93-5p
ENSMUST00000127786	miR-155-3p
ENSMUST00000127786	miR-145b
ENSMUST00000127786	miR-7013-3p
ENSMUST00000127786	miR-466l-3p
ENSMUST00000127786	miR-466e-3p
ENSMUST00000127786	miR-344c-3p
ENSMUST00000127786	miR-3086-3p
ENSMUST00000127786	miR-15a-3p
ENSMUST00000127786	miR-665-3p
ENSMUST00000127786	miR-3473b

ENSMUST00000127786	miR-146b-5p
ENSMUST00000127786	miR-1948-5p
ENSMUST00000127786	let-7i-5p
ENSMUST00000127786	miR-378b
ENSMUST00000127786	miR-664-5p
ENSMUST00000127786	miR-6910-3p
ENSMUST00000127786	miR-7061-5p
ENSMUST00000127786	miR-465c-5p
ENSMUST00000127786	miR-200a-3p
ENSMUST00000127786	miR-6991-5p
ENSMUST00000127786	miR-329-3p
ENSMUST00000127786	miR-186-5p
ENSMUST00000127786	miR-6540-5p
ENSMUST00000127786	miR-195a-5p
ENSMUST00000127786	miR-320-3p
ENSMUST00000127786	miR-491-3p
ENSMUST00000127786	miR-410-3p
ENSMUST00000127786	miR-330-3p
ENSMUST00000127786	miR-298-5p
ENSMUST00000127786	miR-10a-5p
ENSMUST00000127786	miR-10b-5p
ENSMUST00000127786	miR-760-5p
ENSMUST00000127786	miR-7b-3p
ENSMUST00000127786	miR-296-3p
ENSMUST00000127786	miR-335-3p
ENSMUST00000127786	miR-19b-3p
ENSMUST00000127786	miR-5101
ENSMUST00000127786	miR-3087-3p
ENSMUST00000127786	miR-702-5p
ENSMUST00000127786	miR-127-5p
ENSMUST00000127786	miR-297a-3p
ENSMUST00000127786	miR-125b-5p
ENSMUST00000127786	miR-17-5p
ENSMUST00000127786	miR-19a-3p
ENSMUST00000127786	miR-3113-5p
ENSMUST00000127786	miR-3470b
ENSMUST00000127786	miR-300-3p
ENSMUST00000127786	miR-138-5p
ENSMUST00000127786	miR-124-5p
ENSMUST00000127786	miR-1224-5p
ENSMUST00000127786	miR-669b-5p
ENSMUST00000127786	miR-384-3p
ENSMUST00000127786	miR-1955-3p
ENSMUST00000127786	miR-425-5p

ENSMUST00000127786	miR-6937-3p
ENSMUST00000127786	miR-362-5p
ENSMUST00000127786	miR-7116-5p
ENSMUST00000127786	miR-222-3p
ENSMUST00000127786	miR-3086-5p
ENSMUST00000127786	miR-423-5p
ENSMUST00000127786	miR-3095-3p
ENSMUST00000127786	miR-3079-3p
ENSMUST00000127786	miR-6539
ENSMUST00000127786	miR-669p-3p
ENSMUST00000127786	miR-708-3p
ENSMUST00000127786	miR-670-5p
ENSMUST00000127786	miR-466o-3p
ENSMUST00000127786	miR-5617-5p
ENSMUST00000127786	miR-669e-3p
ENSMUST00000127786	miR-7224-3p
ENSMUST00000127786	miR-6919-5p
ENSMUST00000127786	miR-489-3p
ENSMUST00000127786	miR-145a-5p
ENSMUST00000127786	miR-338-5p
ENSMUST00000127786	miR-7037-3p
ENSMUST00000127786	miR-10a-3p
ENSMUST00000127786	miR-7085-3p
ENSMUST00000127786	miR-6970-5p
ENSMUST00000127786	miR-107-5p
ENSMUST00000127786	miR-679-3p
ENSMUST00000127786	let-7e-5p
ENSMUST00000127786	miR-1224-3p
ENSMUST00000127786	miR-541-3p
ENSMUST00000127786	miR-344d-2-5p
ENSMUST00000127786	miR-219a-5p
ENSMUST00000127786	miR-9-3p
ENSMUST00000127786	miR-27b-3p
ENSMUST00000127786	miR-344e-3p
ENSMUST00000127786	miR-3102-5p.2-5p
ENSMUST00000127786	miR-135a-1-3p
ENSMUST00000127786	miR-186-3p
ENSMUST00000127786	miR-6896-5p
ENSMUST00000127786	miR-1198-5p
ENSMUST00000127786	miR-483-3p
ENSMUST00000127786	miR-130b-3p
ENSMUST00000127786	miR-212-3p
ENSMUST00000127786	miR-7039-5p
ENSMUST00000127786	miR-15b-5p

ENSMUST00000127786	miR-338-3p
ENSMUST00000127786	miR-1298-3p
ENSMUST00000127786	miR-466n-3p
ENSMUST00000127786	miR-128-3p
ENSMUST00000127786	miR-382-3p
ENSMUST00000127786	miR-219b-3p
ENSMUST00000127786	miR-470-5p
ENSMUST00000127786	miR-29a-3p
ENSMUST00000127786	miR-669f-3p
ENSMUST00000127786	miR-23b-3p
ENSMUST00000127786	miR-543-3p
ENSMUST00000127786	miR-7224-5p
ENSMUST00000127786	miR-27a-3p
ENSMUST00000127786	miR-450a-2-3p
ENSMUST00000127786	miR-7665-5p
ENSMUST00000127786	miR-3475-3p
ENSMUST00000127786	miR-429-3p
ENSMUST00000127786	miR-449c-5p
ENSMUST00000127786	miR-7661-3p
ENSMUST00000127786	miR-181d-5p
ENSMUST00000127786	miR-192-5p
ENSMUST00000127786	miR-7226-3p
ENSMUST00000127786	miR-133b-3p
ENSMUST00000127786	miR-3065-5p
ENSMUST00000127786	miR-5621-3p
ENSMUST00000127786	miR-6896-3p
ENSMUST00000127786	miR-181c-5p
ENSMUST00000127786	miR-20b-5p
ENSMUST00000127786	miR-669p-5p
ENSMUST00000127786	miR-880-3p
ENSMUST00000127786	miR-144-3p
ENSMUST00000127786	miR-499-5p
ENSMUST00000127786	miR-24-3p
ENSMUST00000127786	miR-329-5p
ENSMUST00000127786	miR-544-3p
ENSMUST00000127786	miR-1188-5p
ENSMUST00000127786	miR-6985-3p
ENSMUST00000127786	miR-3058-3p
ENSMUST00000127786	miR-7092-3p
ENSMUST00000127786	miR-543-5p
ENSMUST00000127786	miR-148b-3p
ENSMUST00000127786	miR-377-3p
ENSMUST00000127786	miR-214-3p
ENSMUST00000127786	miR-7064-5p

ENSMUST00000127786	miR-450a-1-3p
ENSMUST00000127786	miR-540-5p
ENSMUST00000127786	miR-106a-5p
ENSMUST00000127786	miR-5615-5p
ENSMUST00000127786	miR-5615-3p
ENSMUST00000127786	miR-201-5p
ENSMUST00000127786	miR-26b-5p
ENSMUST00000127786	miR-130b-5p
ENSMUST00000127786	miR-466m-3p
ENSMUST00000127786	miR-326-3p
ENSMUST00000127786	let-7b-5p
ENSMUST00000127786	miR-879-5p
ENSMUST00000127786	miR-466p-3p
ENSMUST00000127786	miR-152-3p
ENSMUST00000127786	miR-3057-5p
ENSMUST00000127786	miR-6903-3p
ENSMUST00000127786	miR-466q
ENSMUST00000127786	miR-3094-5p
ENSMUST00000127786	miR-6971-3p
ENSMUST00000127786	miR-187-5p
ENSMUST00000127786	miR-328-3p
ENSMUST00000127786	miR-224-3p
ENSMUST00000127786	miR-135b-3p
ENSMUST00000127786	miR-3472
ENSMUST00000127786	miR-669a-3-3p
ENSMUST00000127786	miR-694
ENSMUST00000127786	miR-1981-5p
ENSMUST00000127786	miR-7085-5p
ENSMUST00000127786	miR-669o-3p
ENSMUST00000127786	miR-758-3p
ENSMUST00000127786	miR-199b-3p
ENSMUST00000127786	miR-153-5p
ENSMUST00000127786	miR-107-3p
ENSMUST00000127786	miR-669b-3p
ENSMUST00000127786	miR-378a-3p
ENSMUST00000127786	miR-22-3p
ENSMUST00000127786	miR-7022-5p
ENSMUST00000127786	miR-7080-3p
ENSMUST00000127786	miR-3967
ENSMUST00000127786	miR-6945-3p
ENSMUST00000127786	miR-505-3p
ENSMUST00000127786	miR-7a-2-3p
ENSMUST00000127786	miR-7015-3p
ENSMUST00000127786	miR-6984-5p

ENSMUST00000127786	miR-669a-3p
ENSMUST00000127786	miR-7240-5p
ENSMUST00000127786	miR-669f-5p
ENSMUST00000127786	miR-764-5p
ENSMUST00000127786	miR-3098-5p
ENSMUST00000127786	miR-672-3p
ENSMUST00000127786	miR-501-3p
ENSMUST00000127786	let-7d-5p
ENSMUST00000127786	miR-148a-3p
ENSMUST00000127786	miR-3059-5p
ENSMUST00000127786	miR-7004-5p
ENSMUST00000127786	miR-188-5p
ENSMUST00000127786	miR-342-3p
ENSMUST00000127786	miR-6916-3p
ENSMUST00000127786	miR-145a-3p
ENSMUST00000127786	miR-421-3p
ENSMUST00000127786	miR-350-3p
ENSMUST00000127786	miR-148b-5p
ENSMUST00000127786	miR-376c-3p
ENSMUST00000127786	miR-8106
ENSMUST00000127786	miR-3069-5p
ENSMUST00000127786	miR-7a-5p
ENSMUST00000127786	miR-378c
ENSMUST00000127786	miR-448-3p
ENSMUST00000127786	miR-6999-3p
ENSMUST00000127786	miR-330-5p
ENSMUST00000127786	miR-6914-3p
ENSMUST00000127786	miR-130a-3p
ENSMUST00000127786	miR-3091-3p
ENSMUST00000127786	miR-455-3p
ENSMUST00000127786	miR-6905-3p
ENSMUST00000127786	miR-6918-5p
ENSMUST00000127786	miR-103-3p
ENSMUST00000127786	miR-466f-3p
ENSMUST00000127786	miR-301a-3p
ENSMUST00000127786	miR-7013-5p
ENSMUST00000127786	miR-1258-3p
ENSMUST00000127786	miR-129-2-3p
ENSMUST00000127786	miR-669e-5p
ENSMUST00000127786	miR-30d-3p
ENSMUST00000127786	miR-539-3p
ENSMUST00000127786	miR-140-3p
ENSMUST00000127786	miR-6997-5p
ENSMUST00000127786	miR-185-5p

ENSMUST00000127786	miR-7236-3p
ENSMUST00000127786	miR-34b-5p
ENSMUST00000127786	miR-217-5p
ENSMUST00000127786	miR-3060-3p
ENSMUST00000127786	miR-297c-3p
ENSMUST00000127786	miR-3083-5p
ENSMUST00000127786	miR-344b-3p
ENSMUST00000127786	miR-203-3p
ENSMUST00000127786	miR-30d-5p
ENSMUST00000127786	miR-674-5p
ENSMUST00000127786	miR-300-5p
ENSMUST00000127786	miR-5709-5p
ENSMUST00000127786	miR-15a-5p
ENSMUST00000127786	miR-20a-3p
ENSMUST00000127786	miR-486a-3p
ENSMUST00000127786	miR-30c-5p
ENSMUST00000127786	miR-497b
ENSMUST00000127786	miR-7077-5p
ENSMUST00000127786	miR-8114
ENSMUST00000127786	miR-132-3p
ENSMUST00000127786	miR-511-5p
ENSMUST00000127786	miR-1943-5p
ENSMUST00000127786	let-7k
ENSMUST00000127786	miR-1961
ENSMUST00000127786	miR-181a-5p
ENSMUST00000127786	miR-5620-3p
ENSMUST00000127786	miR-7038-3p
ENSMUST00000127786	miR-466a-3p
ENSMUST00000127786	miR-383-3p
ENSMUST00000127786	miR-1198-3p
ENSMUST00000127786	miR-6899-3p
ENSMUST00000127786	miR-1912-5p
ENSMUST00000127786	miR-6516-5p
ENSMUST00000127786	miR-465a-5p
ENSMUST00000127786	miR-203-5p
ENSMUST00000127786	let-7c-5p
ENSMUST00000127786	miR-322-3p
ENSMUST00000127786	miR-615-3p
ENSMUST00000127786	miR-666-3p
ENSMUST00000127786	miR-3094-3p
ENSMUST00000127786	miR-667-3p
ENSMUST00000127786	miR-1941-5p
ENSMUST00000127786	miR-466d-3p
ENSMUST00000127786	miR-6967-5p

ENSMUST00000127786	miR-92a-1-5p
ENSMUST00000127786	miR-1983
ENSMUST00000127786	miR-204-3p
ENSMUST00000127786	miR-486b-5p
ENSMUST00000127786	miR-6964-3p
ENSMUST00000127786	miR-192-3p
ENSMUST00000127786	miR-3058-5p
ENSMUST00000127786	miR-297b-3p
ENSMUST00000127786	miR-494-5p
ENSMUST00000127786	miR-7047-3p
ENSMUST00000127786	miR-331-3p
ENSMUST00000127786	miR-3062-5p
ENSMUST00000127786	miR-448-5p
ENSMUST00000127786	miR-340-5p
ENSMUST00000127786	miR-3078-5p
ENSMUST00000127786	miR-129-1-3p
ENSMUST00000127786	miR-30c-2-3p
ENSMUST00000127786	miR-503-5p
ENSMUST00000127786	miR-450b-3p
ENSMUST00000127786	miR-1191b-5p
MSTRG.11359.1	miR-7667-3p
MSTRG.11359.1	miR-365-2-5p
MSTRG.11359.1	miR-365-1-5p
MSTRG.11359.1	miR-3093-3p
MSTRG.11359.1	miR-6994-3p
MSTRG.17500.2	miR-6900-3p
MSTRG.17500.2	miR-195a-3p
MSTRG.17500.2	miR-346-3p
MSTRG.17500.2	miR-532-5p
MSTRG.17500.2	miR-466q
MSTRG.17500.2	miR-669h-5p
MSTRG.17500.2	miR-3572-5p
MSTRG.17500.2	miR-7224-5p
MSTRG.17500.2	miR-7663-3p
MSTRG.17500.2	miR-466n-3p
MSTRG.17500.2	miR-3106-5p
MSTRG.17500.2	miR-694
MSTRG.17500.2	miR-7667-3p
MSTRG.17500.2	miR-150-5p
MSTRG.17500.2	miR-879-5p
MSTRG.17500.2	miR-300-5p
MSTRG.17500.2	miR-338-5p
MSTRG.17500.2	miR-301b-5p
MSTRG.17500.2	miR-301a-5p

MSTRG.17500.2	miR-145b
MSTRG.17500.2	miR-145a-5p
MSTRG.17500.2	miR-741-3p
MSTRG.17500.2	miR-3094-5p
MSTRG.17500.2	miR-133a-5p
MSTRG.17500.2	miR-3065-5p
MSTRG.17500.2	miR-450a-2-3p
MSTRG.17500.2	miR-7031-5p
MSTRG.17500.2	miR-323-5p
MSTRG.17500.2	miR-3074-5p
MSTRG.17500.2	miR-1249-5p
MSTRG.17500.2	miR-7052-3p
MSTRG.17500.2	miR-6951-5p
MSTRG.17500.2	miR-466p-3p
MSTRG.17500.2	miR-466c-3p
MSTRG.17500.2	miR-466b-3p
MSTRG.17500.2	miR-7063-5p
MSTRG.17500.2	miR-362-3p
MSTRG.17500.2	miR-329-3p
MSTRG.17500.2	miR-6958-3p
MSTRG.17500.2	miR-344i
MSTRG.17500.2	miR-1929-3p
MSTRG.17500.2	miR-1193-3p
MSTRG.17500.2	miR-7084-5p
MSTRG.17500.2	miR-466f-3p
MSTRG.17500.2	miR-7015-3p
MSTRG.17500.2	miR-493-5p
MSTRG.17500.2	miR-134-3p
MSTRG.17500.2	miR-7093-3p
MSTRG.17500.2	miR-6994-5p
MSTRG.17500.2	miR-551b-5p
MSTRG.17500.2	miR-696
MSTRG.17500.2	miR-7077-3p
MSTRG.17500.2	miR-433-3p
MSTRG.17500.2	miR-1943-5p
MSTRG.17500.2	miR-5615-5p
MSTRG.17500.2	miR-3064-3p
MSTRG.17500.2	miR-330-5p
MSTRG.17500.2	miR-383-3p
MSTRG.17500.2	miR-7065-3p
MSTRG.17500.2	miR-491-3p
MSTRG.17500.2	miR-7048-3p
MSTRG.17500.2	miR-6985-5p
MSTRG.17500.2	miR-299b-3p

MSTRG.17500.2	miR-299a-3p
MSTRG.17500.2	miR-544-5p
MSTRG.17500.2	miR-706
MSTRG.17500.2	miR-199b-5p
MSTRG.17500.2	miR-199a-5p
MSTRG.17500.2	miR-222-5p
MSTRG.17500.2	miR-1948-5p
MSTRG.17500.2	miR-3085-3p
MSTRG.17500.2	miR-3064-5p
MSTRG.17500.2	miR-7064-5p
MSTRG.17500.2	miR-6985-3p
MSTRG.17500.2	miR-130b-5p
MSTRG.17500.2	miR-935
MSTRG.17500.2	miR-224-5p
MSTRG.17500.2	miR-3088-3p
MSTRG.17500.2	miR-3971
MSTRG.17500.2	miR-7052-5p
MSTRG.17500.2	miR-300-3p
MSTRG.17500.2	miR-1960
MSTRG.17500.2	miR-152-5p
MSTRG.17500.2	miR-7047-3p
MSTRG.17500.2	miR-195b
MSTRG.17500.2	miR-195a-5p
MSTRG.17500.2	miR-16-5p
MSTRG.17500.2	miR-15b-5p
MSTRG.17500.2	miR-15a-5p
MSTRG.17500.2	miR-326-3p
MSTRG.17500.2	miR-98-5p
MSTRG.17500.2	let-7k
MSTRG.17500.2	let-7i-5p
MSTRG.17500.2	let-7g-5p
MSTRG.17500.2	let-7f-5p
MSTRG.17500.2	let-7e-5p
MSTRG.17500.2	let-7c-5p
MSTRG.17500.2	let-7b-5p
MSTRG.17500.2	let-7a-5p
MSTRG.17500.2	miR-152-3p
MSTRG.17500.2	miR-148b-3p
MSTRG.17500.2	miR-148a-3p
MSTRG.17500.2	miR-6999-3p
MSTRG.17500.2	miR-669o-3p
MSTRG.17500.2	miR-669a-3p
MSTRG.17500.2	miR-669a-3-3p
MSTRG.17500.2	miR-7685-5p

MSTRG.17500.2	miR-3094-3p
MSTRG.17500.2	miR-3095-5p
MSTRG.17500.2	miR-1941-3p
MSTRG.17500.2	miR-1983
MSTRG.17500.2	miR-6912-5p
MSTRG.17500.2	miR-488-5p
MSTRG.17500.2	miR-499-5p
MSTRG.17500.2	miR-101a-3p
MSTRG.17500.2	miR-212-3p
MSTRG.17500.2	miR-132-3p
MSTRG.17500.2	miR-497a-5p
MSTRG.17500.2	miR-322-5p
MSTRG.17500.2	miR-412-3p
MSTRG.17500.2	miR-6953-3p
MSTRG.17500.2	miR-7085-3p
MSTRG.17500.2	miR-669h-3p
MSTRG.17500.2	miR-877-5p
MSTRG.17500.2	miR-8106
MSTRG.17500.2	miR-3066-3p
MSTRG.17500.2	miR-142a-5p
MSTRG.17500.2	miR-3966
MSTRG.17500.2	miR-8107
MSTRG.17500.2	miR-365-3p
MSTRG.17500.2	miR-7077-5p
MSTRG.17500.2	miR-7689-3p
MSTRG.17500.2	miR-6901-3p
MSTRG.17500.2	miR-3084-3p
MSTRG.17500.2	miR-698-5p
MSTRG.17500.2	miR-6948-3p
MSTRG.17500.2	miR-7016-5p
MSTRG.17500.2	miR-6967-5p
MSTRG.17500.2	miR-324-3p
MSTRG.17500.2	miR-384-3p
MSTRG.17500.2	miR-3100-5p
MSTRG.17500.2	miR-377-5p
MSTRG.17500.2	miR-669b-5p
MSTRG.17500.2	let-7d-5p
MSTRG.17500.2	miR-7055-3p
MSTRG.17500.2	miR-6540-3p
MSTRG.17500.2	miR-370-5p
MSTRG.17500.2	miR-1b-3p
MSTRG.17500.2	miR-32-3p
MSTRG.17500.2	miR-5121
MSTRG.17500.2	miR-214-3p

MSTRG.17500.2	miR-6909-5p
MSTRG.17500.2	miR-770-3p
MSTRG.17500.2	miR-7656-5p
MSTRG.17500.2	miR-6769b-5p
MSTRG.17500.2	miR-30e-3p
MSTRG.17500.2	miR-30d-3p
MSTRG.17500.2	miR-30a-3p
MSTRG.17500.2	let-7j
MSTRG.17500.2	miR-6962-5p
MSTRG.17500.2	miR-504-5p
MSTRG.17500.2	miR-673-5p
MSTRG.17500.2	miR-3057-3p
MSTRG.17500.2	miR-5615-3p
MSTRG.17500.2	miR-709
MSTRG.17500.2	miR-873a-3p
MSTRG.17500.2	miR-3108-5p
MSTRG.17500.2	miR-6998-5p
MSTRG.17500.2	miR-346-5p
MSTRG.17500.2	miR-361-3p
MSTRG.17500.2	miR-7661-3p
MSTRG.17500.2	miR-142b
MSTRG.17500.2	miR-3099-3p
MSTRG.17500.2	miR-674-5p
MSTRG.17500.2	miR-1912-5p
MSTRG.17500.2	miR-701-3p
MSTRG.17500.2	miR-186-5p
MSTRG.17500.2	miR-5620-3p
MSTRG.17500.2	miR-7674-5p
MSTRG.17500.2	miR-5710
MSTRG.17500.2	miR-7024-3p
MSTRG.17500.2	miR-6964-3p
MSTRG.17500.2	miR-342-3p
MSTRG.17500.2	miR-344g-5p
MSTRG.17500.2	miR-344d-3-5p
MSTRG.17500.2	miR-344d-1-5p
MSTRG.17500.2	miR-344c-5p
MSTRG.17500.2	miR-344-5p
MSTRG.17500.2	miR-6944-5p
MSTRG.17500.2	miR-466i-3p
MSTRG.17500.2	miR-3087-3p
MSTRG.17500.2	miR-297b-5p
MSTRG.17500.2	miR-181d-3p
MSTRG.17500.2	miR-6941-5p
MSTRG.17500.2	miR-7649-3p

MSTRG.17500.2	miR-219a-5p
MSTRG.17500.2	miR-344d-2-5p
MSTRG.17500.2	miR-6937-3p
MSTRG.17500.2	miR-211-5p
MSTRG.17500.2	miR-7231-5p
MSTRG.17500.2	miR-7000-3p
MSTRG.17500.2	miR-6516-3p
MSTRG.17500.2	miR-21b
MSTRG.17500.2	miR-5626-3p
MSTRG.17500.2	miR-350-3p
MSTRG.17500.2	miR-377-3p
MSTRG.17500.2	miR-297c-5p
MSTRG.17500.2	miR-297a-5p
MSTRG.17500.2	miR-363-5p
MSTRG.17500.2	miR-204-5p
MSTRG.17500.2	miR-339-5p
MSTRG.17500.2	miR-7008-3p
MSTRG.17500.2	miR-3076-5p
MSTRG.17500.2	miR-1964-5p
MSTRG.17500.2	miR-1961
MSTRG.17500.2	miR-7646-5p
MSTRG.17500.2	miR-5619-3p
MSTRG.17500.2	miR-6946-3p
MSTRG.17500.2	miR-182-5p
MSTRG.17500.2	miR-3078-3p
MSTRG.17500.2	miR-3099-5p
MSTRG.17500.2	miR-7026-5p
MSTRG.17500.2	miR-6952-3p
MSTRG.17500.2	miR-1839-5p
MSTRG.17500.2	miR-185-3p
MSTRG.17500.2	miR-1231-5p
MSTRG.17500.2	miR-7243-3p
MSTRG.17500.2	miR-433-5p
MSTRG.17500.2	miR-210-5p
MSTRG.17500.2	miR-664-5p
MSTRG.17500.2	miR-1258-5p
MSTRG.17500.2	miR-204-3p
MSTRG.17500.2	miR-145a-3p
MSTRG.17500.2	miR-1954
MSTRG.17500.2	miR-1194
MSTRG.17500.2	miR-7214-5p
MSTRG.17500.2	miR-1968-5p
MSTRG.17500.2	miR-340-5p
MSTRG.17500.2	miR-764-5p

MSTRG.17500.2	miR-7056-5p
MSTRG.17500.2	miR-298-5p
MSTRG.17500.2	miR-6975-3p
MSTRG.17500.2	miR-1188-5p
MSTRG.17500.2	miR-6899-3p
MSTRG.17500.2	miR-331-3p
MSTRG.17500.2	miR-24-3p
MSTRG.17500.2	miR-3098-3p
MSTRG.17500.2	miR-6959-5p
MSTRG.17500.2	miR-6998-3p
MSTRG.17500.2	miR-1198-5p
MSTRG.17500.2	miR-3106-3p
MSTRG.17500.2	miR-6903-5p
MSTRG.17500.2	miR-365-2-5p
MSTRG.17500.2	miR-365-1-5p
MSTRG.17500.2	miR-421-5p
MSTRG.17500.2	miR-666-3p
MSTRG.17500.2	miR-6908-5p
MSTRG.17500.2	miR-511-3p
MSTRG.17500.2	miR-539-5p
MSTRG.17500.2	miR-3057-5p
MSTRG.17500.2	miR-139-5p
MSTRG.17500.2	miR-450b-5p
MSTRG.17500.2	miR-6918-5p
MSTRG.17500.2	miR-466i-5p
MSTRG.17500.2	miR-669c-3p
MSTRG.17500.2	miR-466d-5p
MSTRG.17500.2	miR-466k
MSTRG.17500.2	miR-6903-3p
MSTRG.17500.2	miR-7116-3p
MSTRG.17500.2	miR-669m-5p
MSTRG.17500.2	miR-466m-5p
MSTRG.17500.2	miR-466j
MSTRG.17500.2	miR-466h-5p
MSTRG.17500.2	miR-223-5p
MSTRG.17500.2	miR-1187
MSTRG.17500.2	miR-466p-5p
MSTRG.17500.2	miR-466a-5p
MSTRG.17500.2	miR-466e-5p
MSTRG.17500.2	miR-466n-5p
MSTRG.17500.2	miR-466o-3p
MSTRG.17500.2	miR-466m-3p
MSTRG.17500.2	miR-7116-5p
MSTRG.17500.2	miR-669k-5p

MSTRG.17500.2	miR-466l-3p
MSTRG.17500.2	miR-199b-3p
MSTRG.17500.2	miR-199a-3p
MSTRG.17500.2	miR-5125
MSTRG.17500.2	miR-6902-5p
MSTRG.17500.2	miR-7042-5p
MSTRG.17500.2	miR-17-3p
MSTRG.17500.2	miR-7117-5p
MSTRG.17500.2	miR-3473e
MSTRG.17500.2	miR-3473b
MSTRG.17500.2	miR-6979-3p
MSTRG.17500.2	miR-6944-3p
MSTRG.17500.2	miR-20b-3p
MSTRG.17500.2	miR-3083-5p
MSTRG.17500.2	miR-3059-5p
MSTRG.17500.2	miR-7240-5p
MSTRG.17500.2	miR-5101
MSTRG.17500.2	miR-3090-3p
MSTRG.17500.2	miR-6940-3p
MSTRG.17500.2	miR-672-3p
MSTRG.17500.2	miR-7073-5p
MSTRG.17500.2	miR-3473a
MSTRG.17500.2	miR-185-5p
MSTRG.17500.2	miR-7675-3p
MSTRG.17500.2	miR-6896-3p
MSTRG.17500.2	miR-6948-5p
MSTRG.17500.2	miR-3110-5p
MSTRG.17500.2	miR-7032-3p
MSTRG.17500.2	miR-7013-5p
MSTRG.17500.2	miR-466e-3p
MSTRG.17500.2	miR-466d-3p
MSTRG.17500.2	miR-466a-3p
MSTRG.17500.2	miR-297c-3p
MSTRG.17500.2	miR-297b-3p
MSTRG.17500.2	miR-297a-3p
MSTRG.17500.2	miR-670-3p
MSTRG.17500.2	miR-7664-3p
MSTRG.17500.2	miR-669m-3p
MSTRG.17500.2	miR-467e-3p
MSTRG.17500.2	miR-467d-3p
MSTRG.17500.2	miR-467c-3p
MSTRG.17500.2	miR-467b-3p
MSTRG.16327.2	miR-466i-5p
MSTRG.16327.2	miR-466k

MSTRG.16327.2	miR-466d-5p
MSTRG.16327.2	miR-6903-3p
MSTRG.16327.2	miR-28c
MSTRG.16327.2	miR-28b
MSTRG.16327.2	miR-23b-3p
MSTRG.16327.2	miR-23a-3p
MSTRG.16327.2	miR-1930-3p
MSTRG.16327.2	miR-3057-5p
MSTRG.16327.2	miR-6959-5p
MSTRG.16327.2	miR-143-5p
MSTRG.16327.2	miR-122-5p
MSTRG.16327.2	miR-448-5p
MSTRG.16327.2	miR-6913-5p
MSTRG.16327.2	miR-5621-5p
MSTRG.16327.2	miR-101b-3p
MSTRG.16327.2	miR-709
MSTRG.16327.2	miR-30f
MSTRG.16327.2	miR-142a-3p
MSTRG.16327.2	miR-7651-5p
MSTRG.16327.2	miR-214-3p
MSTRG.16327.2	miR-5710
MSTRG.16327.2	miR-466f
MSTRG.16327.2	miR-6985-5p
MSTRG.16327.2	miR-1964-5p
MSTRG.14838.1	miR-3084-3p
MSTRG.14838.1	miR-7214-5p
MSTRG.14838.1	miR-672-5p
MSTRG.14838.1	miR-3067-5p
MSTRG.14838.1	miR-377-5p
MSTRG.14838.1	miR-153-5p
MSTRG.14838.1	miR-7671-5p
MSTRG.14838.1	miR-7664-5p
MSTRG.14838.1	miR-346-3p
MSTRG.14838.1	miR-182-5p
MSTRG.1243.32	miR-5101
MSTRG.1243.32	miR-7226-3p
MSTRG.1243.32	miR-709
MSTRG.1243.32	miR-297c-3p
MSTRG.1243.32	miR-297b-3p
MSTRG.1243.32	miR-297a-3p
MSTRG.1243.32	miR-1960
MSTRG.1243.32	miR-466o-3p
MSTRG.1243.32	miR-466m-3p
MSTRG.1243.32	miR-706

MSTRG.1243.32	miR-1b-5p
MSTRG.1243.32	miR-466e-3p
MSTRG.1243.32	miR-466d-3p
MSTRG.1243.32	miR-466a-3p
MSTRG.1243.32	miR-7092-3p
MSTRG.1243.32	miR-3967
MSTRG.1243.32	miR-222-3p
MSTRG.1243.32	miR-221-3p
MSTRG.1243.32	miR-6922-3p
MSTRG.1243.32	miR-466p-3p
MSTRG.1243.32	miR-466c-3p
MSTRG.1243.32	miR-466b-3p
MSTRG.1243.32	miR-7042-5p
MSTRG.1243.32	miR-6985-5p
MSTRG.1243.32	miR-182-3p
MSTRG.1243.32	miR-134-3p
MSTRG.1243.32	miR-7054-5p
MSTRG.1243.32	miR-547-5p
MSTRG.1243.32	miR-484
MSTRG.1243.32	miR-7010-5p
MSTRG.1243.32	miR-181d-5p
MSTRG.1243.32	miR-181c-5p
MSTRG.1243.32	miR-181b-5p
MSTRG.1243.32	miR-181a-5p
MSTRG.1243.32	miR-6913-5p
MSTRG.1243.32	miR-669f-3p
MSTRG.1243.32	miR-669b-3p
MSTRG.1243.32	miR-467a-3p
MSTRG.1243.32	miR-1264-3p
MSTRG.1243.32	miR-5626-3p
MSTRG.1243.32	miR-136-5p
MSTRG.1243.32	miR-181b-2-3p
MSTRG.1243.32	miR-181b-1-3p
MSTRG.1243.32	miR-6942-5p
MSTRG.1243.32	miR-7052-3p
MSTRG.1243.32	miR-6996-5p
MSTRG.1243.32	miR-30e-3p
MSTRG.1243.32	miR-30d-3p
MSTRG.1243.32	miR-30a-3p
MSTRG.1243.32	miR-669c-3p
MSTRG.1243.32	miR-7027-5p
MSTRG.1243.32	miR-362-3p
MSTRG.1243.32	miR-329-3p
MSTRG.1243.32	miR-146a-3p

MSTRG.1243.32	miR-466f-3p
MSTRG.1243.32	miR-383-3p
MSTRG.1243.32	miR-6946-3p
MSTRG.1243.32	miR-6999-5p
MSTRG.1243.32	miR-504-3p
MSTRG.1243.32	miR-6958-3p
MSTRG.1243.32	miR-3100-5p
MSTRG.1243.32	miR-30c-2-3p
MSTRG.1243.32	miR-30c-1-3p
MSTRG.1243.32	miR-676-5p
MSTRG.1243.32	miR-7073-5p
MSTRG.1243.32	miR-18a-5p
MSTRG.1243.32	miR-7116-3p
MSTRG.1243.32	miR-7b-5p
MSTRG.1243.32	miR-7a-5p
MSTRG.1243.32	miR-187-5p
MSTRG.1243.32	miR-3971
MSTRG.1243.32	miR-1194
MSTRG.1243.32	miR-7669-5p
MSTRG.1243.32	miR-7051-5p
MSTRG.1243.32	miR-7015-5p
MSTRG.1243.32	miR-669m-3p
MSTRG.1243.32	miR-467e-3p
MSTRG.1243.32	miR-467d-3p
MSTRG.1243.32	miR-467c-3p
MSTRG.1243.32	miR-467b-3p
MSTRG.1243.32	miR-466n-3p
MSTRG.1243.32	miR-669i
MSTRG.1243.32	miR-7115-3p
MSTRG.1243.32	miR-702-5p
MSTRG.1243.32	miR-335-3p
MSTRG.1243.32	miR-6902-3p
MSTRG.1243.32	miR-760-5p
MSTRG.1243.32	miR-501-5p
MSTRG.1243.32	miR-7661-3p
MSTRG.1243.32	miR-6903-5p
MSTRG.1243.32	miR-466i-3p
MSTRG.1243.32	miR-6769b-3p
MSTRG.1243.32	miR-141-5p
MSTRG.1243.32	miR-128-3p
MSTRG.1243.32	miR-6539
MSTRG.1243.32	miR-664-3p
MSTRG.1243.32	miR-743b-3p
MSTRG.1243.32	miR-488-3p

MSTRG.1243.32	miR-6983-5p
MSTRG.1243.32	miR-1941-5p
MSTRG.1243.32	miR-551b-5p
MSTRG.1243.32	miR-3068-5p
MSTRG.1243.32	miR-3969
MSTRG.1243.32	miR-6911-5p
MSTRG.1243.32	miR-376b-3p
MSTRG.1243.32	miR-7055-5p
MSTRG.1243.32	miR-744-3p
MSTRG.1243.32	miR-874-3p
MSTRG.1243.32	miR-490-5p
MSTRG.1243.32	miR-7012-5p
MSTRG.1243.32	miR-3572-3p
MSTRG.1243.32	miR-412-3p
MSTRG.1243.32	miR-378a-5p
MSTRG.1243.32	miR-1251-5p
MSTRG.1243.32	miR-7b-3p
MSTRG.1243.32	miR-7a-2-3p
MSTRG.1243.32	miR-152-3p
MSTRG.1243.32	miR-148b-3p
MSTRG.1243.32	miR-148a-3p
MSTRG.1243.32	miR-7668-5p
MSTRG.1243.32	miR-330-5p
MSTRG.1243.32	miR-338-3p
MSTRG.1243.32	miR-1983
MSTRG.1243.32	miR-7081-3p
MSTRG.1243.32	miR-219a-1-3p
MSTRG.1243.32	miR-194-2-3p
MSTRG.1243.32	miR-6984-3p
MSTRG.1243.32	miR-6901-5p
MSTRG.1243.32	miR-5129-5p
MSTRG.1243.32	miR-300-5p
MSTRG.1243.32	miR-877-3p
MSTRG.1243.32	miR-326-3p
MSTRG.1243.32	miR-222-5p
MSTRG.1243.32	miR-365-3p
MSTRG.1243.32	miR-7043-3p
MSTRG.1243.32	miR-193b-5p
MSTRG.1243.32	miR-1981-3p
MSTRG.1243.32	miR-701-3p
MSTRG.1243.32	miR-511-3p
MSTRG.1243.32	miR-7068-3p
MSTRG.1243.32	miR-3090-3p
MSTRG.1243.32	miR-1247-3p

MSTRG.1243.32	miR-539-5p
MSTRG.1243.32	miR-129b-3p
MSTRG.1243.32	miR-6946-5p
MSTRG.1243.32	miR-202-5p
MSTRG.1243.32	miR-483-3p
MSTRG.1243.32	miR-450b-3p
MSTRG.1243.32	miR-450a-1-3p
MSTRG.1243.32	miR-130a-5p
MSTRG.1243.32	miR-495-5p
MSTRG.1243.32	miR-22-5p
MSTRG.1243.32	miR-3082-3p
MSTRG.1243.32	miR-186-5p
MSTRG.1243.32	miR-1291
MSTRG.1243.32	miR-6941-5p
MSTRG.1243.32	let-7f-2-3p
MSTRG.1243.32	miR-6999-3p
MSTRG.1243.32	miR-679-5p
MSTRG.1243.32	miR-6240
MSTRG.1243.32	miR-770-3p
MSTRG.1243.32	miR-350-3p
MSTRG.1243.32	miR-6991-5p
MSTRG.1243.32	miR-1930-5p
MSTRG.1243.32	miR-1668
MSTRG.1243.32	miR-879-5p
MSTRG.1243.32	miR-6985-3p
MSTRG.1243.32	miR-673-5p
MSTRG.1243.32	miR-6918-5p
MSTRG.1243.32	miR-345-3p
MSTRG.1243.32	miR-139-5p
MSTRG.1243.32	miR-24-3p
MSTRG.1243.32	miR-5620-3p
MSTRG.1243.32	miR-7651-5p
MSTRG.1243.32	miR-330-3p
MSTRG.1243.32	miR-298-3p
MSTRG.1243.32	miR-7665-5p

Table S11. Information of RNAs in the L-M-T network based on RNA-seq results.

RNA	Cortex				Hippocampus			
	Log_2Fold Change	*p*-value	*q*-value	Regulation	Log_2Fold Change	*p*-value	*q*-value	Regulation
ENSMUST00000127786	11.46	3.74×10^{-5}	1.57×10^{-6}	up	10.49	5.72×10^{-11}	2.86×10^{-8}	up
ENSMUSG00000098912	-16.56	6.00×10^{-12}	3.79×10^{-9}	down	-1.68	1.49×10^{-5}	2.26×10^{-3}	down
miR-362-3p	0.72	1.01×10^{-6}	1.36×10^{-4}	up	0.15	5.28×10^{-1}	8.87×10^{-1}	up
miR-329-3p	-0.10	4.49×10^{-1}	7.26×10^{-1}	down	-0.21	3.44×10^{-1}	8.21×10^{-1}	down
miR-466i-3p	-0.41	5.69×10^{-1}	1.00	down	1.07	1.07×10^{-1}	1.00	up
miR-669b-5p	1.15	2.46×10^{-2}	1.89×10^{-1}	up	0.59	3.28×10^{-1}	1.00	up
miR-3057-5p	0.25	4.17×10^{-1}	7.17×10^{-1}	up	-0.23	4.95×10^{-1}	8.76×10^{-1}	down
miR-5101	-0.59	6.63×10^{-1}	1.00	down	0.69	6.09×10^{-1}	1.00	up
miR-15a-5p	0.62	9.48×10^{-4}	2.36×10^{-2}	up	0.25	3.21×10^{-1}	7.88×10^{-1}	up
miR-466o-3p	-1.46	7.00×10^{-1}	1.00	down	-1.54	6.09×10^{-1}	1.00	down
miR-466m-3p	-2.33	2.27×10^{-1}	1.00	down	-1.44	7.25×10^{-1}	1.00	down
miR-539-5p	-0.09	7.49×10^{-1}	8.97×10^{-1}	down	-0.05	8.63×10^{-1}	9.69×10^{-1}	down
Clec7a	5.99	2.38×10^{-5}	3.26×10^{-3}	up	7.46	1.10×10^{-5}	1.76×10^{-3}	up
Elovl2	10.03	1.89×10^{-7}	5.62×10^{-5}	up	6.87	2.12×10^{-6}	4.34×10^{-4}	up
Fnbp1	16.14	4.15×10^{-6}	7.74×10^{-4}	up	-16.40	8.16×10^{-20}	1.57×10^{-16}	down
5730455P16Rik	10.51	3.57×10^{-7}	9.41×10^{-5}	up	-11.23	1.29×10^{-8}	4.25×10^{-6}	down
